# Two Roomtemperature‐stable Trications – Triprotonated Triamino‐ and Tricyanobenzene

**DOI:** 10.1002/open.202200049

**Published:** 2022-05-11

**Authors:** Alexander Nitzer, Robert Hübsch, Christoph Jessen, Andreas J. Kornath

**Affiliations:** ^1^ Department of Chemistry and Pharmacy LMU Munich Butenandtstrasse 5–13 81377 Munich Germany

**Keywords:** spectroscopy, superacid, triaminobenzene, trication, tricyanobenzene

## Abstract

Protonation of 1,3,5‐tricyano‐ and 1,3,5‐triaminobenzene was achieved in various superacidic media, resulting in the formation of the respective trinitrilium and triammonium species. Furthermore, the respective *N*‐methyl nitrilium species was synthesized by methylation. Characterization was performed by NMR and vibrational spectroscopy, followed by single‐crystal X‐ray diffraction analyses of selected species. Fourfold protonation of the amine, which would have led to the triammonium arenium species, could not be achieved. Quantum chemical calculations are employed to enable full vibrational assignment as well to quantify charge localization.

## Graphical abstract

**Triprotonation of 1,3,5‐tricyanobenzene and 1,3,5‐triaminobenzene** was achieved in (super)acidic media. The nitrile species was also successfully trimethylated. Characterization of and comparison between the three trications have been carried out by vibrational and NMR spectroscopy as well as by single‐crystal X‐ray diffraction. The influence of protonation or methylation on the systems is evaluated and compared to predictions made by quantum‐chemical calculations.

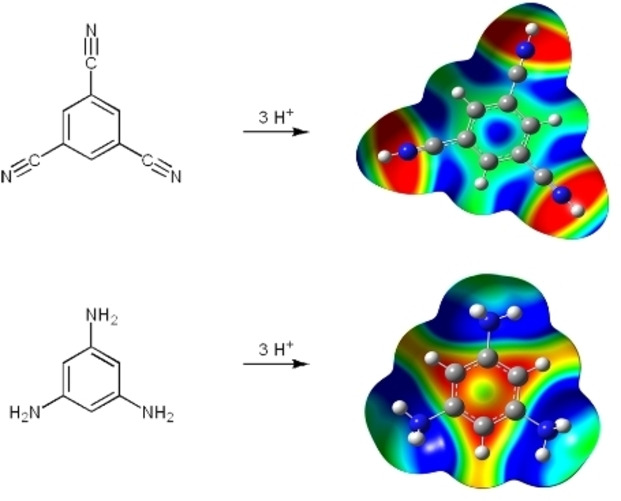

## Introduction

Apart from various carbonium and oxonium cations, numerous studies discuss the protonation of nitrogen‐containing species. Evidently, an enormous variety of ammonium ions are known. Iminium or diazonium cations commonly appear in organic syntheses, most often as intermediates. Even more exotic cations like protonated hydrazoic acid^[1]^ or the N_5_^+^ ion^[2]^ do exist.

**Figure 1:**
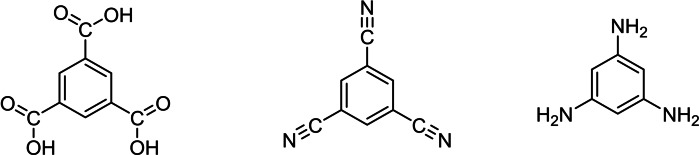
Structures (left to right) of 1,3,5‐tricarboxybenzene, 1,3,5‐tricyanobenzene and 1,3,5‐triaminobenzene.

Out of all aromatic nitrogen‐containing species, the protonation of pyridine is most obvious, it being a key compound in organic chemistry. Protonation of various nitriles,^[3]^ for example of benzonitrile and terephthalonitrile, was easily achieved and led to the respective nitrilium species.^[4]^ With the protonation of 1,3,5‐tricarboxybenzene,^[5]^ we wanted to investigate the protonation of other 1,3,5‐substitued benzenes, but with nitrogen‐containing functional groups (Figure 1).

**Scheme 1:**
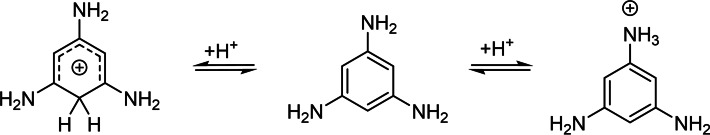
Equilibrium of monoprotonated 1,3,5‐triaminobenzene.

1,3,5‐triaminobenzene is fascinating, as its monoprotonation results in a temperature‐dependent equilibrium (Scheme 1). The σ‐complex (arenium ion) is energetically favored, while the mono‐ammonium species is entropically favored.^[6]^

As the basic amine moieties should be readily protonated in superacidic media, we intended to investigate whether it would be still possible to also protonate the aromatic ring. Benzene itself can be protonated in sufficient superacidic media,^[7]^ so 1,3,5‐triaminobenzene is a candidate for a fourfold protonation. 1,3,5‐tricyanobenzene has a basic site at each nitrile group where protonation should occur first. After triprotonation, enough basicity in the ring could remain to enable fourfold protonation.

## Results and Discussion

**Scheme 2:**
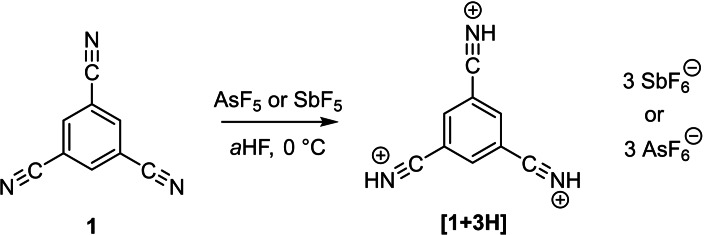
Protonation of 1,3,5‐tricyanobenzene.

1,3,5‐tricyanobenzene is readily protonated at 0 °C in the superacidic media HF/SbF_5_ and HF/AsF_5_ (Scheme 2).

The starting material was dissolved at only about 0 °C in *a*HF (anhydrous hydrogen fluoride) and reacted with the respective superacidic mixtures, resulting in the immediate formation of yellow to colorless solids. Precipitating already at −20 °C, the salts were characterized after removal of the solvent at −78 °C.

As methylation of nitriles^[8]^ is easily possible by means of using the system CH_3_F‐SO_2_‐MF_5_ (M=Sb, As),^[9]^ we intended to investigate the differences in electronic properties of the protonated and methylated derivative.

**Scheme 3:**
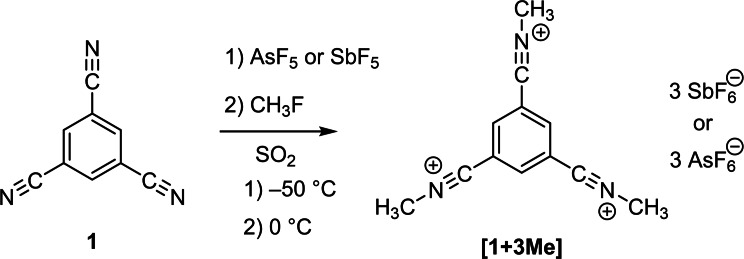
Methylation of 1,3,5‐tricyanobenzene.

Methylation of 1,3,5‐tricyanobenzene was achieved by adding methyl fluoride to frozen antimony or arsenic pentafluoride and reacting the mixture at −50 °C in sulfur dioxide (Scheme 3). After freezing the solution to −196 °C, 1,3,5‐tricyanobenzene was added and the solution was allowed to warm to 0 °C. The in situ formed [SO_2_CH_3_][MF_6_] (M=Sb or As) acts as methylating agent,^[9]^ which reacts with the nitrile. A simultaneous dissolution and homogenization with the nitrile at 0 °C allowed for the formation of the *N*‐methyl nitrilium salts. Cooling of the reaction mixture leads to the precipitation of the yellowish salts at −40 °C, which are then characterized after removing the solvent at −60 °C.

The room‐temperature‐stable salts of both **[1+3H]** and **[1+3Me]** were first characterized by Raman spectroscopy. A slight blue‐shift of the C≡N stretching vibration is the first indication of protonation and an even larger blue‐shift a proof of methylation, without considering the vibrations assigned to the anions. Furthermore, **[1+3H]**[(SbF_6_)_3_] was re‐dissolved in anhydrous hydrogen fluoride at 0 °C and **[1+3Me]**[(SbF_6_)_3_] in sulfur dioxide at −20 °C, respectively, followed by an NMR spectroscopic examination. For unequivocal proof, crystallization enabled isolation of **[1+3H]**[(Sb_2_F_11_)_2_(SbF_6_)] ⋅ 3HF as determined by single‐crystal X‐ray diffraction analysis. The results will be discussed further below.

**Scheme 4:**
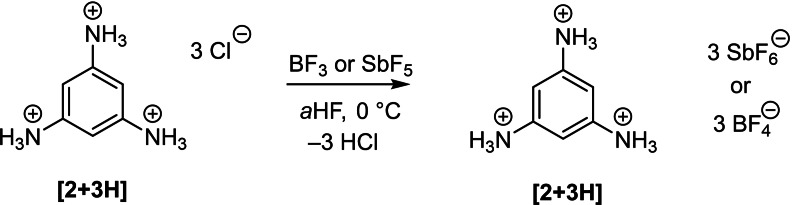
Isolation of triprotonated 1,3,5‐triaminobenzene.

1,3,5‐triaminobenzene hydrochloride was reacted with HF/BF_3_ and HF/SbF_5_ respectively with the starting material already dissolving at −40 °C. Removal of the solvent yielded colorless solids for characterization (Scheme 4).

The room‐temperature‐stable [(SbF_6_)_3_] and [(BF_4_)_3_] species and the starting material were compared by vibrational spectroscopy, showing similarity of the three compounds up to the fingerprint region. Furthermore, comparison of ^14^N NMR data of the starting material in D_2_O and of **[2+3H]**[(SbF_6_)_3_] in *a*HF revealed the same cationic species. Crystallization of the triammonium species in the system HF/SbF_5_ yielded **[2+3H]**[(SbF_6_)_3_] ⋅ HF as determined by single crystal X‐ray diffraction analysis. The data will be discussed at a later point. No crystal structure of **2** or **[2+3H]** has been reported so far. Even with tenfold stoichiometric amount of SbF_5_, no fourfold protonation was achieved, only polyfluoridoantimonates could be observed.

### X‐ray Crystal Structures

By recrystallization from *a*HF, crystals of both cationic species were grown, which were suitable for single‐crystal X‐ray diffraction. The complete list of bond lengths and additional crystallographic details can be found in the Supporting Information (Chapter 3).

**Figure 2:**
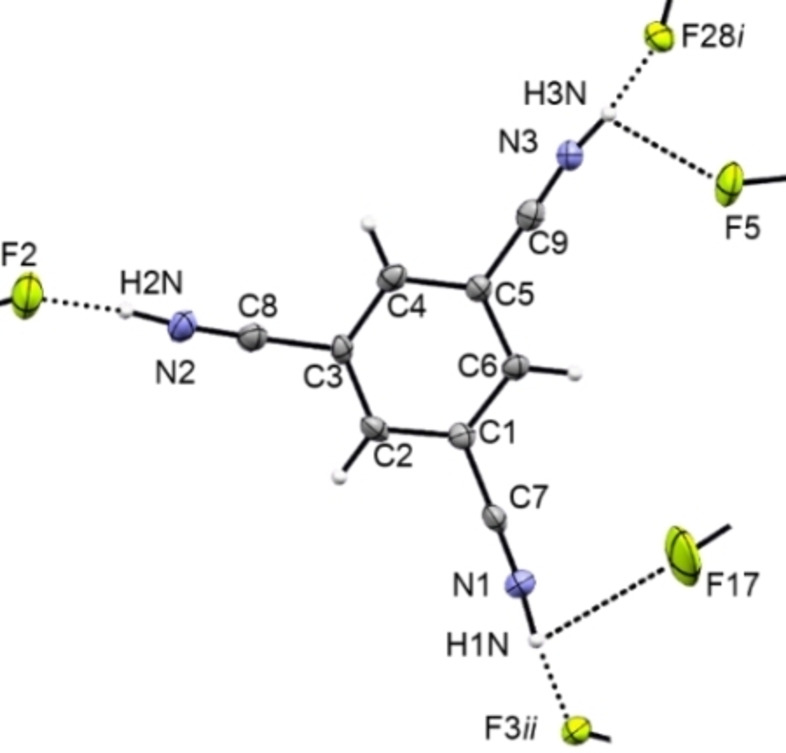
Section of the crystal structure of **[1+3H]**[(Sb_2_F_11_)_2_(SbF_6_)] ⋅ 3HF, view along *a*, displacement ellipsoids at 50 % probability, *i*=x, −1+y, 1+z, *ii*=1−x, 1−y, −z.

**[1+3H]**[(Sb_2_F_11_)_2_(SbF_6_)] ⋅ 3HF crystallizes in the triclinic space group *P* $\overset{-}{1}$ (*Z*=2) (Figure 2). The C≡N distances amount to 1.125(7), 1.135(7) and 1.141(7) Å, similar to those of the starting material and comparable to other nitrilium ions.^[4]^ The C−C bonds adjacent to the CN groups measure 1.424(8), 1.426(8) and 1.442(7) Å and are thus of similar length compared to 1,3,5‐tricyanobenzene.^[10]^ A shortening of these bonds, as it was observed by protonation of 1,3,5‐tricarboxybenzene, where the C−C bonds adjacent to the [COOH_2_] moiety were slightly shortened, is not observed for the nitrilium derivative. The aromatic C−C bonds do not change in length by protonation, measuring 1.382(7) to 1.399(7) Å.

**Figure 3:**
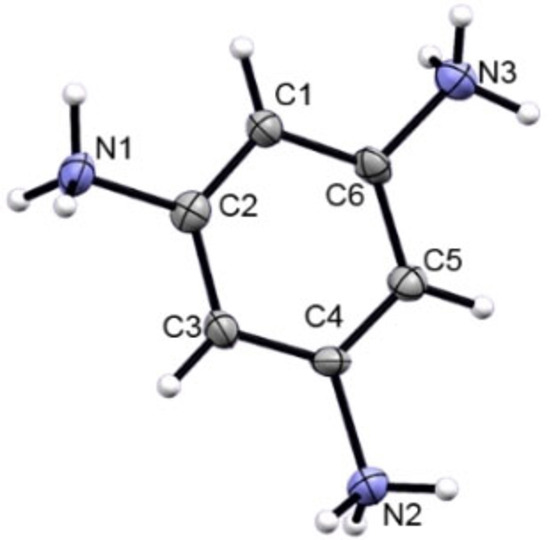
Cation from the asymmetric unit of **[2+3H]**[(SbF_6_)_3_] ⋅ HF, view along *a*, displacement ellipsoids at 50 % probability.

Triprotonated 1,3,5‐triaminobenzene, **[2+3H]**[(SbF_6_)_3_] ⋅ HF crystallizes in the monoclinic space group *P*2_1_/*n* (*Z*=4) (Figure 3). The CN bonds measure 1.484(9), 1.480(8) and 1.459(8) Å and are in the range of typical C−N single bonds.^[11]^ In the crystal structure of protonated 2,4,6‐tri‐*tert*‐butylaniline (TBA),^[12]^ a C−N distance of 1.473(5) Å was found, a similar length as in the here presented ammonium species. The crystal structure of aniline hydrobromide measured at 70 °C exhibits a C−N bond distance of 1.47 Å.^[13]^ In the respective hydrochloride compound, a C−N bond length of 1.35 Å is present, a significant decrease in length compared to the afore‐mentioned species.^[14]^ The authors note that in various other ammonium species, be they aromatic, aliphatic or heterocyclic, a similar observation of a short C−N distance was made.^[14]^ Thus, a significant influence of the counterion is observed, whilst the amount of charge on the cation is not of importance. The temperature has also only a marginal influence on the C−N bond length.

### NMR Spectroscopy

The complete list of NMR spectroscopic data is given in the Supporting Information (Chapter 4). Sulfur dioxide was used as the solvent for NMR spectroscopical analyses of **1** and **[1+3Me]** at 26 °C and −20 °C, respectively, while **[1+3H]** was characterized at 0 °C in *a*HF. An overview of chemical shifts is given in Table 1. Aromatic protons slightly shift depending on solvent and temperature. Still, a moderate deshielding of these resonances from starting material to nitrilium ion is observed. The NH protons display narrow signals for **[1+3H]** at 9.84 ppm, while the methyl protons are observed at 5.44 ppm for **[1+3Me]**. Solvent effects and temperature do not play a major role for ^13^C NMR shifts. Only upon protonation and methylation do the ^13^C resonances shift significantly. The C−H aromatic carbons are shifted downfield, while the **C−**CN carbons and nitrilium carbons are shifted upfield. The *N*‐methyl nitrilium species displays the methyl group at 34.0 ppm. The change from nitrile to nitrilium is detected in the ^14^N NMR spectrum as the nitrogen resonance shifts downfield.

**Table 1:** ^1^H, ^13^C and ^14^N NMR shifts of **1**, **[1+3H]** and **[1+3Me]**.^[a]^

	**1** ^[b]^	**[1+3H]** ^[c]^	**[1+3Me]** ^[d]^
δ ^1^H (C−**H**)	8.72	9.03	10.49
δ ^1^H (CN**H** ^+^)		9.84	
δ ^1^H (N−**C**H_3_)			5.44
δ ^13^C (**C**−H)	141.1	150.4	150.1
δ ^13^C (**C**−CN)	113.8	109.1	111.3
δ ^13^C (**C≡**N)	111.2	100.1	100.9
δ ^13^C (N−**C**H_3_)			34.0
δ ^14^N	−238.8	−214.7	−215.1

[a] All shifts in ppm; [b] in SO_2_ at 26 °C; [c] in *a*HF at 0 °C; [d] in SO_2_ at −20 °C.

**Figure 4:**
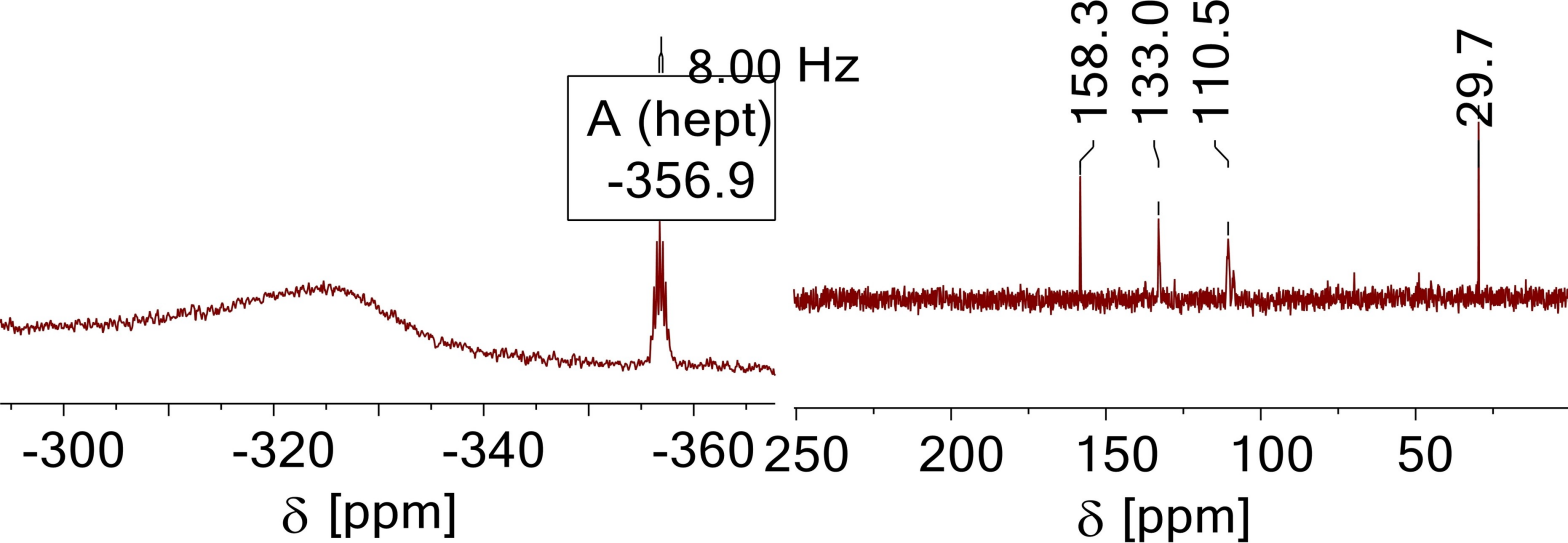
^14^N NMR (left) and ^13^C NMR spectra (right) of **[2+3H]**[Cl_3_] in D_2_O, shifts in ppm.

NMR spectroscopy of 1,3,5‐triaminobenzene hydrochloride in D_2_O and in *a*HF showed the presence of the ammonium species to be prevalent, as the presence of a ND_3_^+^ group is detected in the ^14^N NMR spectrum (Figure 4; see Supporting Information, Chapter 4.5, for a detailed explanation). The previously reported equilibrium between arenium and mono ammonium species^[6]^ was also detected. Beside the two aromatic signals in the ^13^C NMR spectrum, other signals, the strongest at 29.7 ppm and 158.3 ppm, are observed (Figure 4). The amount of hydrochloric acid in D_2_O thus acidifies the NMR sample sufficiently to induce the equilibrium. For the sample in *a*HF, only two signals in the ^13^C NMR spectrum are detected (Table 2). The triammonium species thus can be assumed to be present in the solution, as a mono‐ or diammonium species would exhibit more resonances. An equilibrium between arenium and ammonium ion is no longer possible, when at least diprotonation, in any case triprotonation of **2** is achieved. A fourfold protonation must thus also be excluded.

**Table 2:** ^1^H, ^13^C and ^14^N NMR shifts of **[2+3H]**.^[a]^

	**[2+3H]**[Cl_3_] in D_2_O	**[2+3H]**[(SbF_6_)_3_] in *a*HF
δ ^1^H (C−**H**)	7.03	7.55
δ ^13^C (**C**−H)	110.5	122.0
δ ^13^C (**C**−NH_3_)	133.0	132.1
δ ^14^N	−356.9	−333.5

[a] all shifts in ppm, measured at 25 or 26 °C.

### Vibrational Spectroscopy

Protonation or methylation of **1** is detectable by IR and Raman spectroscopy. For clarity, only selected frequencies of cations from respective hexafluoridoantimonates are depicted in Table 3. Most obvious are the respective blue‐shifts of the C≡N stretching vibration of 31 cm^−1^ (IR) and 64 cm^−1^ (Raman) upon protonation and of 148 cm^−1^ (IR) and 157 cm^−1^ (Raman) upon methylation. The ring‐breathing mode at 974 cm^−1^ in **1** only shifts slightly by protonation or methylation. The intensity of the respective line, however, is intensified, especially by protonation. For **[1+3H]**, the NH stretching vibrations occur as a broad band at 3437 cm^−1^ in the IR spectrum. The C−N stretching vibration of **[1+3Me]** is observed at 910 cm^−1^ (IR) and 905 cm^−1^ (Raman), furthermore the deformation of the methyl group appears at 1429 cm^−1^ in the IR spectrum. The complete list of vibrational data is provided in the Supporting Information (Chapter 2).

**Table 3:** Selected experimental vibrational frequencies [cm^−1^] of **1**, **[1+3H]** and **[1+3Me]**; assignments based on calculated vibrational frequencies [cm^−1^].^[a]^

Assignment	**1**	**[1+3H]**[(SbF_6_)_3_]	**[1+3Me]**[(SbF_6_)_3_]
ν (C≡N)	2249 (m) / 2248 (100)	2280 (m) / 2312 (70)	2397 (m) / 2405 (100)
ring breathing (only Raman)	974 (3)	1000 (72)	998 (16)
ν (NH) (only IR)		3437 (m, broad)	
δ (CH_3_) (only IR)			1429 (m)
ν (C−N)			910 (m) / 905 (11)

[a] Calculated on B3LYP/6–311 g++(3d2 f,3p2d) level of theory, IR intensities in km/mol, Raman intensities in Å^4^/u; abbreviations for IR intensities: m – medium.

For triprotonated 1,3,5‐triaminobenzene, selected vibrations are listed in Table 4, showing the similarity of hydrochloride compound compared to **[2+3H]**[(BF_4_)_3_] and **[2+3H]**[(SbF_6_)_3_]. The ring‐breathing mode appears at the same wavenumber for the hydrochloride as well as for the [SbF_6_] and [BF_4_] salts. Furthermore, the deformation vibration of the NH_3_ group appears for all three salts at a similar position in the IR spectra, that is, at around 1650 cm^−1^. A deformation mode of a NH_2_ group would appear at a lower wavenumber, thus excluding the presence of an unprotonated amino group in any cation.^[15]^

**Table 4:** Selected experimental vibrational frequencies [cm^−1^] of **[2+3H]** with respective anions, assignments based on respective calculated vibrational frequencies [cm^−1^].^[a]^

Assignment	**[2+3H]**[(Cl)_3_]	**[2+3H]**[(SbF_6_)_3_]	**[2+3H]**[(BF_4_)_3_]
ring breathing (only Raman)	1007 (100)	1008 (56)	1008 (100)
δ (NH_3_) (only Raman)	1635 (25)	1651 (23)	1649 (51)
ρ (NH_3_) (only IR)		1028 (vs)	1028 (m)

[a] Calculated at the B3LYP/6–311 g++(3d2 f,3p2d) level of theory, IR intensities in km mol^−1^, Raman intensities in Å^4^/u; abbreviations for IR intensities: vs. – very strong, m – medium.

### Quantum‐Chemical Calculations

For **1**, **[1+3H]** and **[1+3Me]** as well as **2** and **[2+3H]**, quantum‐chemical calculations are employed, using DFT at the B3LYP/6–311G++(3d2 f, 3p2d) level of theory. GaussView 6.0 was used for visualization, Gaussian16 for calculations.^[16]^ Vibrational frequencies were computed after the optimization, selected observed and calculated vibrations of 1,3,5‐tricyanobenzene as well as its triprotonated and trimethylated derivatives are listed in Table 5. DFT in general, hybrid functionals especially, are suitable for prediction of vibrational frequencies.^[17]^ The C≡N stretching vibration is calculated to be at higher wavenumbers, which is explainable by the gas‐phase‐optimization of the computed molecule, leading to overall higher calculated wavenumbers. The two predicted modes are too close together to be observed individually. The blueshift of the C≡N stretching is also expected by the employed calculations, although the overestimation of the vibration(s) becomes smaller. The two modes which are predicted diverge compared to **1**, which is observable by the broadening of the assigned Raman line. The shift of the C≡N stretching mode by methylation is almost exactly calculated, the two calculated modes being only 1 and 4 cm^−1^ blueshifted compared to the observed one. The ring‐breathing mode of **1** is overestimated by the DFT method; in **[1+3H]** and **[1+3Me]** the difference is smaller. The calculations predict a slight redshift of the mode, while a slight blueshift is observed. The calculation predicts the C−N stretching vibration in the methylated compound to appear by almost 50 cm^−1^ lower. This discrepancy may be due to a stabilizing effect occurring between the methyl group and the nitrogen, more on that matter will be elaborated when discussing NPA charges.

**Table 5:** Selected experimental and *[calculated]* Raman frequencies [cm^−1^] of **1**, **[1+3H]** and **[1+3Me]**, intensities given in round brackets.^[a]^

Assignment	**1**	**[1+3H]**[(SbF_6_)_3_]	**[1+3Me]**[(SbF_6_)_3_]
ν (C≡N)	2248 (100) [2280 (877)] [2278 (586)]	2312 (70) [2331 (767)] [2326 (638)]	2405 (100) [2409 (2759)] [2406 (2129)]
ring‐breathing	974 (3) [1017 (76)]	1000 (72) [1003 (70)]	998 (16) [1008 (87)]
ν (C−N)			905 (11) [847 (137)]

[a] Calculated at the B3LYP/6–311 g++(3d2 f,3p2d) level of theory, Raman intensities in Å^4^/u, observed intensities scaled to 100.

The ring breathing mode of **[2+3H]** is observed at 1007 cm^−1^ in the hydrochloride, at 1008 cm^−1^ in the hexafluoridoantimonate and tetrafluoridoborate and predicted at 1021 cm^−1^, fitting closely to the experimental data.

The computed bond distances for **1**, **[1+3H]** and **[2+3H]** mostly agree with the measured ones. Selected distances are listed in Table 6. The gas phase optimization leads to similar bond lengths of each type of bond, for example all CN bonds being calculated to be equally long. The measured bond lengths in the cations are under the influence of various solid‐state effects such as hydrogen bonding. A (slight) deviation from the computed lengths can thus be explained. Nevertheless, most experimentally observed bond distances do fit to the calculated lengths, in both cationic species they are at most slightly overestimated. Only for **1**, where not hydrogen bonding, but π‐π interactions are mainly contributing to the packing, the difference between calculation and observation is apparent. The C≡N and CC (ring) bond lengths are slightly overestimated even when considering standard deviations of measured lengths, the C−C(N) distance is predicted to be longer.

**Table 6:** Selected observed and *[calculated]* bond lengths (Å) of **1**, **[1+3H]** and **[2+3H]**.^[a]^

Bond	**1**	**[1+3H]**	Bond	**[2+3H]**
d (C≡N)	1.135(3) [1.166]	1.135(7) [1.148]		
d (C–CN)	1.444(3) [1.432]	1.426(8) [1.436]	d (C−N)	1.480(8) [1.502]
d (CC) (ring)	1.387(3) [1.404]	1.390(6) [1.410]	d (CC) (ring)	1.375(7) [1.400]

[a] Calculated at the B3LYP/6–311 g++(3d2 f,3p2d) level of theory, calculated bond lengths rounded to the third decimal place.

**Scheme 5:**
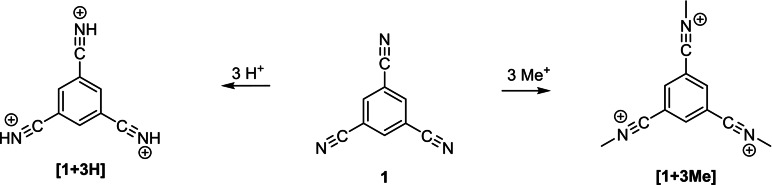
Synthesized nitrilium species derived from 1,3,5‐tricyanobenzene.

To locate charges and to comprehend the stabilization of the cations, NPA charges were calculated at the described level of theory. 1,3,5‐tricyanobenzene easily forms the respective (tri‐)nitrilium species, either by protonation or methylation (Scheme 5).

**Figure 5:**
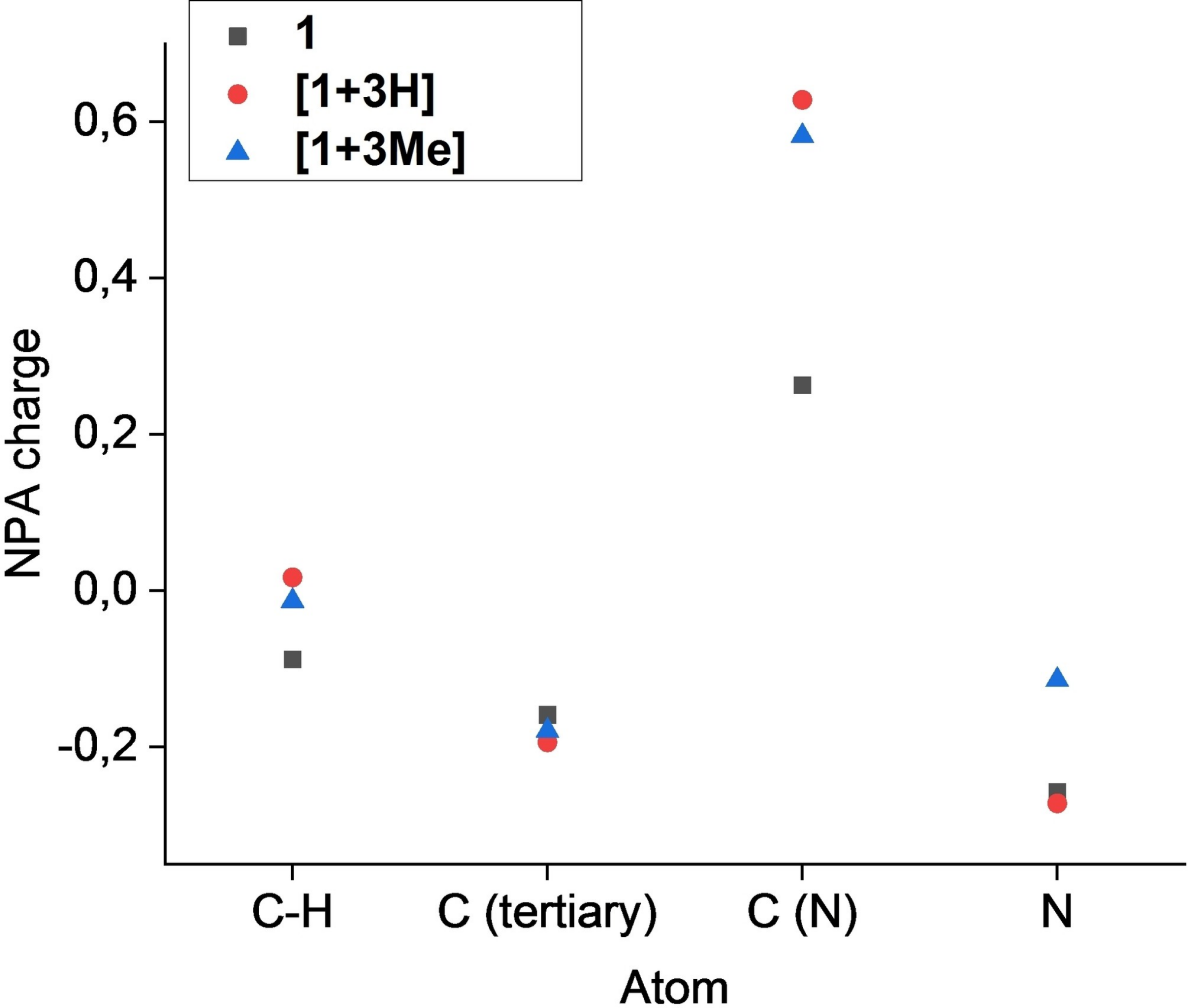
Plot of the NPA charges of the non‐hydrogen atoms of **1**, **[1+3H]** and **[1+3Me]** obtained by DFT calculations at the B3LYP/6‐311G++(3d2 f, 3p2d) level of theory.

As computed by the NPA calculations, the positive charge, emerging either by methylation or protonation, is mostly located at the nitrilium carbon (Figure 5). The NPA charge of that carbon increases from 0.263 in **1** to 0.582 in **[1+3Me]** and 0.628 in **[1+3H]**. The *N*‐methyl nitrilium species exhibits a larger stabilization of the positive charge on the nitrogen compared to the protonated derivative. The nitrogen atoms’ NPA charge increases from −0.258 in **1** to −0.114 in **[1+3Me]**, whereas in **[1+3H]** it even slightly decreases to −0.272. The participation of the aromatic carbons is small but present, with the C−H aromatic carbons losing electron density and the arylic carbons connected to the nitrilium groups gaining it. Lopez and co‐workers^[18]^ studied protonation of various nitriles by theoretical calculations. They showed that depending on which group is attached to the nitrile/nitrilium species, σ‐electron density (alkyl group) or π‐electron density (aryl group) is provided to the nitrile carbon. When analyzing NPA charges of **1**, **[1+3H]** and **[1+3Me]**, this σ‐electron donation is observable. So, the underestimation of the observed C−N stretching vibration in the methylated compound may also be explained.

**Figure 6:**
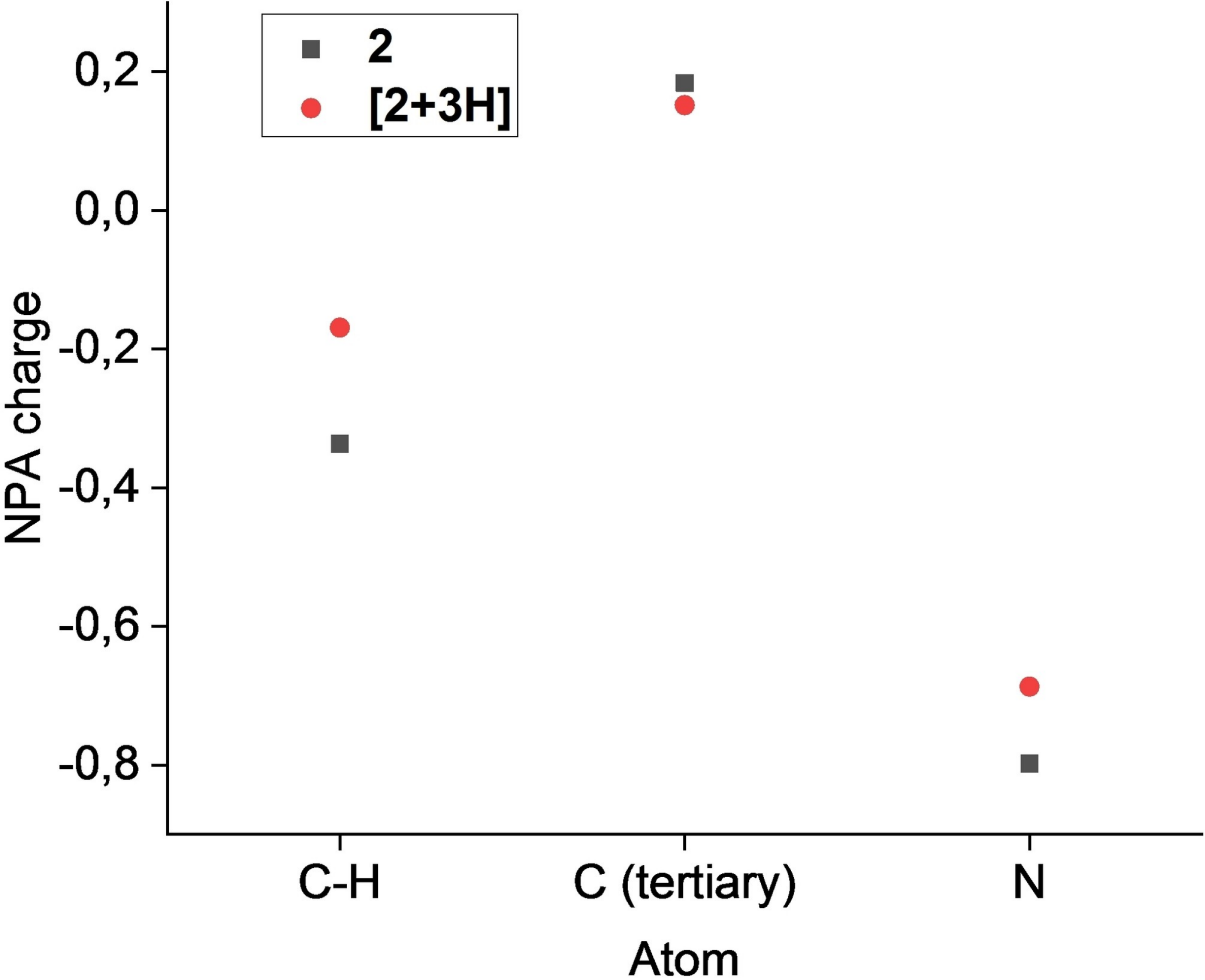
Plot of the NPA charges of the non‐hydrogen atoms of **2** and **[2+3H]** obtained by DFT calculations at the B3LYP/6‐311G++(3d2 f, 3p2d) level of theory.

Protonation of 1,3,5‐triaminobenzene shows a similar change of NPA charges in the aromatic system as calculated for 1,3,5‐tricyanobenzene (Figure 6). As the protonated site is directly connected to the ring, thus “lacking” a carbon atom compared to **1**, the positive charge is located on the sp^3^‐hybridized nitrogen. Still, the C−H aromatic carbons show a larger increase in NPA charge compared to **[1+3H]**.

^13^C NMR shieldings were calculated for the here discussed species using the GIAO method^[19]^ at the B3LYP/6−311G++(3d2 f, 3p2d) level of theory. A comparable method was used by Vazquez to compute ^13^C chemical shifts, which were in good agreement with experimental data.^[20]^ The observed NMR shifts as well as the calculated ones are listed in Table 7. The before‐mentioned charge stabilization occurring in **[1+3H]** and **[1+3Me]** is also expected by the computed values. The greatest deviation from calculated shieldings is found for the aromatic carbons in **[1+3H]**, the calculations expecting a further deshielded value, by 8 ppm from the observed one.

**Table 7:** Selected measured and *[calculated]* ^13^C NMR shieldings [ppm] of **1**, **[1+3H]**, **[1+3Me]** and **[2+3H]**.^[a]^

Atom	**1** in SO_2_ @RT	**[1+3H]** in HF @0 °C (Δ to **1**)	**[1+3Me]** in SO_2_ @−20 °C (Δ to **1**)	Atom	**[2+3H]** in aHF @RT
**C**N	111.2 [118]	100.1 (−11.1) [100]	100.90 (−10.3) [102]		
**C**CN	113.8 [117]	109.1 (−4.7) [109]	111.3 (−2.5) [112]	**C**N	132.1 [134]
**C**‐H	141.1 [141]	150.4 (+9.3) [158]	150.1 (+9.0) [153]	**C**‐H	122.0 [128]
N‐**C**H_3_			34.0 [35]

[a] Calculated on B3LYP/6–311 g++(3d2 f,3p2d) level of theory with the GIAO method, calculated ^13^C shielding with TMS as reference, shielding rounded to a full number.

**Figure 7:**
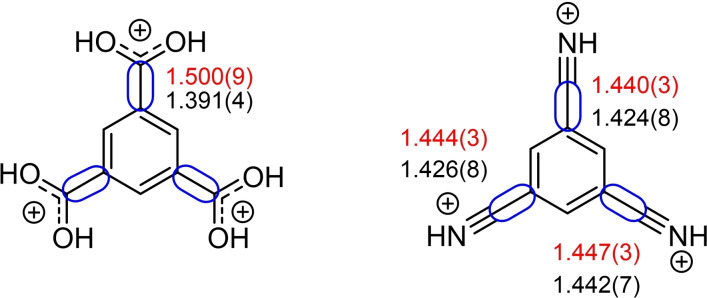
Triprotonated 1,3,5‐tricarboxybenzene (left)^[5]^ and 1,3,5‐tricyanobenzene (right) with selected bond lengths (Å) of the neutral compound (red) and triprotonated species (black).

The C(OH)_2_^+^ moiety of the previously reported triprotonated 1,3,5‐tricarboxybenzene seems to interact with the aromatic ring to a greater extent compared to the CNH^+^ moiety of **[1+3H]**. The C−C bond between the protonated group and the aromatic ring in triprotonated 1,3,5‐tricarboxybenzene decreases slightly in length compared to the starting material. This observation is not made for 1,3,5‐tricyanobenzene (bonds depicted in blue boxes, Figure 7). The sp‐hybridized CN carbon has more s‐orbital character compared to the sp^2^‐hybridized COOH carbon, leading to a short C−C(N) bond, which is already present in **1**. A slight increase in CC bond strength occurs in the nitrilium species, found by Raman spectroscopy. The stretching mode of the C−C bond blue‐shifts from 1285 cm^−1^ in **1** to 1293 cm^−1^ in **[1+3H]** and to 1311 cm^−1^ in **[1+3Me]**.

The formation of a triammonium arenium ion of **2** could not be achieved. The stability of the ammonium species would suggest sufficient remaining basicity of the benzene moiety. Still, too much charge seems to be concentrated on one cation, ruling out fourfold protonation.

## Conclusion

For the first time, the synthesis of room‐temperature‐stable *N*‐H and *N*‐methyl trinitrilium salts of 1,3,5‐tricyanobenzene, together with the isolation of novel 1,3,5‐triammonium benzene salts, was achieved. The triprotonated nitrile has its positive charges located mainly on the nitrilium carbons, while the methylated species transfers more of the positive charge on the nitrogens due to σ‐electron donation. The influence on the C−H aromatic carbons is considerable for both nitrilium species; in the case of the triammonium species, these carbons are charged in the same magnitude as the nitrogen atoms. Comparison with the previously reported protonation of 1,3,5‐tricarboxybenzene shows that the delocalization of positive charge highly depends on the functional group attached to the ring. A carboxonium group is better stabilized by the aromatic system than a nitrilium group.

## Experimental Procedures

**Caution**! Avoid contact with any of these compounds. Hydrolysis might form HF, which burns skin and causes irreparable damage.

### Apparatus and Materials

Standard Schlenk technique with a stainless steel vacuum line was used to perform all reactions. All reactions in superacidic media were carried out in FEP/PFA reactors closed with a stainless steel valve. HF was dried with F_2_ prior to use. Raman spectra were recorded on a Bruker MultiRAM FT‐Raman spectrometer with Nd:YAG laser excitation (λ=1064 nm). For Raman measurements, samples of products were transferred into a cooled glass cell, which were evacuated afterwards. The starting materials were transferred into NMR tubes and measured at room temperature. IR spectra were recorded with a Vertex‐80 V FTIR spectrometer. Samples were placed on a CsBr single‐crystal plate within a cell, which was cooled for the compounds not stable at room temperature. NMR spectra were recorded on a Jeol ECX400 NMR instrument. The spectrometer was externally referenced to CFCl_3_ for ^19^F, CH_3_NO_2_ for ^14^N and to tetramethylsilane for ^1^H and ^13^C NMR spectra. The spectra were recorded inside 4 mm FEP NMR tube inliners. Acetone‐d_6_ was employed for external shimming when *a*HF or SO_2_ were used as solvents for the respective compounds. The starting materials were measured in 6 mm glass NMR tubes. The NMR samples were prepared by (re‐)dissolving the respective protonated or methylated compound at the designated measuring temperature in *a*HF or SO_2_ and transferring the solution into a 4 mm FEP NMR tube inliner. The inliner was then frozen and flame sealed. The low‐temperature X‐ray diffraction was performed with an Oxford X‐Calibur3 equipped with a Kappa CCD detector, operating with Mo‐K_α_ radiation (λ=0.71073 Å) and a Spellman generator (voltage 50 kV, current 40 mA).

Deposition Number(s) 2085061 for **[1+3H]**[(Sb_2_F_11_)(SbF_6_)_2_] ⋅ 3HF and 2085062 for **[2+3H]**[(Sb_2_F_11_)(SbF_6_)_2_]⋅HF contain the supplementary crystallographic data for this paper. These data are provided free of charge by the joint Cambridge Crystallographic Data Centre and Fachinformationszentrum Karlsruhe Access Structures service.

## General Computational Procedures

Quantum‐chemical calculations were done using Gaussian16 with the integrated NBO 3.0 package. GaussView 6.0 was used for visualization. All structures were computed by DFT at the B3LYP/6‐311G++(3d2 f,3p2d) level of theory. First, each structure was optimized. Computation of frequencies or of NMR shieldings was done after optimization, the latter using the GIAO method. NPA charges were calculated using the integrated NBO 3.0 software at the described level of theory. For more details, see the Supporting Information.

### General Procedure

In a typical experiment for protonation, the Lewis acid and *a*HF were condensed into an FEP reactor at −196 °C. The mixture was reacted at −40 °C for 15 minutes and frozen to −196 °C. The 1,3,5‐triamino‐ or 1,3,5‐tricyanobenzene (∼0.2.–0.5 mmol) was added under constant N_2_‐flow. The complete mixture was reacted at the before‐mentioned temperature. The solution was cooled down to −78 °C and the solvent was removed *in vacuo*. Some material of the obtained colorless salts was used for Raman spectroscopy, the rest was redissolved in *a*HF. A part of the solution was transferred into an FEP NMR tube, the rest was used to grow crystals suitable for single‐crystal X‐ray diffraction.

In a typical experiment for methylation, the Lewis acid, methyl fluoride and SO_2_ were condensed into an FEP reactor at −196 °C. The mixture was reacted at −50 °C for 15 min and frozen to −196 °C. 1,3,5‐tricyanobenzene (∼0.2–0.5 mmol) was added under constant N_2_ flow. The complete mixture was reacted at 0 °C. The solution was cooled down to −60 °C and the solvent was removed *in vacuo*. Some material of the obtained colorless salts was used for Raman spectroscopy, the rest was redissolved in SO_2_ for NMR spectroscopy.

For all experimental details, see the Supporting Information.

## Conflict of interest

The authors declare no conflict of interest.

1

## Supporting information

As a service to our authors and readers, this journal provides supporting information supplied by the authors. Such materials are peer reviewed and may be re‐organized for online delivery, but are not copy‐edited or typeset. Technical support issues arising from supporting information (other than missing files) should be addressed to the authors.

Supporting Information

## Data Availability

The data that support the findings of this study are available in the supplementary material of this article.
