# Supplementary material for: Two Roomtemperature‐stable Trications – Triprotonated Triamino‐ and Tricyanobenzene
Source: ChemistryOpen. 2022 May 11;11(5):e202200049. doi: 10.1002/open.202200049 (PMC9092289; doi:10.1002/open.202200049)
Supplement: Supplementary file 1 — Supporting Information [file OPEN-11-e202200049-s001.pdf]

ChemistryOpen

Supporting Information

Two Roomtemperature-stable Trications – Triprotonated Triamino- and Tricyanobenzene

Alexander Nitzer, Robert Hübsch, Christoph Jessen, and Andreas J. Kornath*

Contents

1. Experimental Details **5**

2. Vibrational Data **9**

2.1 1,3,5-Tricyanobenzene 9

2.2 [1,3,5-$C_6H_3(CNH)_3$][(MF_6)$_3$] 12

2.3 [1,3,5-$C_6H_3(CNCH_3)_3$][(MF_6)$_3$] 14

2.3 [1,3,5-$C_6H_3(NH_3)_3$] 17

3. Crystal Structures **20**

3.1 [1,3,5-$C_6H_3(CNH)_3$][(SbF_6)(Sb_2F_{11})$_2$] · 3HF 20

3.2 [1,3,5-$C_6H_3(NH_3)_3$][(SbF_6)$_3$] · HF 32

3.3 Comparison of bond lengths 39

4. NMR data **41**

4.1 1,3,5-Tricyanobenzene 41

4.2 [1,3,5-$C_6H_3(CNH)_3$][(SbF_6)$_3$] 44

4.3 [1,3,5-$C_6H_3(CNCH_3)_3$][(SbF_6)$_3$] 47

4.4 Comparison of NMR data of 1,3,5-Tricyanobenzene and its` protonated and methylated derivates 50

4.5 1,3,5-Triammoniumbenzene 51

5. Quantum chemical calculations **55**

5.1 1,3,5-Tricyanobenzene 55

5.2 1,3,5-$C_6H_3(CNH)_3$ 58

5.3 1,3,5-$C_6H_3(CNCH_3)_3$. 61

5.4 1,3,5-Triaminobenzene 64

5.5 1,3,5-$C_6H_3(NH_3)_3$. 67

5.6 Comparison of NPA charges 70

5.7 NMR shieldings 72

6. Literature **73**

List of Figures

Figure 1. Raman and IR spectrum of 1,3,5-Tricyanobenzene 9
Figure 2. Raman (and IR spectra) of [1,3,5-$C_6H_3(CNH)_3$][$(SbF_6)_3$] and [1,3,5-$C_6H_3(CNH)_3$][$(AsF_6)_3$]. 12
Figure 3. Raman and IR spectra of [1,3,5-$C_6H_3(CNCH_3)_3$][$(SbF_6)_3$] and [1,3,5-$C_6H_3(CNCH_3)_3$][$(AsF_6)_3$] 14
Figure 4. Raman (and IR spectra) of [1,3,5-$C_6H_3(NH_3)_3$][Cl_3], [1,3,5-$C_6H_3(NH_3)_3$][$(SbF_6)_3$] and [1,3,5-$C_6H_3(CNH)_3$][$(BF_4)_3$]. 17
Figure 5. Asymmetric unit of [1,3,5-$C_6H_3(CNH)_3$][$(SbF_6)(Sb_2F_{11})_2$] · 3HF 20
Figure 6. Particular hydrogen bonds in [1,3,5-$C_6H_3(CNH)_3$][$(SbF_6)(Sb_2F_{11})_2$] · 3HF 23
Figure 7. Selected hydrogen bonds of [1+3H][$(Sb_2F_{11})_2(SbF_6)$] · 3HF 24
Figure 8. Asymmetric unit of [1,3,5-$C_6H_3(NH_3)_3$][$(SbF_6)_3$] · HF 32
Figure 9. Optimized structure of 1,3,5-Tricyanobenzene 55
Figure 10. NPA charges of 1,3,5-Tricyanobenzene 56
Figure 11. NPA charges of 1,3,5-Tricyanobenzene (coloured). 57
Figure 12. Optimized structure of 1,3,5-$C_6H_3(CNH)_3$. 58
Figure 13. NPA charges of 1,3,5-$C_6H_3(CNH)_3$. 59
Figure 14. NPA charges of 1,3,5-$C_6H_3(CNH)_3$ (coloured) 60
Figure 15. Optimized structure of 1,3,5-$C_6H_3(CNCH_3)_3$. 61
Figure 16. NPA charges of 1,3,5-$C_6H_3(CNCH_3)_3$. 63
Figure 17. NPA charges of 1,3,5-$C_6H_3(CNCH_3)_3$ (coloured) 63
Figure 18. Optimized structure of 1,3,5-Triaminobenzene. 64
Figure 19. NPA charges of 1,3,5-Triaminobenzene. 65
Figure 20. NPA charges of 1,3,5-Triaminobenzene (coloured). 66
Figure 21. Optimized structure of 1,3,5-$C_6H_3(NH_3)_3$. 67
Figure 22. NPA charges of 1,3,5-$C_6H_3(NH_3)_3$. 69
Figure 23. NPA charges of 1,3,5-$C_6H_3(NH_3)_3$ (coloured). 69
Figure 24. NPA charges of 1,3,5-Tricyanobenzene, its` protonated and methylated derivate (R= aromatic core). 70
Figure 25. NPA charges of 1,3,5-Triaminobenzene and its` protonated derivate (R= aromatic core). 71

List of Tables

Table 1. Observed vibrational frequencies and calculated vibrational frequencies of 1,3,5-tricyanobenzene 10

Table 2. Observed vibrational frequencies and calculated vibrational frequencies of [1,3,5-$C_6H_3(CNH)_3$][$(MF_6)_3$] 12

Table 3. Observed vibrational frequencies and calculated vibrational frequencies of [1,3,5-$C_6H_3(CNCH_3)_3$][$(MF_6)_3$] 15

Table 4. Observed vibrational frequencies and calculated vibrational frequencies of [1,3,5-$C_6H_3(NH_3)_3$][Cl_3], [1,3,5-$C_6H_3(NH_3)_3$][$(SbF_6)_3$] and [1,3,5-$C_6H_3(NH_3)_3$][$(BF_4)_3$] 17

Table 5. Selected bond lengths (Å) of [1,3,5-$C_6H_3(CNH)_3$][$(SbF_6)(Sb_2F_{11})_2$] · 3HF. 20

Table 6. Selected bond angles (°) of [1,3,5-$C_6H_3(CNH)_3$][$(SbF_6)(Sb_2F_{11})_2$] · 3HF. 21

Table 7. Particular H-bond lengths (Å) for [1,3,5-$C_6H_3(CNH)_3$][$(SbF_6)(Sb_2F_{11})_2$] · 3HF. 23

Table 8. Selected hydrogen bond lengths of [1+3H][$(Sb_2F_{11})_2(SbF_6)$] · 3HF in Å. 24

Table 9. Data collection and structure refinement for [1,3,5-$C_6H_3(CNH)_3$][$(SbF_6)(Sb_2F_{11})_2$] · 3HF 24

Table 10. Bond lengths (Å) of [1,3,5-$C_6H_3(CNH)_3$][$(SbF_6)(Sb_2F_{11})_2$] · 3HF. 25

Table 11. Bond angles (°) for [1,3,5-$C_6H_3(CNH)_3$][$(SbF_6)(Sb_2F_{11})_2$] · 3HF. 27

Table 12. Anisotropic atomic displacement parameters ($Å^2$) for [1,3,5-$C_6H_3(CNH)_3$][$(SbF_6)(Sb_2F_{11})_2$] · 3HF 30

Table 13. Selected bond lengths (Å) of [1,3,5-$C_6H_3(NH_3)_3$][$(SbF_6)_3$] · HF. 34

Table 14. Data collection and structure refinement for [1,3,5-$C_6H_3(NH_3)_3$][$(SbF_6)_3$] · HF. 34

Table 15. Bond lengths (Å) of [1,3,5-$C_6H_3(NH_3)_3$][$(SbF_6)_3$] · HF 35

Table 16. Bond angles (°) for [1,3,5-$C_6H_3(NH_3)_3$][$(SbF_6)_3$] · HF. 36

Table 17. Anisotropic atomic displacement parameters ($Å^2$) for [1,3,5-$C_6H_3(NH_3)_3$][$(SbF_6)_3$] · HF. 38

Table 18. Comparison of bond lengths of [1,3,5-$C_6H_3(CNH)_3$][$(SbF_6)(Sb_2F_{11})_2$] · 3HF with the starting material[10] and [1,3,5-$C_6H_3(NH_3)_3$][$(SbF_6)_3$] · HF 39

Table 19. Comparison of NMR data of 1,3,5-Tricyanobenzene and its` protonated and methylated derivatives. 50

Table 20. Comparison of NMR data of 1,3,5-Tricyanobenzene and 1,3,5-Triammoniumbenzene (135-TAB) and its` protonated and methylated derivatives with calculated ^{13}C NMR shifts in square brackets.[a] 72

List of Images

Image 1. Crystals of [1,3,5-$C_6H_3(CNH)_3$][$(SbF_6)(Sb_2F_{11})_2$] · 3HF. 22
Image 2. Crystals of [1,3,5-$C_6H_3(NH_3)_3$][$(SbF_6)_3$] · HF inside of a NMR tube. 33
Image 3. Crystals of [1,3,5-$C_6H_3(NH_3)_3$][$(SbF_6)_3$] · HF inside a reactor. 33

1. Experimental Details

Caution! Anhydrous HF, AsF_5, BF_3 and SbF_5 can cause severe burns and contact with the skin must be avoided and the compounds should only be handled in a well-ventilated fume hood. Any of the described formed salts may form HF by hydrolysis.

Materials and apparatus:

All reactions were carried out either in FEP/PFA reactors closed with a stainless steel valve, employing standard Schlenk technique with a stainless steel vacuum line. HF was dried with F_2 prior to use.

Raman spectra were recorded on a Bruker MultiRAM FT-Raman spectrometer with Nd:YAG laser excitation (λ = 1064 nm). For Raman measurements, samples of products were transferred into a cooled glass cell, which were evacuated afterwards. The educts were transferred into NMR tubes and measured at room temperature. IR spectra were recorded with a Vertex-80V FT-IR spectrometer. Samples were placed on a CsBr single-crystal plate within a cell, which was cooled for the compounds not stable at room temperature.

NMR spectra were recorded on a Jeol ECX400 NMR instrument. The spectrometer was externally referenced to $CFCl_3$ for ^{19}F, CH_3NO_2 for ^{14}N and to tetramethylsilane for ^{1}H and ^{13}C NMR spectra. The spectra were recorded inside 4 mm FEP NMR tube inliners. Acetone-d_6 was employed for external shimming when *a*HF or SO_2 was used as solvent for the respective compounds. The starting materials were measured in 9 mm glass NMR tubes. The NMR samples were prepared by (re-)dissolving the respective protonated or methylated compound at the designated measuring temperature in *a*HF or SO_2 and transferring the solution into a 4 mm FEP NMR tube inliner. The inliner was then frozen and flame sealed. For visualization and evaluation MestReNova Version 12.0.2 was employed.[1]

The low-temperature X-ray diffraction was performed with an Oxford X-Calibur3 equipped with a Kappa CCD detector, operating with Mo-K_α radiation (λ = 0.71073 Å) and a Spellman generator (voltage 50 kV, current 40 mA). The program CrysAlisPro 1.171.38.46 (Rigaku OD, 2015)[2] was employed for the data collection and reduction. The structures were solved utilizing SHELXT[3] and SHELXL-2018/3[4] of the WINGX software package.[5] The structures were checked using the software PLATON.[6] The absorption correction was

performed using the SCALE3 ABPSACK multiscan method.[7] Visualization was done by Mercury 2020.2.0.[8]

Computational methods:

All here presented calculations were done using DFT, B3LYP/6-311G++(3d2f,3p2d) level of theory by Gaussian 16.[11] NMR calculations were done using the GIAO method on the described level.

Preparations:

1,3,5-Tricyanobenzene and 1,3,5-Triaminobenzene trihydrochloride were used without further purification. 1,3,5-Tricyanobenzene and 1,3,5-Triaminobenzene trihydrochloride were purchased from abcr. Purity was checked by NMR and Raman spectroscopy.

Synthesis of $[1,3,5\text{-}C_6H_3(CNH)_3][(SbF_6)_3]$:

Antimony pentafluoride (290 mg, 1.39 mmol, 6 eq.) and anhydrous HF (ca. 3 mL) were condensed into an FEP reactor at –196 ° C. The mixture was homogenized at –40 ° C for 15 min. After freezing the solution, 1,3,5-Tricyanobenzene (32.0 mg, 0.21 mmol, 1 eq.) was added under nitrogen atmosphere. Followed by removing the nitrogen from the reaction vessel, the mixture was warmed to room temperature and vigorously mixed until all solid was dissolved. Cooling down to 0 ° C led to formation of slightly yellow crystals (see image 1), the mixture was then cooled down to –78° C, so that excess *a*HF could be removed from the system. $[1,3,5\text{-}C_6H_3(CNH)_3][(SbF_6)_3]$ was obtained as a colorless solid, decomposition at room temperature over some hours was not observed. The crystalline compound was identified as $[1,3,5\text{-}C_6H_3(CNH)_3][(SbF_6)(Sb_2F_{11})_2] \cdot 3HF$.

Synthesis of $[1,3,5\text{-}C_6H_3(CNH)_3][(AsF_6)_3]$:

Arsenic pentafluoride (204 mg, 1.20 mmol, 6 eq.) and anhydrous HF (ca. 3 mL) were condensed into an FEP reactor at –196 ° C. The mixture was homogenized at –40 ° C for 15 min. After freezing the solution, 1,3,5-Tricyanobenzene (31.0 mg, 0.20 mmol, 1 eq.) was

added under nitrogen atmosphere. Followed by removing the nitrogen from the reaction vessel, the mixture was warmed to room temperature and vigorously mixed. Cooling down to 0 ° C led to formation of a colorless solid, the mixture was then cooled down to –78° C, so that excess *a*HF could be removed from the system. [1,3,5-$C_6H_3(CNH)_3$][($AsF_6)_3$] was obtained as a colorless solid, which decomposed at room temperature over some hours.

Synthesis of [1,3,5-$C_6H_3(CNCH_3)_3$][($SbF_6)_3$]:

Antimony pentafluoride (230 mg, 1.06 mmol, 6 eq.), methyl fluoride (36.0 mg, 1.06 mmol, 6 eq.) and sulfur dioxide (ca. 3 mL) were condensed into an FEP reactor at –196 ° C. The mixture was homogenized at –50 ° C for 15 min. After freezing the solution, 1,3,5-Tricyanobenzene (27.0 mg, 0.18 mmol, 1 eq.) was added under nitrogen atmosphere. Followed by removing the nitrogen from the reaction vessel, the mixture was warmed to 0 ° C and vigorously mixed until all solid was dissolved. Cooling down to –40 ° C led to formation of a colorless solid, the mixture was then cooled down to –60° C, so that the sulfur dioxide could be removed from the system. [1,3,5-$C_6H_3(CNCH_3)_3$][($SbF_6)_3$] was obtained as a colorless solid, which decomposed at room temperature over some hours.

Synthesis of [1,3,5-$C_6H_3(CNCH_3)_3$][($AsF_6)_3$]:

Arsenic pentafluoride (513 mg, 3.02 mmol, 6 eq.), methyl fluoride (103 mg, 3.02 mmol, 6 eq.) and sulfur dioxide (ca. 3 mL) were condensed into an FEP reactor at –196 ° C. The mixture was homogenized at –50 ° C for 15 min. After freezing the solution, 1,3,5-Tricyanobenzene (77.0 mg, 0.50 mmol, 1 eq.) was added under nitrogen atmosphere. Followed by removing the nitrogen from the reaction vessel, the mixture was warmed to 0 ° C and vigorously mixed until all solid was dissolved. Cooling down to –40 ° C led to formation of a colorless solid, the mixture was then cooled down to –60° C, so that the sulfur dioxide could be removed from the system. [1,3,5-$C_6H_3(CNCH_3)_3$][($AsF_6)_3$] was obtained as a colorless solid, which decomposed at room temperature over some minutes.

Synthesis of [1,3,5-$C_6H_3(NH_3)_3$][($SbF_6)_3$]:

Antimony pentafluoride (430 mg, 1.98 mmol, 4.6 eq.) and anhydrous HF (ca. 3 mL) were condensed into an FEP reactor at –196 ° C. The mixture was homogenized at –40 ° C for 15 min. After freezing the solution, 1,3,5-Triaminobenzene trihydrochloride (100 mg,

0.43 mmol, 1 eq.) was added under nitrogen atmosphere. Followed by removing the nitrogen from the reaction vessel, the mixture was warmed to 0 ° C and vigorously mixed until all solid was dissolved. Cooling down to –20 ° C and –40 ° C led to formation of colorless crystals (see image 2, image 3), the mixture was then cooled down to –78° C, so that excess *a*HF could be removed from the system. [1,3,5-$C_6H_3(NH_3)_3$][($SbF_6)_3$] was obtained as a colorless solid, decomposition at room temperature over some hours was not observed. The crystalline compound was identified as [1,3,5-$C_6H_3(NH_3)_3$][($SbF_6)_3$] · HF.

Synthesis of [1,3,5-$C_6H_3(NH_3)_3$][($BF_4)_3$]:

Boron trifluoride (60.7 mg, 0.90 mmol, 4 eq.) and anhydrous HF (ca. 3 mL) were condensed into an FEP reactor at –196 ° C. The mixture was homogenized at –40 ° C for 15 min. The mixture was homogenized at –50 ° C for 15 min. After freezing the solution, 1,3,5-Triaminobenzene trihydrochloride (52.0 mg, 0.22 mmol, 1 eq.) was added under nitrogen atmosphere. Followed by removing the nitrogen from the reaction vessel, the mixture was warmed to 0 ° C and vigorously mixed until all solid was dissolved. The mixture was then cooled down to –78° C, so that excess *a*HF could be removed from the system. [1,3,5-$C_6H_3(NH_3)_3$][($BF_4)_3$] was obtained as a colorless solid, decomposition at room temperature over some hours was not observed.

2. Vibrational Data

Certain wavenumbers are underlined in the tables. They are assigned to the respective anions, although sometimes they may overlap with frequencies of the cation.

2.1 1,3,5-Tricyanobenzene

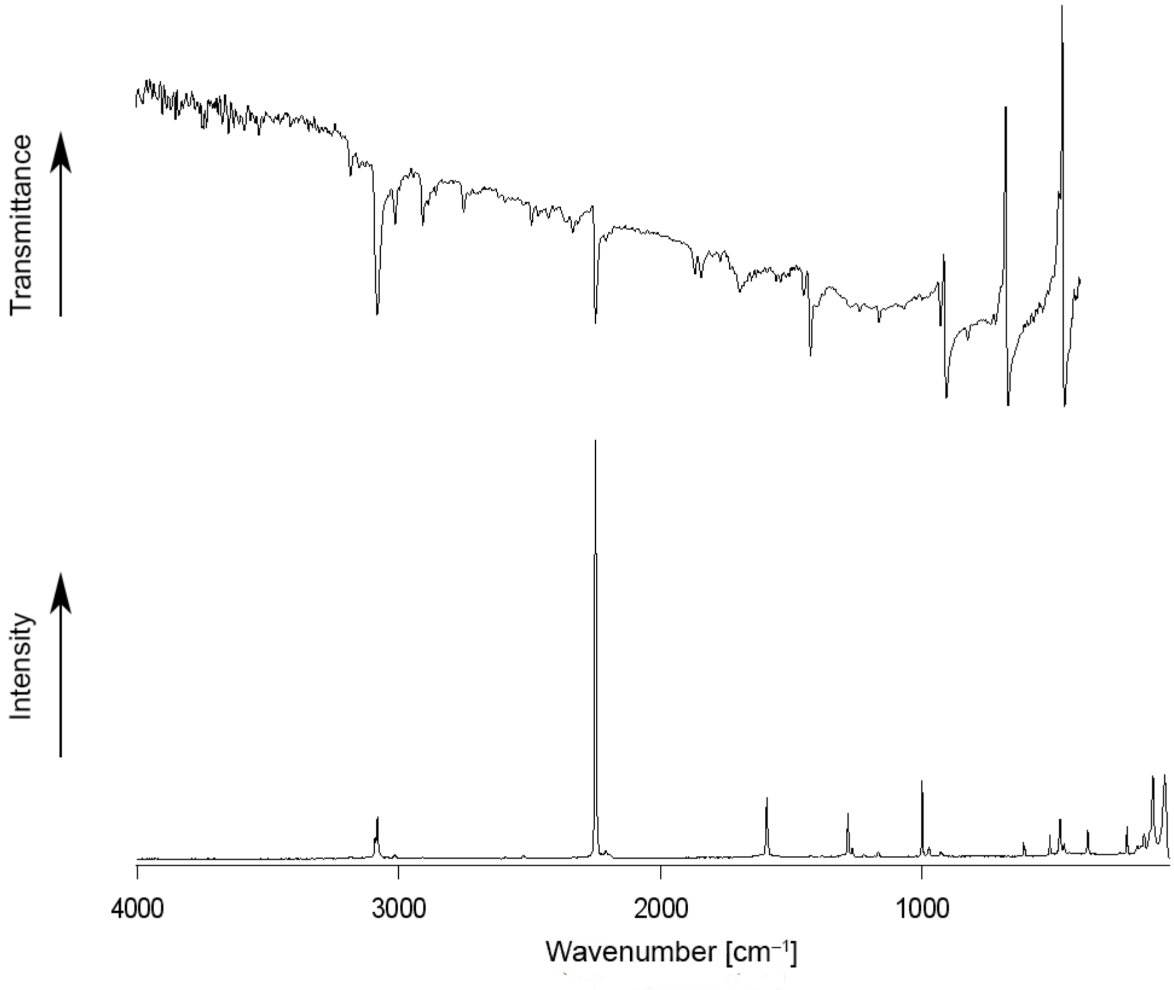

Figure 1. Raman and IR spectrum of 1,3,5-Tricyanobenzene.

Table 1. Observed vibrational frequencies and calculated vibrational frequencies of 1,3,5-tricyanobenzene [cm^{-1}] (calculated on B3LYP/6-311G++(3d2f, 3p2d) level of theory, IR intensities in km/mol, Raman intensities in $Å^4$/u).

exp. frequency		calc. frequency	
IR (Intensity)	Raman (Intensity)	(Intensity IR/Raman)	assignment
	3091(5)		
3082(m)	3081(10)	3233(4/169)	ν (CH)
3014(w)			
2908(w)			
2249(m)	2248(100)	2280(0/877)	ν (C≡N)
		2278(34/586)	ν (C≡N)
	2210(2)		
1869(w)			
1846(w)			
1699(w)			
	1594(15)	1621(14/160)	ν (CC) (ring)
1454(m)		1446(16/2)	δ (CH)
1429(w)			
	1285(11)	1316(0/107)	ν (CC) (**CC**N)
	1267(3)		
	1225(1)		
1165(w)	1170(2)	1182(2/16)	δ (CH)
	1001(19)		
	974(3)	1017(0/76)	ring breathing
932(m)	932(2)	951(16/4)	δ (ring)
908(s)		947(37/0)	ω (CH) (out of plane)
673(vs)		718(24/0)	δ (ring) (out of plane)
	614(4)	677(0/10)	δ (CCN) (out of plane)
	610(3)		
	513(6)	539(0/10)	δ (CN)

		492(3(0)	δ (CN) (out of plane)
457(vs)	474(10)	470(0/15)	δ (CN)
		469(0/15)	δ (CCN)
	459(4)	464(2/2)	δ (ring)
	367(7)	381(0/8)	δ (ring) (out of plane)
	220(8)		
	215(3)		
	179(3)		
	155(6)		
	120(20)	120(14/16)	δ (CCN)
	75(20)	98(16/0)	ω (CCN) (all)

Abbreviations for IR intensities: vs= very strong, s= strong, m= medium, w= weak; Raman intensities scaled to 100.

2.2 $[1,3,5\text{-}C_6H_3(CNH)_3][(MF_6)_3]$

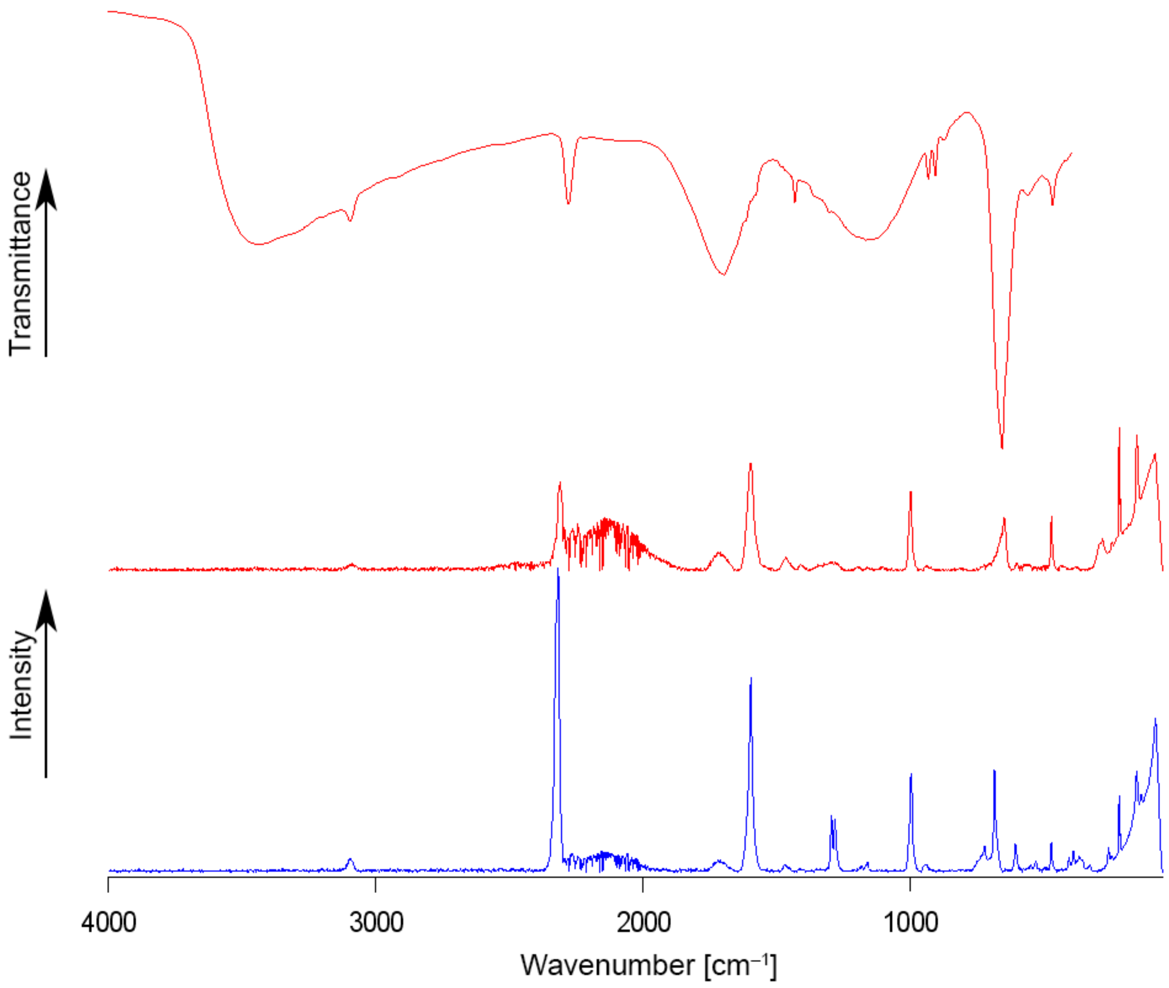

Figure 2. Raman (and IR spectra) of $[1,3,5\text{-}C_6H_3(CNH)_3][(SbF_6)_3]$ and $[1,3,5\text{-}C_6H_3(CNH)_3][(AsF_6)_3]$.

Table 2. Observed vibrational frequencies and calculated vibrational frequencies of $[1,3,5\text{-}C_6H_3(CNH)_3][(MF_6)_3]$ (M= As or Sb) [cm^{-1}] (calculated on B3LYP/6-311G++(3d2f, 3p2d) level of theory, IR intensities in km/mol, Raman intensities in $Å^4/u$).

exp. frequency $[1,3,5\text{-}C_6H_3(CNH)_3][(AsF_6)_3]$		exp. frequency $[1,3,5\text{-}C_6H_3(CNH)_3][(SbF_6)_3]$		calc. frequency $[1,3,5\text{-}C_6H_3(CNH)_3]$	
Raman (Intensity)		IR (Intensity	Raman (Intensity)	(Intensity IR/Raman)	assignment
				3575(0/35)	ν (NH)
		3437(m)		3564(41701 /0)	

3097(20)		3097(m)	3094(7)	3206(104/184)	ν (CH)
2327(69)			2312(70)	2331(0/767)	ν (C≡N)
		2280(m)		2326(292/638)	ν (C≡N)
		1701(m)	1713(22)		
1597(100)			1599(100)	1599(80/182)	ν (CC) (ring)
		1433(m)		1435(48/0)	ν (CC) (ring)
		1306(m)			
1293(39)				1277(0/102)	ν (CC) (**CC**N)
1280(46)		1169(m)			
				1184(96/13)	δ (CH)
999(65)			1000(72)	1003(0/70)	ring breathing
939(23)		933(m)		935(14/6)	δ (ring)
		908(m)			
		874(m)			
735(27)				728(394/2)	δ (NH)
724(28)					
701(34)		658(vs)		690(53/0)	δ (CCH)
685(49)			650(46)	677(0/14)	δ (CC) (out of plane)
675(39)					
607(30)				622(0/4)	τ (NH)
530(25)		563(m)		554(16/8)	δ (CCN)
473(41)			473(48)	457(0/17)	δ (CCN)
		469(m)		452(64/0)	δ (CC) (ring)
368(37)				387(0/8)	δ (out of plane)
			282(18)		
220(63)			220(100)		
215(39)					
154(61)			154(65)	123(12/18)	δ (CCN)

				105(27)	ω (CCNH)
100(55)					

Abbreviations for IR intensities: vs= very strong, s= strong, m= medium, w= weak; Raman intensities scaled to 100.

2.3 [1,3,5-$C_6H_3(CNCH_3)_3$][(MF_6)$_3$]

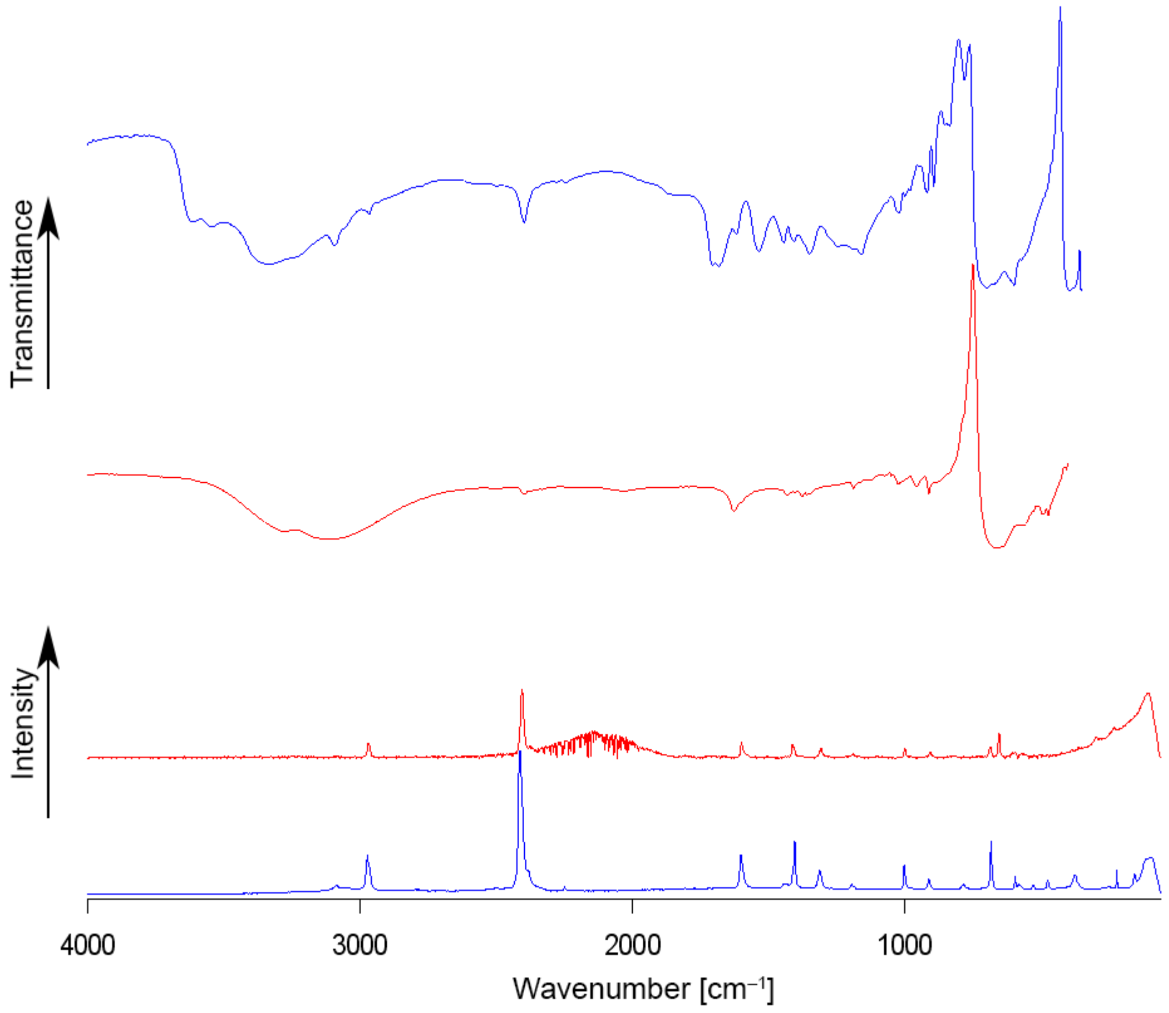

Figure 3. Raman and IR spectra of [1,3,5-$C_6H_3(CNCH_3)_3$][(SbF_6)$_3$] and [1,3,5-$C_6H_3(CNCH_3)_3$][(AsF_6)$_3$].

Table 3. Observed vibrational frequencies and calculated vibrational frequencies of [1,3,5-$C_6H_3(CNCH_3)_3$][$(MF_6)_3$] (M= As or Sb) [cm^{-1}] (calculated on B3LYP/6-311G++(3d2f, 3p2d) level of theory, IR intensities in km/mol, Raman intensities in $Å^4$/u).

exp. frequency [1,3,5-$C_6H_3(CNCH_3)_3$][$(AsF_6)_3$]		exp. frequency [1,3,5-$C_6H_3(CNCH_3)_3$][$(SbF_6)_3$]		calc. frequency [1,3,5-$C_6H_3(CNCH_3)_3$]	
IR (Intensity)	Raman (Intensity)	IR (Intensity)	Raman (Intensity)	(Intensity IR/Raman)	assignment
3612(m)					
3547(m)					
3340(m)		3275(s)		3218(62/121)	ν (CH)
3094(m)	3087(10)	3115(s)		3142(163/635)	ν (CH) (Me)
2966(m)	2973(32)		2969(25)	3046(367/1708)	ν (CH) (Me)
2397(m)	2412(100)	2397(m)	2405(100)	2409(0/2759)	ν (C≡N)
				2406(177/2129)	ν (C≡N)
1705(m)					
1682(m)					
1616(m)	1601(31)	1626(m)	1598(24)	1604(19/390)	ν (CC) (ring)
1533(m)					
1443(m)	1440(10)			1442(23/105)	δ (CH_3)
		1429(m)		1439(203/57)	δ (CH_3)
1412(m)				1432(0/265)	ω (CH_3)
1404(m)	1402(42)	1373(m)	1411(24)	1426(7/150)	ω (CH_3)
1348(m)		1360(m)			
	1311(19)		1305(17)	1307(0/143)	ν (CC) (**CC**N)
1246(m)				1200(49/41)	δ (CH)
1159(m)	1194(8)	1188(m)	1189(6)	1132(4/5)	ρ (CH_3)
				1129(2/4)	ρ (CH_3)
1018(m)		1022(m)		1012(2/10)	δ (CC) (**CC**N)
	999(22)		998(16)	1008(0/87)	ring breathing
		957(m)		958(22/0)	ω (CH)
916(w)	911(12)	910(m)	905(11)	847(0/137)	ν (C–N)
891(w)				746(0/64)	δ (ring) + ν (C–N)

852(w)					
833(w)					
779(vw)	783(7)				
696(s)	682(40)	669(vs)	684(19)	691(40/0)	ω (CH)
671(vs)		656(vs)	652(38)	663(0/22)	δ (ring) (out of plane)
596(s)	592(13)				
571(m)	576(7)	561(m)		562(4/13)	δ (CCN)
	526(6)			524(14/0)	δ (CCN) (out of plane)
		490(m)			
	474(7)	471(m)			
				421(0/12)	δ (CCN) (out of plane)
	374(14)			403(18/0)	δ (ring)
			296(11)	347(0/3)	δ ($CNCH_3$)
				270(26/2)	τ (NCH_3)
	220(13)		229(9)	245(10/1)	ω (NCH_3)
	154(12)				
	112(27)		107(14)	64(4/18)	ρ (NCH_3)
				61(10/0)	δ (NCH_3) (out of plane)

Abbreviations for IR intensities: vs= very strong, s= strong, m= medium, w= weak, vw= very weak; Raman intensities scaled to 100.

2.3 [1,3,5-$C_6H_3(NH_3)_3$]

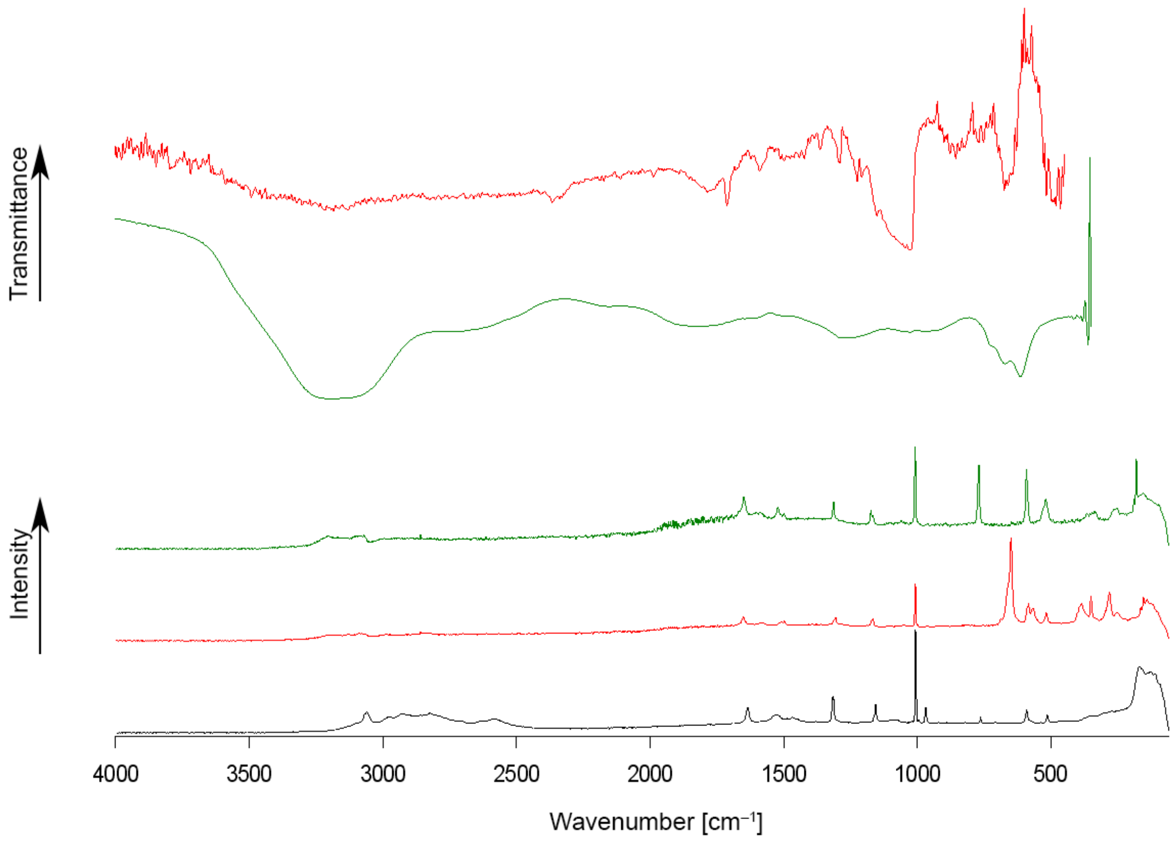

Figure 4. Raman (and IR spectra) of [1,3,5-$C_6H_3(NH_3)_3$][Cl_3], [1,3,5-$C_6H_3(NH_3)_3$][$(SbF_6)_3$] and [1,3,5-$C_6H_3(CNH)_3$][$(BF_4)_3$].

Table 4. Observed vibrational frequencies and calculated vibrational frequencies of [1,3,5-$C_6H_3(NH_3)_3$][Cl_3], [1,3,5-$C_6H_3(NH_3)_3$][$(SbF_6)_3$] and [1,3,5-$C_6H_3(NH_3)_3$][$(BF_4)_3$] [cm^{-1}] (calculated on B3LYP/6-311G++(3d2f, 3p2d) level of theory, IR intensities in km/mol, Raman intensities in Å^4/u).

[1,3,5-$C_6H_3(NH_3)_3$][Cl_3]	[1,3,5-$C_6H_3(NH_3)_3$][$(SbF_6)_3$]		[1,3,5-$C_6H_3(NH_3)_3$][$(BF_4)_3$]		calc. frequency [1,3,5-$C_6H_3(NH_3)_3$]	
Raman (Intensity)	IR (Intensity) SbF_6^-	Raman (Intensity) SbF_6^-	IR (Intensity) BF_4^-	Raman (Intensity) BF_4^-	(Intensity IR/Raman)	assignment
					3414(397/101)	ν (NH_3)
					3389(514/94)	ν (NH_3)

	3219(m) br	3195(6)	3195(vs) br	3211(13)	3340(0/319)	ν (NH_3)
		3129(7)		3166(12)	3335(576/38)	ν (NH_3)
3059(21)		3079(7)		3092(14)	3190(52/178)	ν (CH)
2981(16)		2987(7)				
2928(19)		2829(8)				
2822(19)			2744(m) sh			
2585(14)						
	2366(m)					
	1786(m)		1848(m)			
	1713(m)					
					1666(58/28)	ν (CC) + δ (NH_3)
1635(25)		1651(23)	1622(m)	1649(51)	1649(0/3)	δ (NH_3)
					1645(191/18)	δ (NH_3)
					1629(124/2)	ν (CC) + δ (NH_3)
					1579(0(10)	ω (NH_3)
1525(18)	1591(m)	1580(18)		1522(41)	1574(572/14)	ω (NH_3)
		1509(19)			1505(50/1)	ν (CC) + δ (CH)
1483(14)		1498(20)	1495(m)			
1468(16)						
1317(36)	1294(w)	1305(23)	1286(m)	1313(46)		
	1225(w)		1252(m)			
	1207(w)			1174(38)	1261(0/5)	ν (CN)
1156(28)	1151(s)	1166(21)		1167(34)	1177(0/6)	δ (CH)
1101(13)	1059(s)				1114(73/0)	τ (NH_3)
1080(13)	1045(s)				1064(0/3)	ρ (NH_3)
	1028(vs)		1028(m)		1061(58/0)	ρ (NH_3)
1007(100)		1008(56)		1008(100)	1021(0/27)	ring breathing
995(13)						
968(25)						
					889(14/0)	ω (CH)

891(11)						
764(15)				769(82)		
	675(m)	688(21)	671(m)		674(42/0)	δ (CC) (out of plane)
		650(100)				
			613(s)			
591(22)		584(37)		592(78)		
538(11)		566(32)			548(0/21)	δ (ring + CN)
					546(0/1)	δ (HCCN) (out of plane)
514(18)	517(m)	518(27)		519(49)	518(15/8)	δ (ring)
	465(m)					
		387(26)		366(35)		
351(16)		350(44)		336(38)		
304(20)		281(47)		266(39)	291(26/2)	δ (CN)
		252(27)		253(40)	237(0/2)	δ (out of plane)
169(65)		164(32)		181(88)	206(83/0)	δ (CN) (out of plane)
		154(42)		151(54)		
128(60)		140(40)				
110(58)						

Abbreviations for IR intensities: vs= very strong, s= strong, m= medium, w= weak, br= broad, sh= shoulder; Raman intensities scaled to 100.

3. Crystal Structures

3.1 [1,3,5-$C_6H_3(CNH)_3$][(SbF_6)(Sb_2F_{11})$_2$] · 3HF

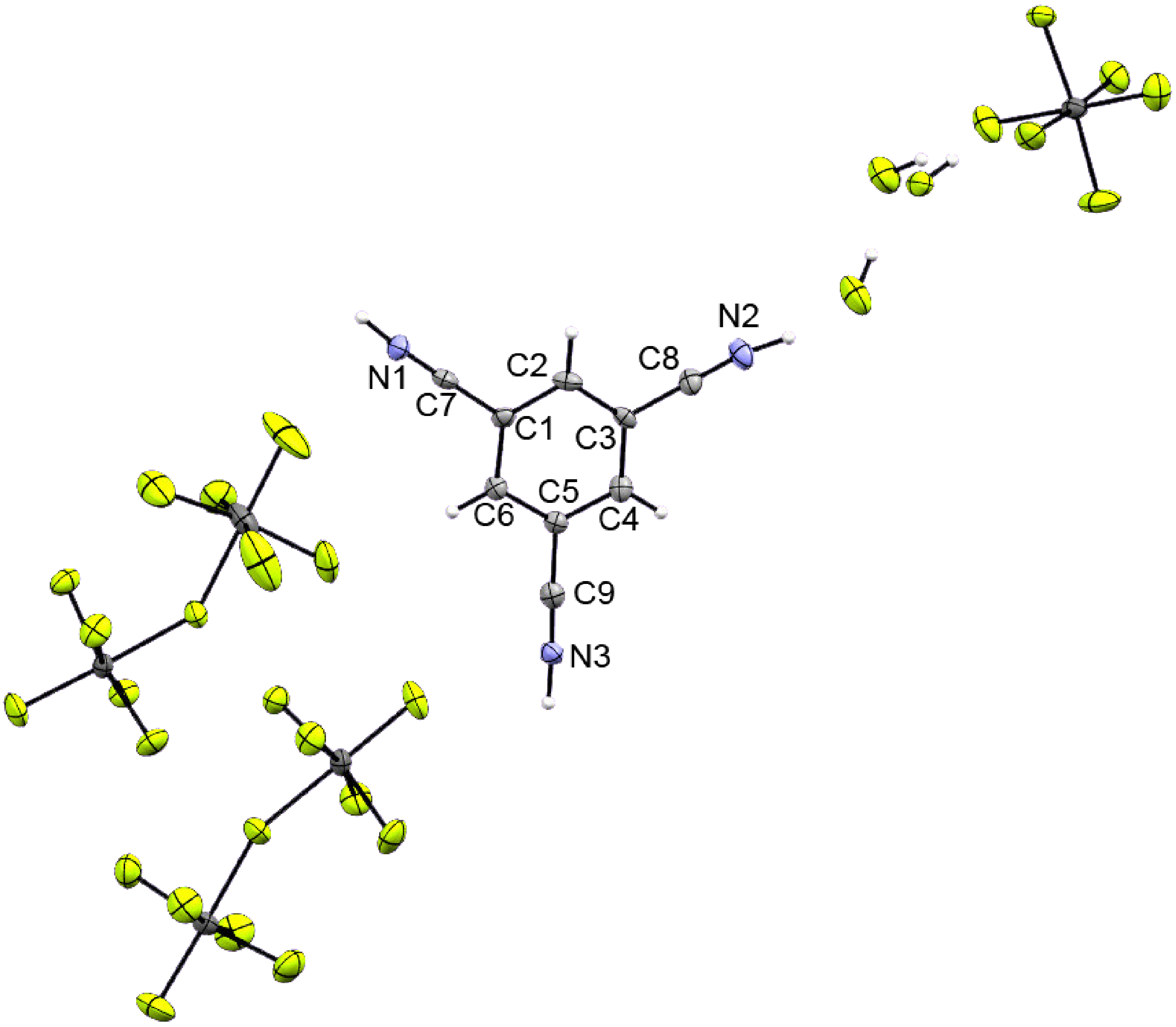

Figure 5. Asymmetric unit of [1,3,5-$C_6H_3(CNH)_3$][(SbF_6)(Sb_2F_{11})$_2$] · 3HF, view along *a*, displacement ellipsoids at 50% probability.

[1,3,5-$C_6H_3(CNH)_3$][(SbF_6)(Sb_2F_{11})$_2$] · HF crystallizes in the triclinic space group *P*–1. A unit cell contains 2 formula units.

Table 5. Selected bond lengths (Å) of [1,3,5-$C_6H_3(CNH)_3$][(SbF_6)(Sb_2F_{11})$_2$] · 3HF.

N2	C8	1.125(7)
N1	C7	1.135(7)
N3	C9	1.141(7)
C7	C1	1.426(7)

C3	C2	1.382(7)
C3	C4	1.387(7)
C3	C8	1.441(7)
C5	C4	1.398(7)
C5	C6	1.399(7)
C5	C9	1.425(7)
C1	C6	1.390(7)
C1	C2	1.398(7)

Table 6. Selected bond angles (°) of $[1,3,5\text{-}C_6H_3(CNH)_3][(SbF_6)(Sb_2F_{11})_2]\cdot 3HF$.

N1	C7	C1	179.1(6)
C2	C3	C4	122.5(5)
C2	C3	C8	120.1(4)
C4	C3	C8	117.3(5)
C4	C5	C6	122.4(5)
C4	C5	C9	117.6(5)
C6	C5	C9	120.0(4)
N2	C8	C3	176.9(6)
C6	C1	C2	122.6(5)
C6	C1	C7	120.0(5)
C2	C1	C7	117.4(4)
N3	C9	C5	177.9(5)
C3	C2	C1	117.8(4)
C3	C4	C5	117.6(5)
C1	C6	C5	117.0(4)

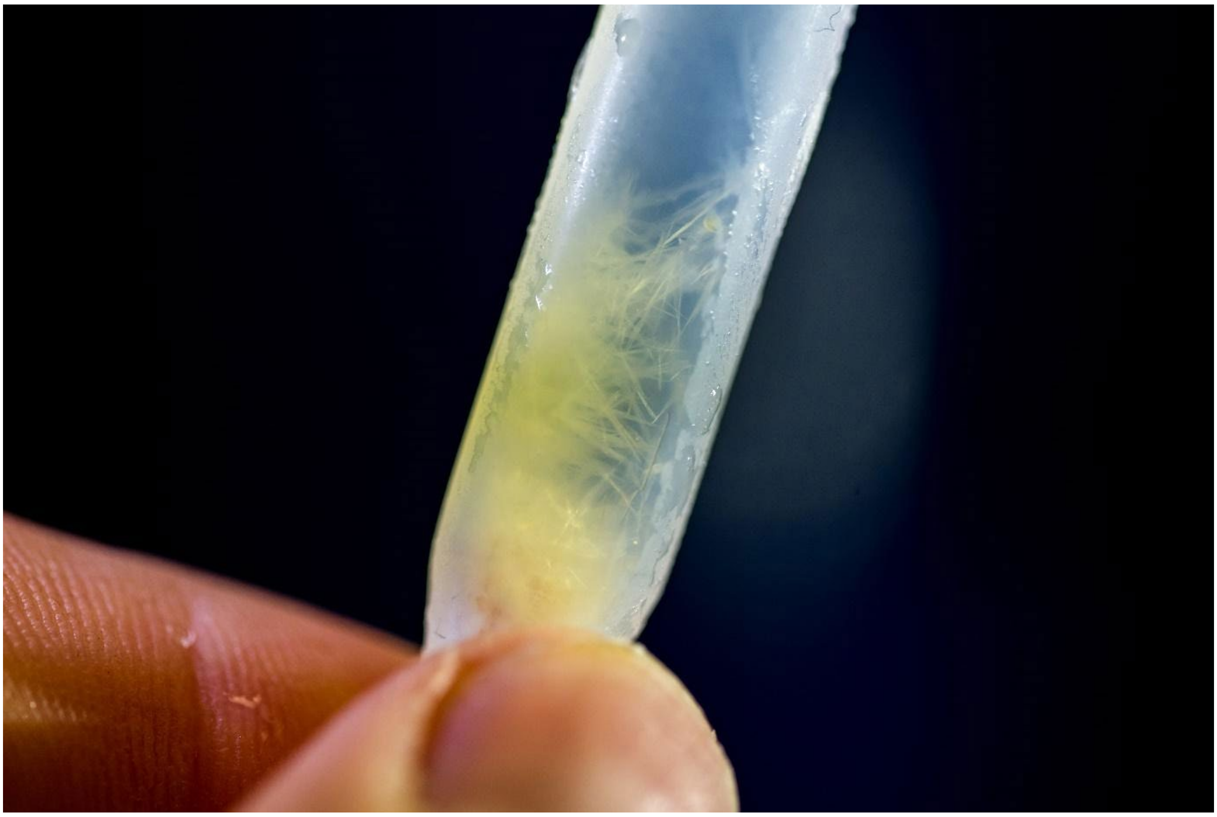

Image 1. Crystals of [1,3,5-$C_6H_3(CNH)_3$][(SbF_6)(Sb_2F_{11})$_2$] · 3HF.

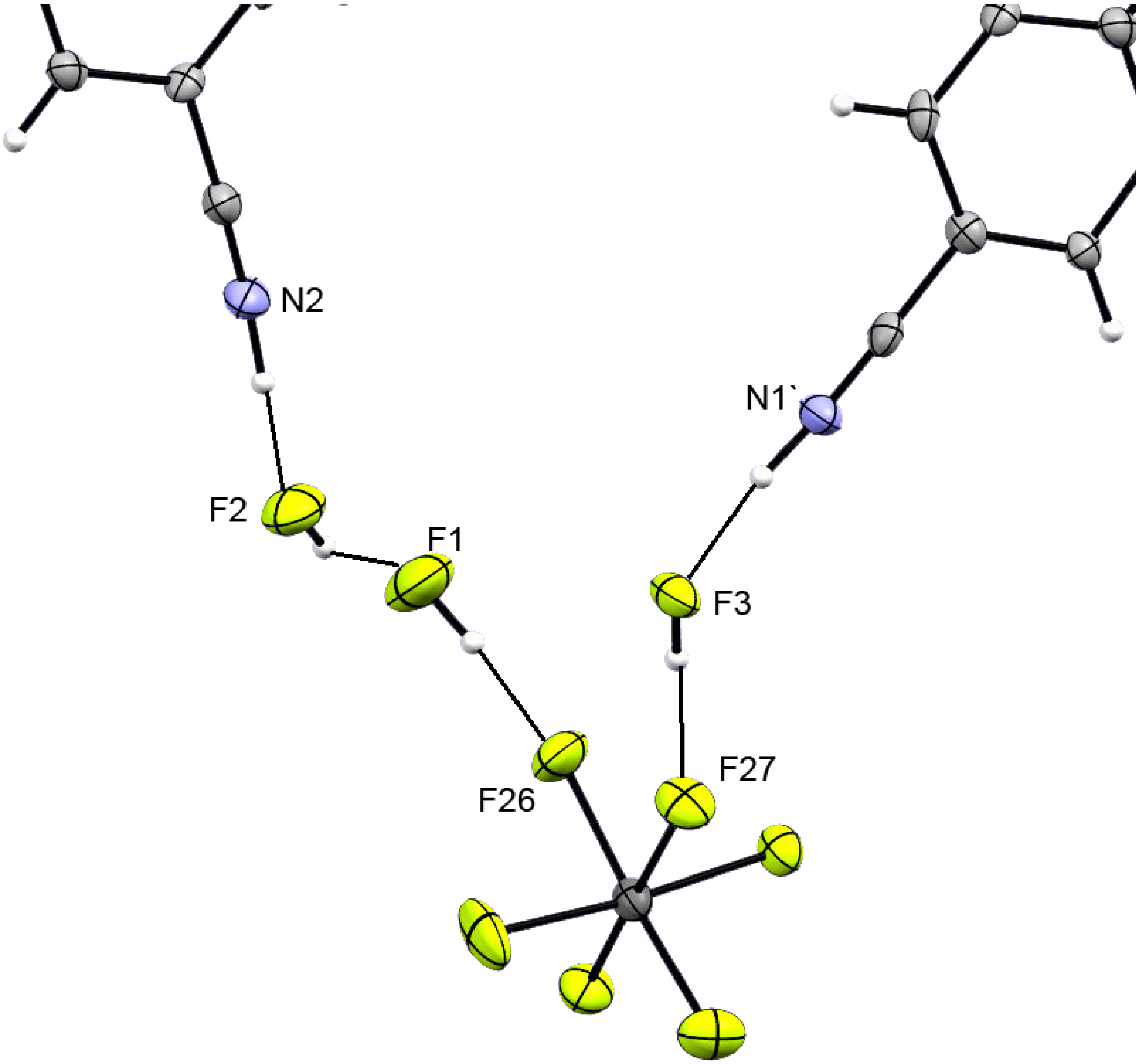

Figure 6. Particular hydrogen bonds in $[1,3,5\text{-}C_6H_3(CNH)_3][(SbF_6)(Sb_2F_{11})_2] \cdot 3HF$, view along *a*, displacement ellipsoids at 50% probability.

Table 7. Particular H-bond lengths (Å) for $[1,3,5\text{-}C_6H_3(CNH)_3][(SbF_6)(Sb_2F_{11})_2] \cdot 3HF$.

N2 (via H2N)	F2	2.516(6)
F2 (via H2F)	F1	2.462(6)
F1 (via H1F)	F26	2.499(5)
N1` (via H1N`)	F3	2.540(7)
F3 (via H3F)	F27	2.501(5)

All hydrogens, even the CH aromatic ones, do exhibit hydrogen bonding of various strength with the anions, furthermore, all carbon atoms show weaker interactions with the fluorines of the anions. Noteworthy is the hydrogen-bonding via linkage of co-crystallized hydrogen fluorides from the cation(s) to the single SbF_6^-.

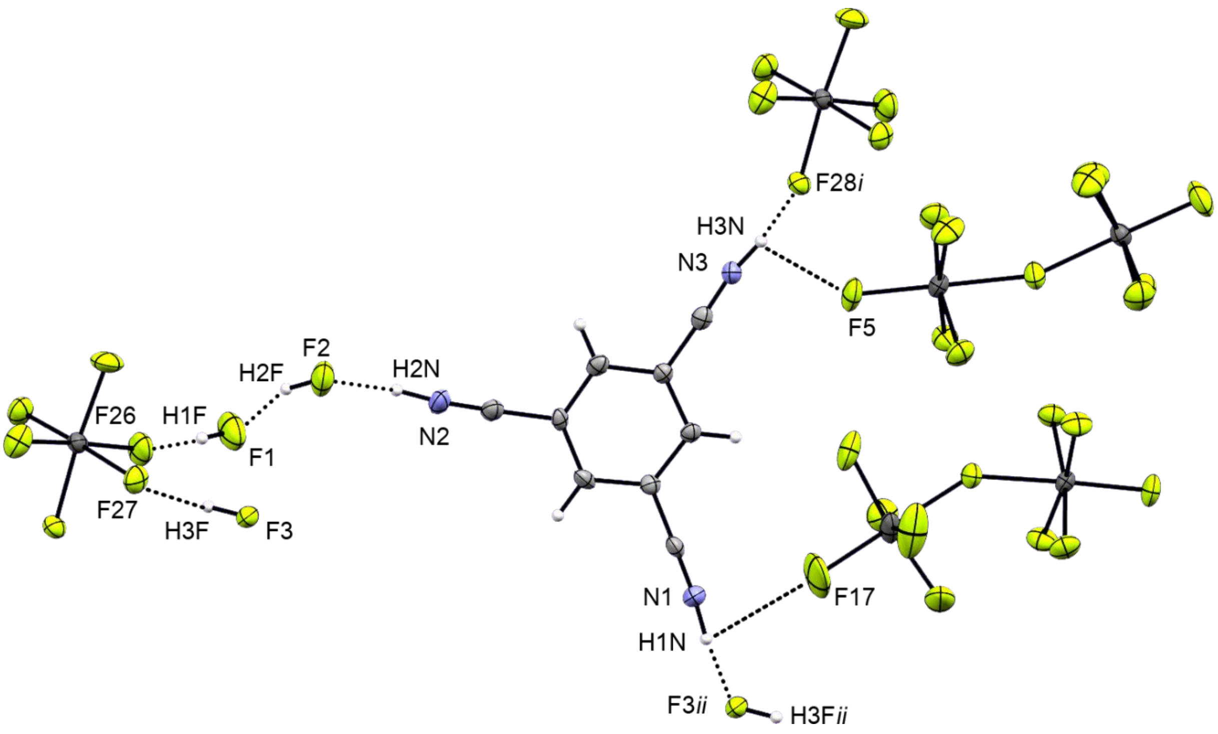

Figure 7. Selected hydrogen bonds of **[1+3H]**$[(Sb_2F_{11})_2(SbF_6)] \cdot 3HF$, view along *a*, displacement ellipsoids at 50% probability. *i* = x, –1+y, 1+z, *ii* = 1–x, 1–y, –z.

Table 8. Selected hydrogen bond lengths of **[1+3H]**$[(Sb_2F_{11})_2(SbF_6)] \cdot 3HF$ in Å.

Bond	Length	Bond	Length
N2(H2N)···F2	2.516(6)	N1(H1N)···F3ii	2.540(7)
F2(H2F)···F1	2.462(6)	N3(H3N)···F28i	2.546(5)
F1(H1F)···F26	2.499(5)	H3N···F5	2.53(5)
F3(H3F)···F27	2.501(5)	H1N···F17	2.75(4)

Table 9. Data collection and structure refinement for $[1,3,5\text{-}C_6H_3(CNH)_3][(SbF_6)(Sb_2F_{11})_2] \cdot 3HF$.

	$[1,3,5\text{-}C_6H_3(CNH)_3][(SbF_6)(Sb_2F_{11})_2] \cdot 3HF$
**Chemical formula**	$C_9\ H_9\ F_{31}\ N_6\ Sb_5$
**Formula weight**	1356.99 g/mol
**Temperature**	113(2) K
**Wavelength**	0.71073 Å
**Crystal size**	0.200 x 0.110 x 0.040 mm
**Crystal habit**	colorless plate

**Crystal system**	triclinic	
**Space group**	P –1	
**Unit cell dimensions**	a = 8.2746(3) Å	α = 75.109(4)°
	b = 12.8517(5) Å	β = 75.109(4)°
	c = 15.3257(7) Å	γ = 83.092(3)°
**Volume**	1542.72(11) $Å^3$	
**Z**	2	
**Density (calculated)**	2.921 g/cm^3	
**Absorption coefficient**	4.520 mm^{-1}	
**F(000)**	1236.0	
**Diffractometer**	Oxford XCalibur	
**Radiation source**	MoKα , λ = 0.71073 Å	
**Index ranges**	-10<=h<=11, -17<=k<=17, -20<=l<=18	
**Reflections collected**	7643	
**Absorption correction**	multi-scan	
**Max. and min. transmission**	1.000 and 0.659	
**Structure solution program**	SHELXT 2018/2 (Sheldrick, 2018)	
**Refinement method**	Full-matrix least-squares on F^2	
**Refinement program**	SHELXL-2018/3 (Sheldrick, 2018)	
**Goodness-of-fit on F^2**	1.028	
**Final R indices**	6127 data; I>2σ(I)	R1 = 0.0372, wR2 = 0.0921
	all data	R1 = 0.0529
**Weighting scheme**	w=1/[¥s^2^(Fo^2^)+(0.0442P)^2^] where P=(Fo^2^+2Fc^2^)/3	
**Largest diff. peak and hole**	2.945 and -1.613 $eÅ^{-3}$	
**R.M.S. deviation from mean**	0.189 $eÅ^{-3}$	
**CCDC-deposition number**	2085061	

Table 10. Bond lengths (Å) of $[1,3,5\text{-}C_6H_3(CNH)_3][(SbF_6)(Sb_2F_{11})_2]$ · 3HF.

Sb4	F23	1.847(3)
Sb4	F22	1.852(3)
Sb4	F21	1.855(3)
Sb4	F24	1.856(3)
Sb4	F25	1.860(3)
Sb4	F20	2.019(3)

Sb1	F5	1.841(3)
Sb1	F8	1.853(3)
Sb1	F6	1.860(3)
Sb1	F4	1.862(3)
Sb1	F7	1.865(3)
Sb1	F9	2.051(3)
Sb2	F14	1.841(3)
Sb2	F12	1.848(3)
Sb2	F13	1.853(3)
Sb2	F10	1.854(3)
Sb2	F11	1.862(4)
Sb2	F9	2.008(3)
Sb5	F30	1.847(3)
Sb5	F29	1.856(3)
Sb5	F31	1.857(3)
Sb5	F26	1.885(3)
Sb5	F28	1.890(3)
Sb5	F27	1.894(3)
Sb3	F15	1.839(3)
Sb3	F17	1.850(4)
Sb3	F18	1.853(4)
Sb3	F16	1.862(4)
Sb3	F19	1.863(4)
Sb3	F20	2.036(3)
N2	C8	1.125(7)
N1	C7	1.135(7)
N3	C9	1.141(7)
C7	C1	1.426(7)
C3	C2	1.382(7)
C3	C4	1.387(7)
C3	C8	1.441(7)
C5	C4	1.398(7)
C5	C6	1.399(7)
C5	C9	1.425(7)
C1	C6	1.390(7)
C1	C2	1.398(7)

Table 11. Bond angles (°) for [1,3,5-$C_6H_3(CNH)_3$][(SbF_6)(Sb_2F_{11})$_2$] · 3HF.

F23	Sb4	F22	95.85(16)
F23	Sb4	F21	94.65(16)
F22	Sb4	F21	89.98(14)
F23	Sb4	F24	93.40(15)
F22	Sb4	F24	90.06(14)
F21	Sb4	F24	171.91(15)
F23	Sb4	F25	93.43(16)
F22	Sb4	F25	170.71(14)
F21	Sb4	F25	89.23(14)
F24	Sb4	F25	89.43(14)
F23	Sb4	F20	177.99(15)
F22	Sb4	F20	85.39(14)
F21	Sb4	F20	86.92(14)
F24	Sb4	F20	85.01(14)
F25	Sb4	F20	85.33(14)
F5	Sb1	F8	94.92(15)
F5	Sb1	F6	95.56(16)
F8	Sb1	F6	169.51(15)
F5	Sb1	F4	96.36(15)
F8	Sb1	F4	90.23(15)
F6	Sb1	F4	88.84(16)
F5	Sb1	F7	95.11(15)
F8	Sb1	F7	88.79(15)
F6	Sb1	F7	90.05(16)
F4	Sb1	F7	168.53(15)
F5	Sb1	F9	178.76(16)
F8	Sb1	F9	84.22(14)
F6	Sb1	F9	85.29(15)
F4	Sb1	F9	84.54(15)
F7	Sb1	F9	83.99(15)

F14	Sb2	F12	94.05(18)
F14	Sb2	F13	91.45(16)
F12	Sb2	F13	93.21(17)
F14	Sb2	F10	89.54(17)
F12	Sb2	F10	93.60(18)
F13	Sb2	F10	173.03(16<br>)
F14	Sb2	F11	172.47(17<br>)
F12	Sb2	F11	93.34(19)
F13	Sb2	F11	89.55(17)
F10	Sb2	F11	88.58(17)
F14	Sb2	F9	85.91(16)
F12	Sb2	F9	178.54(16<br>)
F13	Sb2	F9	85.33(15)
F10	Sb2	F9	87.87(16)
F11	Sb2	F9	86.73(17)
F30	Sb5	F29	92.71(15)
F30	Sb5	F31	92.00(17)
F29	Sb5	F31	92.54(18)
F30	Sb5	F26	90.75(16)
F29	Sb5	F26	176.11(17<br>)
F31	Sb5	F26	89.11(18)
F30	Sb5	F28	90.94(14)
F29	Sb5	F28	90.96(15)
F31	Sb5	F28	175.31(15<br>)
F26	Sb5	F28	87.20(15)
F30	Sb5	F27	177.06(15<br>)
F29	Sb5	F27	88.37(15)
F31	Sb5	F27	90.68(16)
F26	Sb5	F27	88.09(16)
F28	Sb5	F27	86.31(14)
F15	Sb3	F17	96.0(2)

F15	Sb3	F18	168.54(19<br>)
F17	Sb3	F18	95.5(2)
F15	Sb3	F16	89.95(19)
F17	Sb3	F16	92.5(2)
F18	Sb3	F16	89.7(2)
F15	Sb3	F19	89.87(17)
F17	Sb3	F19	93.9(2)
F18	Sb3	F19	89.2(2)
F16	Sb3	F19	173.50(17<br>)
F15	Sb3	F20	84.83(15)
F17	Sb3	F20	179.06(19<br>)
F18	Sb3	F20	83.71(17)
F16	Sb3	F20	87.03(15)
F19	Sb3	F20	86.48(15)
Sb4	F20	Sb3	143.64(17<br>)
Sb2	F9	Sb1	158.5(2)
N1	C7	C1	179.1(6)
C2	C3	C4	122.5(5)
C2	C3	C8	120.1(4)
C4	C3	C8	117.3(5)
C4	C5	C6	122.4(5)
C4	C5	C9	117.6(5)
C6	C5	C9	120.0(4)
N2	C8	C3	176.9(6)
C6	C1	C2	122.6(5)
C6	C1	C7	120.0(5)
C2	C1	C7	117.4(4)
N3	C9	C5	177.9(5)
C3	C2	C1	117.8(4)
C3	C4	C5	117.6(5)
C1	C6	C5	117.0(4)

Table 12. Anisotropic atomic displacement parameters ($Å^2$) for [1,3,5-$C_6H_3(CNH)_3$][(SbF_6)(Sb_2F_{11})$_2$] · 3HF.

	**U_{11}**	**U_{22}**	**U_{33}**	**U_{23}**	**U_{13}**	**U_{12}**
Sb4	0.01948(17)	0.01657(16)	0.02365(18)	-0.00588(13)	-0.00203(13)	-0.00288(12)
Sb1	0.02233(18)	0.01735(17)	0.02776(19)	-0.00574(13)	-0.00301(14)	-0.00263(13)
Sb2	0.02278(18)	0.02575(19)	0.02086(18)	-0.00139(13)	-0.00209(13)	-0.00311(14)
Sb5	0.02504(18)	0.02521(18)	0.01793(17)	-0.00339(13)	-0.00216(13)	-0.00793(14)
Sb3	0.0285(2)	0.0264(2)	0.0455(3)	0.00942(17)	-0.01606(17)	-0.00764(16)
F20	0.0402(19)	0.0191(15)	0.0297(17)	-0.0032(12)	-0.0097(14)	-0.0007(13)
F28	0.0341(18)	0.0334(17)	0.0181(16)	-0.0036(13)	-0.0008(13)	-0.0109(14)
F22	0.0217(15)	0.0358(18)	0.0303(17)	-0.0097(14)	-0.0082(13)	-0.0016(13)
F24	0.0317(18)	0.0385(19)	0.0285(17)	-0.0157(14)	-0.0057(14)	0.0077(15)
F8	0.0345(18)	0.0290(17)	0.0292(18)	-0.0101(13)	-0.0028(14)	0.0055(14)
F21	0.0271(17)	0.045(2)	0.0289(18)	-0.0187(15)	0.0028(13)	-0.0019(15)
F7	0.0302(18)	0.0378(19)	0.044(2)	-0.0086(15)	-0.0146(15)	-0.0037(15)
F25	0.0208(16)	0.0414(19)	0.043(2)	-0.0167(15)	-0.0090(14)	-0.0057(14)
F30	0.0310(18)	0.0337(18)	0.042(2)	-0.0047(15)	-0.0070(15)	-0.0126(14)
F3	0.052(2)	0.0285(17)	0.0238(17)	-0.0062(13)	-0.0051(15)	-0.0174(15)
F27	0.0342(19)	0.0369(18)	0.0317(18)	-0.0056(14)	0.0055(14)	-0.0179(15)
F4	0.0280(17)	0.0370(19)	0.043(2)	-0.0116(15)	-0.0050(14)	-0.0122(14)
F5	0.038(2)	0.0183(16)	0.055(2)	0.0029(15)	-0.0129(16)	-0.0034(14)
F29	0.0282(18)	0.0327(18)	0.055(2)	-0.0133(16)	0.0018(16)	-0.0024(15)
F9	0.066(3)	0.0258(17)	0.0267(18)	0.0004(14)	-0.0054(17)	-0.0107(17)
F13	0.043(2)	0.0283(18)	0.046(2)	-0.0111(15)	-0.0015(16)	0.0016(15)
F26	0.048(2)	0.0279(18)	0.053(2)	0.0024(16)	-0.0209(18)	0.0033(16)
F11	0.0296(19)	0.063(3)	0.052(2)	-0.0181(19)	-0.0093(17)	-0.0092(18)
F14	0.0266(18)	0.046(2)	0.049(2)	-0.0099(17)	-0.0069(16)	-0.0094(15)
F6	0.038(2)	0.041(2)	0.046(2)	-0.0270(17)	0.0068(16)	-0.0018(16)
F19	0.047(2)	0.0209(17)	0.068(3)	-0.0055(16)	0.0042(19)	-0.0087(16)
F31	0.070(3)	0.070(3)	0.0204(18)	-0.0120(17)	-0.0059(17)	-0.034(2)
F12	0.074(3)	0.048(2)	0.030(2)	0.0121(17)	-0.0085(19)	-0.005(2)
F23	0.062(3)	0.0177(16)	0.045(2)	0.0020(14)	-0.0101(18)	-0.0100(16)
F2	0.053(3)	0.0286(19)	0.059(3)	0.0053(17)	-0.016(2)	-0.0109(17)

F15	0.034(2)	0.051(2)	0.046(2)	-0.0124(18)	0.0017(16)	-0.0072(17)
F10	0.046(2)	0.047(2)	0.042(2)	-0.0232(17)	0.0063(17)	-0.0017(18)
F16	0.085(3)	0.050(2)	0.053(3)	0.0005(19)	-0.041(2)	-0.025(2)
F1	0.081(3)	0.035(2)	0.050(3)	0.0006(18)	-0.013(2)	0.011(2)
N2	0.032(3)	0.024(2)	0.036(3)	-0.002(2)	-0.012(2)	-0.010(2)
F18	0.022(2)	0.046(2)	0.137(5)	0.014(3)	-0.013(2)	-0.0028(18)
N1	0.035(3)	0.024(2)	0.025(3)	-0.0067(19)	-0.007(2)	-0.008(2)
N3	0.027(2)	0.021(2)	0.021(2)	-0.0022(17)	-0.0038(18)	-0.0057(18)
C7	0.023(3)	0.023(3)	0.015(2)	0.0024(19)	-0.0045(19)	-0.003(2)
F17	0.100(4)	0.062(3)	0.084(4)	0.041(3)	-0.057(3)	-0.021(3)
C3	0.013(2)	0.019(2)	0.019(2)	0.0017(18)	-0.0012(18)	-0.0058(18)
C5	0.014(2)	0.021(2)	0.019(2)	-0.0035(19)	-0.0022(18)	-0.0029(19)
C8	0.025(3)	0.023(3)	0.028(3)	-0.006(2)	-0.010(2)	-0.003(2)
C1	0.018(2)	0.023(2)	0.017(2)	-0.0059(19)	0.0010(18)	-0.0043(19)
C9	0.016(2)	0.022(3)	0.027(3)	-0.005(2)	-0.002(2)	-0.0024(19)
C2	0.017(2)	0.032(3)	0.014(2)	0.000(2)	-0.0035(18)	-0.007(2)
C4	0.017(2)	0.021(2)	0.029(3)	-0.007(2)	-0.007(2)	-0.0018(19)
C6	0.016(2)	0.020(2)	0.022(3)	-0.0045(19)	-0.0048(19)	-0.0033(19)

3.2 $[1,3,5\text{-}C_6H_3(NH_3)_3][(SbF_6)_3] \cdot HF$

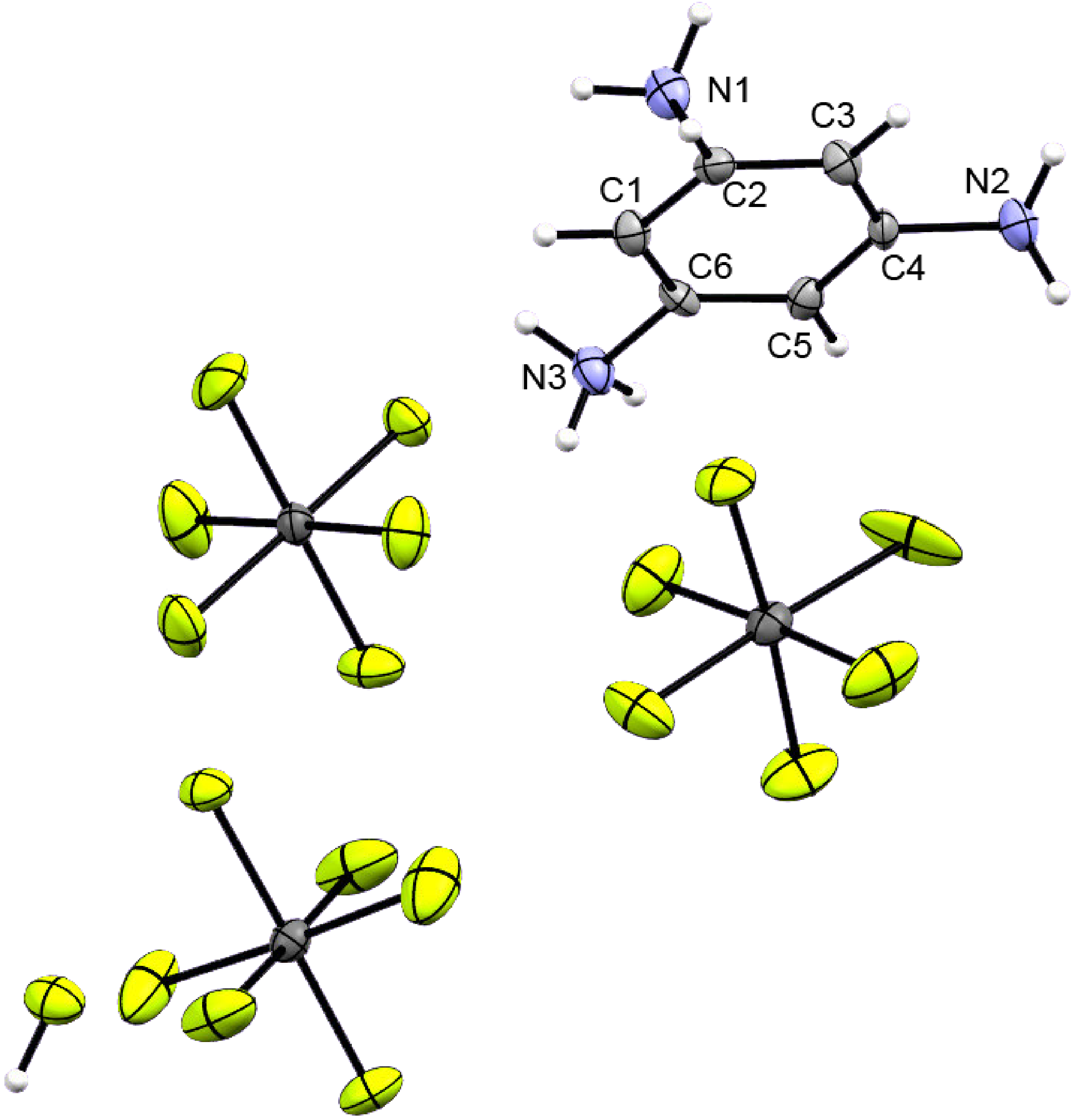

Figure 8. Asymmetric unit of $[1,3,5\text{-}C_6H_3(NH_3)_3][(SbF_6)_3] \cdot HF$, view along *b*, displacement ellipsoids at 50% probability.

$[1,3,5\text{-}C_6H_3(NH_3)_3][(SbF_6)_3] \cdot HF$ crystallizes in the monoclinic space group $P2_1/n$. A unit cell contains 4 formula units.

Hydrogen bonds between the anion and cation are present, the strongest hydrogen bond is 2.702(5) Å long, located between a fluorine of an anion and a NH_3^+ moiety, most are longer than 2.8 Å.

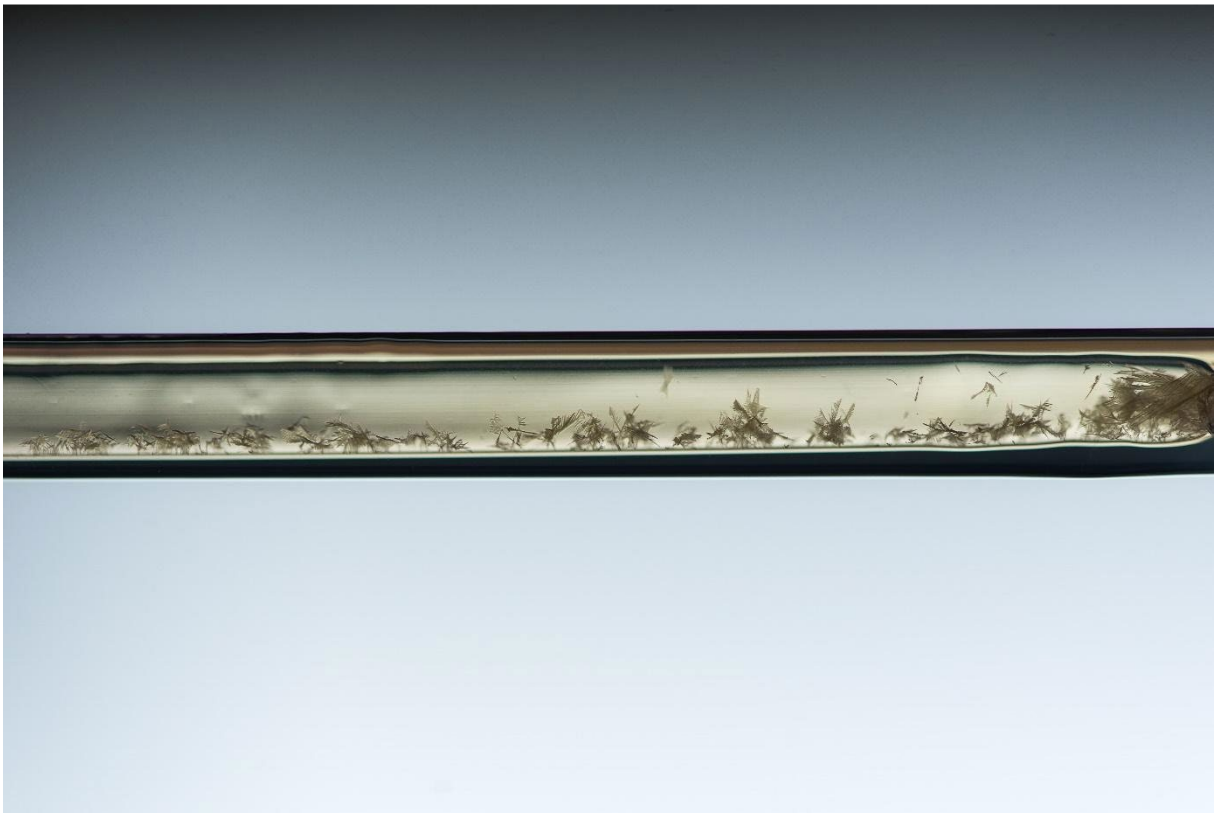

Image 2. Crystals of $[1,3,5\text{-}C_6H_3(NH_3)_3][(SbF_6)_3] \cdot HF$ inside of a NMR tube.

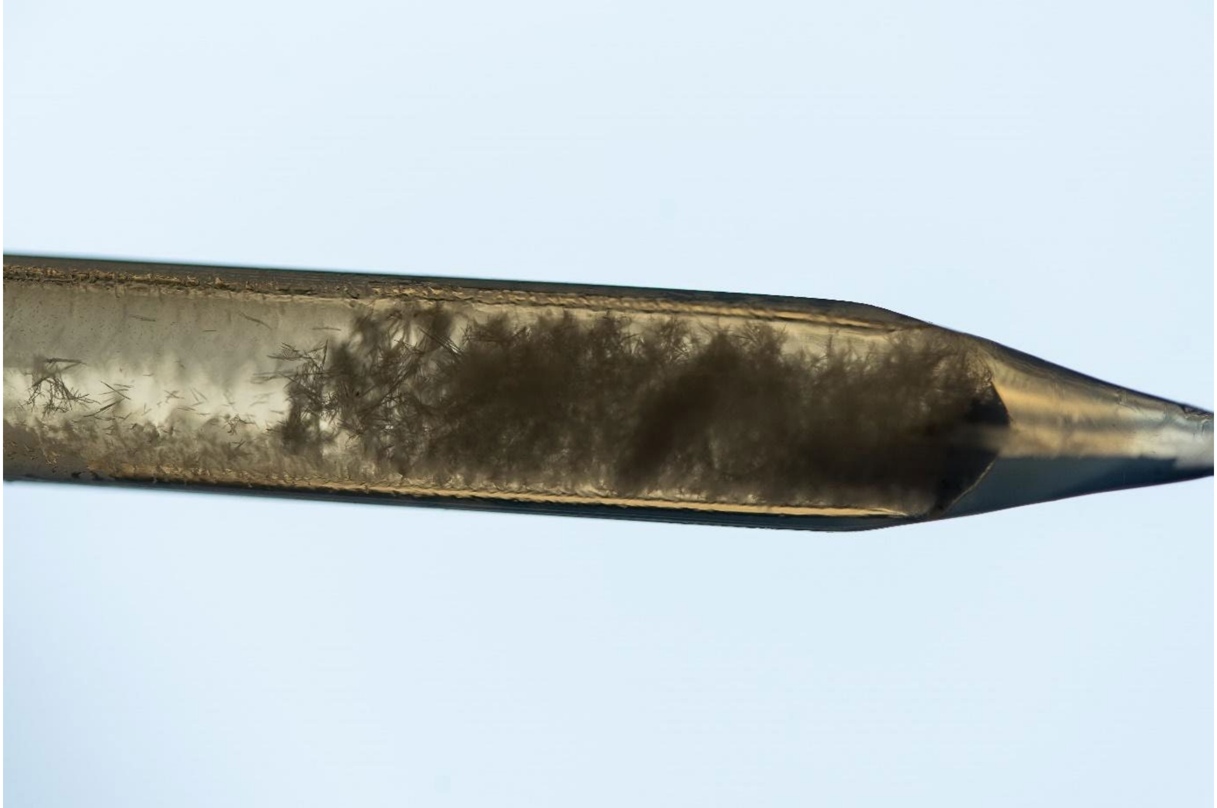

Image 3. Crystals of $[1,3,5\text{-}C_6H_3(NH_3)_3][(SbF_6)_3] \cdot HF$ inside a reactor.

Table 13. Selected bond lengths (Å) of $[1,3,5\text{-}C_6H_3(NH_3)_3][(SbF_6)_3]\cdot HF$.

N1	C2	1.460(7)
N3	C6	1.480(7)
N2	C4	1.484(7)
C4	C5	1.368(8)
C4	C3	1.372(7)
C5	C6	1.383(8)
C6	C1	1.374(8)
C2	C1	1.375(8)
C2	C3	1.385(8)

Table 14. Data collection and structure refinement for $[1,3,5\text{-}C_6H_3(NH_3)_3][(SbF_6)_3]\cdot HF$.

	$[1,3,5\text{-}C_6H_3(NH_3)_3][(SbF_6)_3]\cdot HF$	
**Chemical formula**	$C_6\ H_{13}\ F_{19}\ N_3\ Sb_3$	
**Formula weight**	853.47 g/mol	
**Temperature**	103(2) K	
**Wavelength**	0.71073 Å	
**Crystal size**	0.399 x 0.067 x 0.044 mm	
**Crystal habit**	colorless needle	
**Crystal system**	monoclinic	
**Space group**	$P\ 2_1/n$	
**Unit cell dimensions**	a = 14.3329(8) Å	α = 90°
	b = 9.8824(5) Å	β = 115.686(5)°
	c = 16.0142(8) Å	γ = 90°
**Volume**	647.53(7) $Å^3$	
**Z**	2	
**Density (calculated)**	2.773 g/cm^3	
**Absorption coefficient**	4.104 mm^{-1}	
**F(000)**	1576	
**Diffractometer**	Oxford XCalibur	
**Radiation source**	MoKα, λ = 0.71073 Å	
**Index ranges**	-9<=h<=9, -12<=k<=12, -17<=l<=18	
**Reflections collected**	4519	
**Absorption correction**	multi-scan	
**Max. and min. transmission**	1.000 and 0.773	

**Structure solution program**	SHELXT 2018/3 (Sheldrick, 2018)	
**Refinement method**	Full-matrix least-squares on F^2	
**Refinement program**	SHELXL-2018/3 (Sheldrick, 2018)	
**Goodness-of-fit on F^2**	1.041	
**Final R indices**	3549 data; I>2σ(I)	R1 = 0.0385, wR2 = 0.0896
	all data	R1 = 0.0571
**Weighting scheme**	w=1/[¥s^2^(Fo^2^)+(0.0354P)^2^] where P=(Fo^2^+2Fc^2^)/3	
**Largest diff. peak and hole**	1.234 and -0.568 eÅ^{-3}	
**R.M.S. deviation from mean**	0.181 eÅ^{-3}	
**CCDC-deposition number**	2085062	

Table 15. Bond lengths (Å) of [1,3,5-$C_6H_3(NH_3)_3$][$(SbF_6)_3$] · HF.

N1	C2	1.460(7)
N3	C6	1.480(7)
N2	C4	1.484(7)
C4	C5	1.368(8)
C4	C3	1.372(7)
C5	C6	1.383(8)
C6	C1	1.374(8)
C2	C1	1.375(8)
C2	C3	1.385(8)
Sb2	F12	1.862(4)
Sb2	F10	1.864(3)
Sb2	F9	1.868(4)
Sb2	F8	1.885(4)
Sb2	F7	1.895(3)
Sb2	F11	1.914(4)
Sb1	F2	1.860(4)
Sb1	F5	1.866(3)
Sb1	F4	1.869(4)
Sb1	F1	1.870(4)
Sb1	F6	1.873(3)
Sb1	F3	1.874(4)
Sb3	F17	1.868(4)

Sb3	F14	1.872(3)
Sb3	F18	1.875(4)
Sb3	F15	1.880(3)
Sb3	F13	1.891(4)
Sb3	F16	1.985(5)

Table 16. Bond angles (°) for [1,3,5-$C_6H_3(NH_3)_3$][$(SbF_6)_3$] · HF.

C5	C4	C3	122.8(5)
C5	C4	N2	118.1(5)
C3	C4	N2	119.0(5)
C4	C5	C6	117.3(5)
C1	C6	C5	122.8(5)
C1	C6	N3	119.3(5)
C5	C6	N3	118.0(5)
C1	C2	C3	122.6(5)
C1	C2	N1	118.8(5)
C3	C2	N1	118.6(5)
C4	C3	C2	117.3(5)
C6	C1	C2	117.1(5)
F12	Sb2	F10	90.28(18)
F12	Sb2	F9	178.4(2)
F10	Sb2	F9	90.31(17)
F12	Sb2	F8	89.1(2)
F10	Sb2	F8	91.60(17)
F9	Sb2	F8	89.4(2)
F12	Sb2	F7	90.16(16)
F10	Sb2	F7	179.18(18)
F9	Sb2	F7	89.23(16)
F8	Sb2	F7	87.72(16)
F12	Sb2	F11	90.5(2)
F10	Sb2	F11	91.09(17)
F9	Sb2	F11	91.0(2)
F8	Sb2	F11	177.28(18)
F7	Sb2	F11	89.59(16)

F2	Sb1	F5	179.61(19<br>)
F2	Sb1	F4	90.53(19)
F5	Sb1	F4	89.79(18)
F2	Sb1	F1	89.31(18)
F5	Sb1	F1	90.37(17)
F4	Sb1	F1	179.50(17<br>)
F2	Sb1	F6	91.17(18)
F5	Sb1	F6	89.06(17)
F4	Sb1	F6	89.84(17)
F1	Sb1	F6	90.63(18)
F2	Sb1	F3	90.2(2)
F5	Sb1	F3	89.61(19)
F4	Sb1	F3	89.60(18)
F1	Sb1	F3	89.94(18)
F6	Sb1	F3	178.6(2)
F17	Sb3	F14	176.8(2)
F17	Sb3	F18	90.44(18)
F14	Sb3	F18	88.22(18)
F17	Sb3	F15	90.46(17)
F14	Sb3	F15	90.61(17)
F18	Sb3	F15	174.71(19<br>)
F17	Sb3	F13	89.4(2)
F14	Sb3	F13	87.7(2)
F18	Sb3	F13	86.94(19)
F15	Sb3	F13	87.86(17)
F17	Sb3	F16	88.6(2)
F14	Sb3	F16	94.4(2)
F18	Sb3	F16	91.7(2)
F15	Sb3	F16	93.49(18)
F13	Sb3	F16	177.5(2)

Table 17. Anisotropic atomic displacement parameters (Å^2) for [1,3,5-$C_6H_3(NH_3)_3$][$(SbF_6)_3$] · HF.

	U_{11}	U_{22}	U_{33}	U_{23}	U_{13}	U_{12}
N1	0.037(3)	0.018(3)	0.032(3)	-0.006(2)	0.021(2)	-0.003(2)
N3	0.034(3)	0.023(3)	0.027(3)	0.001(2)	0.018(2)	-0.004(2)
N2	0.032(3)	0.023(3)	0.026(3)	0.001(2)	0.018(2)	0.003(2)
C4	0.020(3)	0.023(3)	0.013(3)	0.002(2)	0.009(2)	0.002(2)
C5	0.020(3)	0.022(3)	0.016(3)	0.001(2)	0.006(2)	0.003(2)
C6	0.019(3)	0.018(3)	0.024(3)	0.004(2)	0.013(2)	-0.001(2)
C2	0.017(3)	0.021(3)	0.021(3)	-0.002(2)	0.007(2)	-0.001(2)
C3	0.024(3)	0.016(3)	0.023(3)	0.004(2)	0.012(2)	0.002(2)
C1	0.024(3)	0.019(3)	0.020(3)	0.003(2)	0.011(2)	0.003(2)
Sb2	0.0245(2)	0.0196(2)	0.0201(2)	-0.00160(15)	0.00778(16)	0.00048(16)
F7	0.026(2)	0.0294(19)	0.0329(19)	-0.0106(15)	0.0109(16)	-0.0061(15)
F10	0.030(2)	0.032(2)	0.033(2)	-0.0100(16)	0.0007(17)	-0.0027(16)
F9	0.028(2)	0.054(3)	0.056(3)	-0.027(2)	0.0089(19)	0.0056(19)
F11	0.094(4)	0.041(3)	0.040(2)	-0.009(2)	0.024(2)	-0.019(2)
F12	0.028(2)	0.064(3)	0.068(3)	-0.038(2)	0.010(2)	0.009(2)
F8	0.094(4)	0.039(2)	0.029(2)	0.0055(18)	0.009(2)	-0.029(2)
Sb1	0.0228(2)	0.0234(2)	0.0186(2)	0.00169(15)	0.01026(16)	0.00017(16)
F1	0.031(2)	0.044(2)	0.037(2)	-0.0089(17)	0.0226(17)	-0.0054(17)
F5	0.030(2)	0.035(2)	0.051(2)	-0.0124(18)	0.0146(18)	-0.0109(17)
F6	0.060(3)	0.032(2)	0.030(2)	0.0075(16)	0.0208(19)	0.0114(18)
F4	0.038(3)	0.060(3)	0.039(2)	0.0092(19)	0.0262(19)	0.012(2)
F2	0.045(3)	0.064(3)	0.031(2)	-0.0260(19)	0.0114(18)	-0.011(2)
F3	0.058(3)	0.061(3)	0.047(2)	0.033(2)	0.031(2)	0.022(2)
Sb3	0.0275(3)	0.0189(2)	0.0239(2)	-0.00189(15)	0.00936(17)	-0.00164(16)
F15	0.037(2)	0.028(2)	0.041(2)	-0.0127(16)	0.0139(18)	-0.0108(16)
F18	0.053(3)	0.025(2)	0.056(3)	-0.0142(18)	0.010(2)	-0.0114(18)
F17	0.056(3)	0.040(2)	0.042(2)	0.0151(19)	-0.007(2)	0.000(2)
F16	0.035(3)	0.032(2)	0.145(5)	-0.010(3)	0.059(3)	0.0061(18)
F13	0.045(3)	0.035(2)	0.074(3)	-0.004(2)	0.041(2)	-0.0037(19)
F14	0.059(3)	0.035(2)	0.033(2)	0.0078(17)	0.006(2)	0.009(2)

F19	0.034(2)	0.028(2)	0.046(2)	-0.0066(16)	0.0196(18)	-0.0051(16)

3.3 Comparison of bond lengths

Table 18. Comparison of bond lengths of $[1,3,5\text{-}C_6H_3(CNH)_3][(SbF_6)(Sb_2F_{11})_2]\cdot 3HF$ with the starting material[10] and $[1,3,5\text{-}C_6H_3(NH_3)_3][(SbF_6)_3]\cdot HF$.

$[1,3,5\text{-}C_6H_3(CNH)_3][(SbF_6)(Sb_2F_{11})_2]\cdot 3HF$.		
bond lengths [Å]	starting material[10]	$+3H^+$
C≡N	1.131(4)<br>1.135(3)<br>1.134(3)	1.125(7)<br>1.135(8)<br>1.141(8)
C–C(N)	1.447(3)<br>1.440(3)<br>1.444(3)	1.424(8)<br>1.426(8)<br>1.442(7)
CC (aromatic)	1.386(3)<br>1.387(3)<br>1.388(3)<br>1.390(2)<br>1.387(3)<br>1.390(3)	1.398(7)<br>1.382(8)<br>1.387(8)<br>1.399(7)<br>1.399(8)<br>1.390(6)
$[1,3,5\text{-}C_6H_3(NH_3)_3][(SbF_6)_3]\cdot HF$		
bond lengths [Å]	starting material	$+3H^+$
C–N	-	1.484(9)<br>1.480(8)<br>1.459(8)
CC (aromatic)	-	1.39(1)<br>1.375(9)<br>1.375(7)<br>1.38(1)<br>1.368(9)<br>1.372(8)

Protonation of 1,3,5-Tricyanobenzene has no significant effect on bond lengths.

As for 1,3,5-Triaminobenzene has no entry for a crystal structure of the neutral compound, attempts for recrystallization showed, that the hydrochloride is fact not the HCl adduct but

the triammonium trichloride salt. Addition of base, specifically NaOH, during recrystallization with water led to the formation of $Na_2CO_3 \cdot H_2O$, indicating decomposition of the aromatic compound, making the attempts of crystaillzation of the free amine futile.

4. NMR data

4.1 1,3,5-Tricyanobenzene

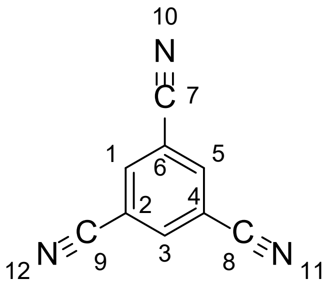

NMR (*a*HF, 26 °C) (ppm): δ (^{1}H) = 8.72 (3H, s, H-1, H-3, H-5); δ(^{13}C) = 141.07 (s, C-1, C-3, C-5), 113.78 (s, C-2, C-4, C-6), 111.17 (s, C-7, C-8, C-9); δ (^{14}N) = –238.79 (s(broad), N-10, N-11, N-12).

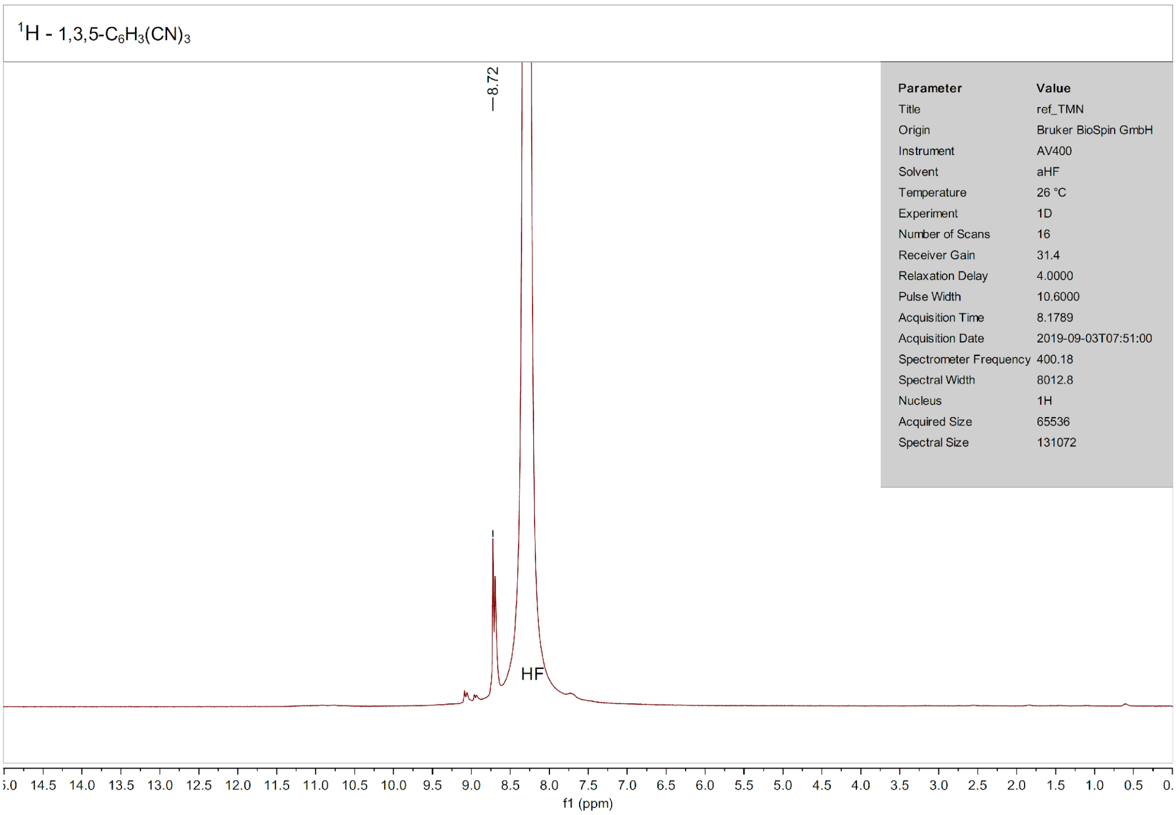
1H - 1,3,5-C6H3(CN)3
8.72
HF
Parameter Value
Title ref_TMN
Origin Bruker BioSpin GmbH
Instrument AV400
Solvent aHF
Temperature 26 °C
Experiment 1D
Number of Scans 16
Receiver Gain 31.4
Relaxation Delay 4.0000
Pulse Width 10.6000
Acquisition Time 8.1789
Acquisition Date 2019-09-03T07:51:00
Spectrometer Frequency 400.18
Spectral Width 8012.8
Nucleus 1H
Acquired Size 65536
Spectral Size 131072
f1 (ppm)

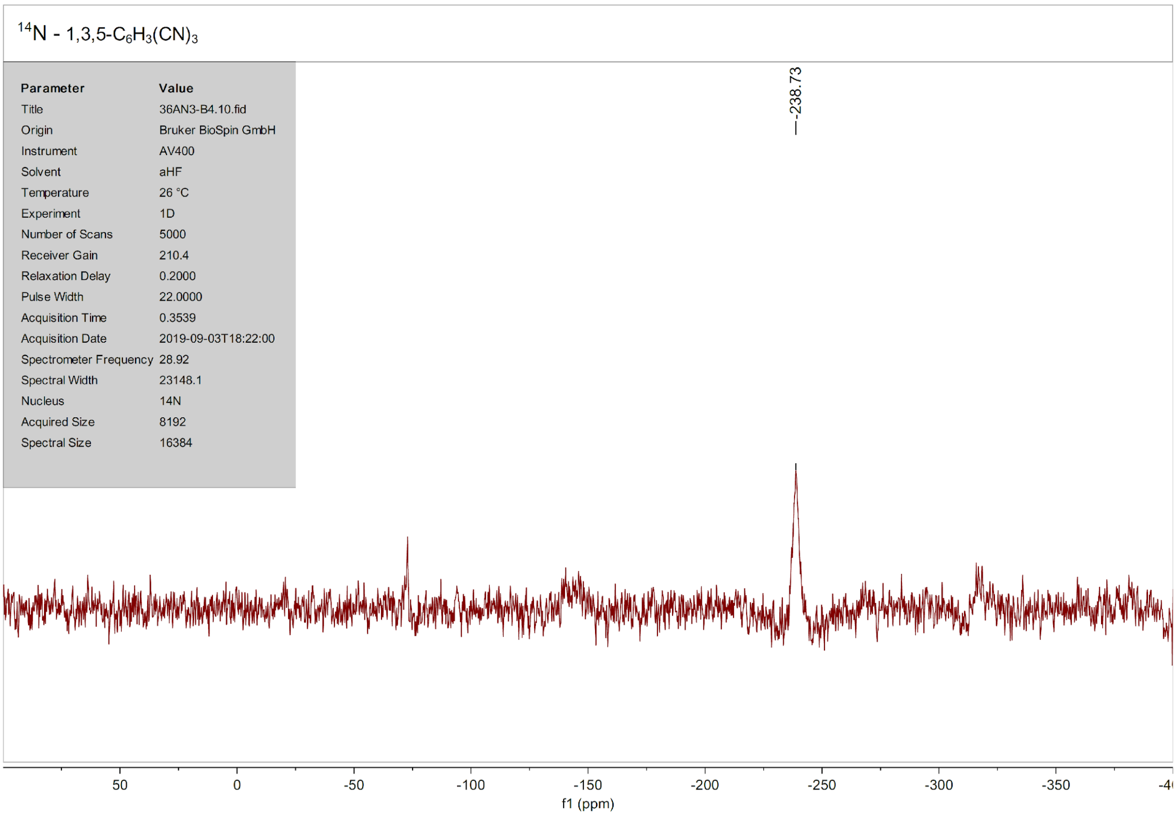
14N - 1,3,5-C6H3(CN)3
-238.73
Parameter Value
Title 36AN3-B4.10.fid
Origin Bruker BioSpin GmbH
Instrument AV400
Solvent aHF
Temperature 26 °C
Experiment 1D
Number of Scans 5000
Receiver Gain 210.4
Relaxation Delay 0.2000
Pulse Width 22.0000
Acquisition Time 0.3539
Acquisition Date 2019-09-03T18:22:00
Spectrometer Frequency 28.92
Spectral Width 23148.1
Nucleus 14N
Acquired Size 8192
Spectral Size 16384
f1 (ppm)

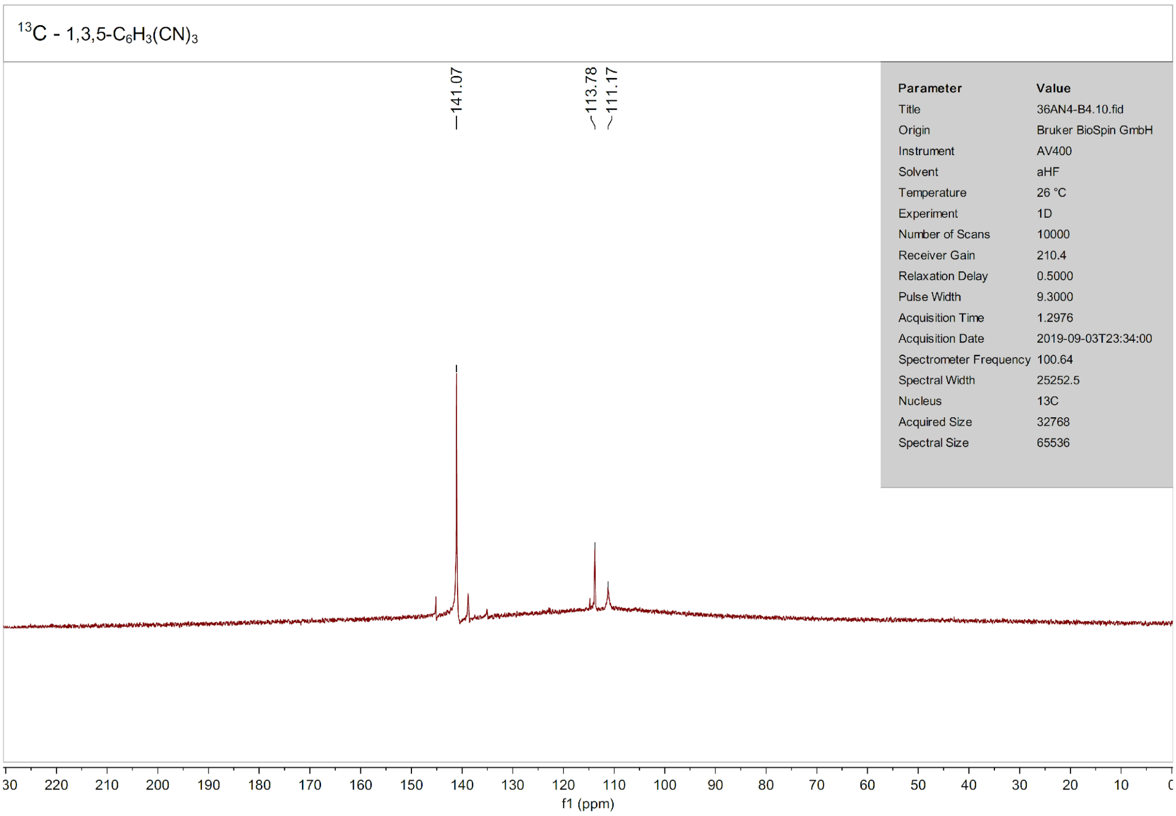

13C - 1,3,5-C6H3(CN)3
141.07
113.78
111.17
Parameter
Value
Title
36AN4-B4.10.fid
Origin
Bruker BioSpin GmbH
Instrument
AV400
Solvent
aHF
Temperature
26 °C
Experiment
1D
Number of Scans
10000
Receiver Gain
210.4
Relaxation Delay
0.5000
Pulse Width
9.3000
Acquisition Time
1.2976
Acquisition Date
2019-09-03T23:34:00
Spectrometer Frequency
100.64
Spectral Width
25252.5
Nucleus
13C
Acquired Size
32768
Spectral Size
65536
f1 (ppm)

4.2 $[1,3,5-C_6H_3(CNH)_3][(SbF_6)_3]$

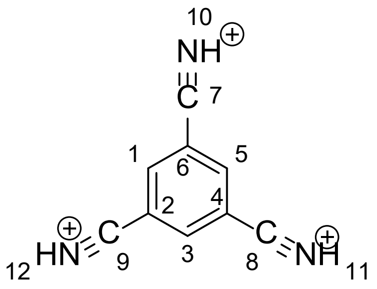

NMR (*a*HF, 0 ° C) (ppm): δ (^{1}H) = 9.84 (3H, s, H-10, H-11, H-12), 9.03 (3H, s, H-1, H-3, H-5); δ (^{13}C) = 150.39 (s, C-1, C-3, C-5), 109.13 (s, C-2, C-4, C-6), 100.08 (s, C-7, C-8, C-9); δ (^{14}N) = –214.68 (s(broad), N-10, N-11, N-12); δ(^{19}F) = –119.70, –124.65 (SbF_6^-).

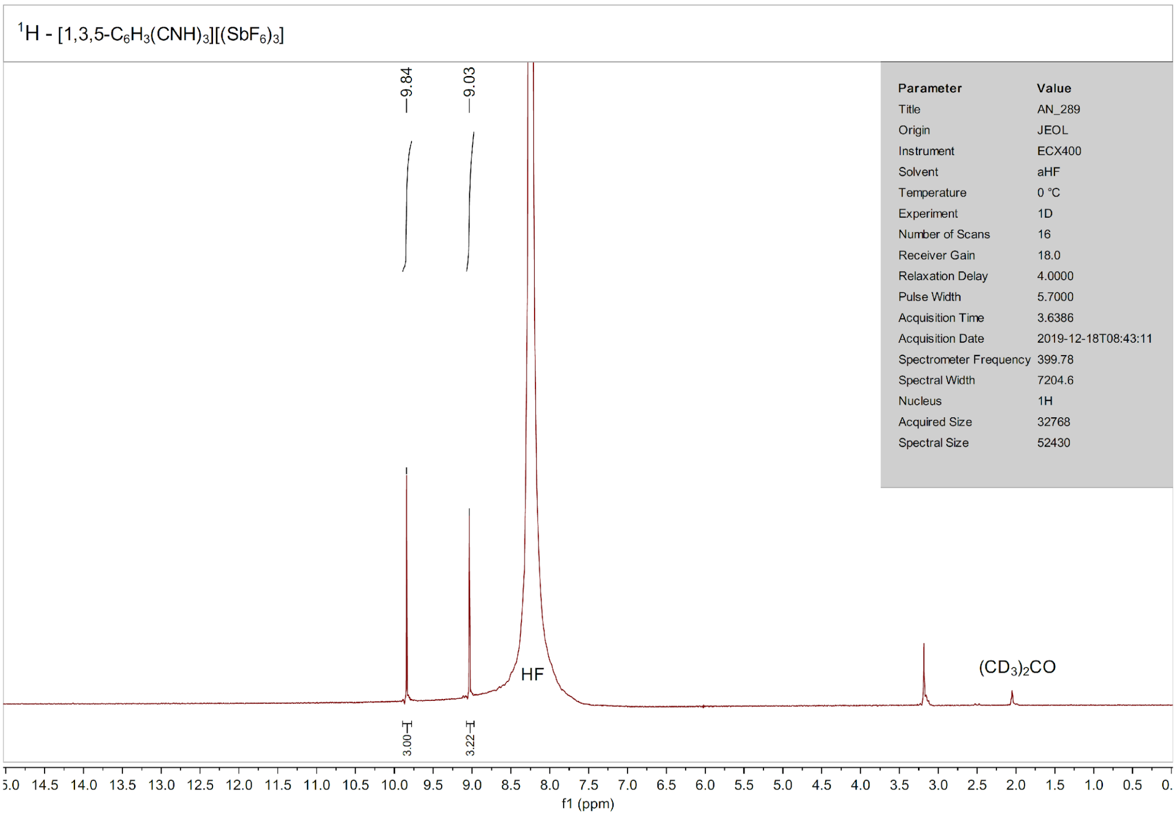
1H - [1,3,5-C6H3(CNH)3][(SbF6)3]
9.84
9.03
HF
(CD3)2CO
3.00
3.22
f1 (ppm)
Parameter Value
Title AN_289
Origin JEOL
Instrument ECX400
Solvent aHF
Temperature 0 °C
Experiment 1D
Number of Scans 16
Receiver Gain 18.0
Relaxation Delay 4.0000
Pulse Width 5.7000
Acquisition Time 3.6386
Acquisition Date 2019-12-18T08:43:11
Spectrometer Frequency 399.78
Spectral Width 7204.6
Nucleus 1H
Acquired Size 32768
Spectral Size 52430

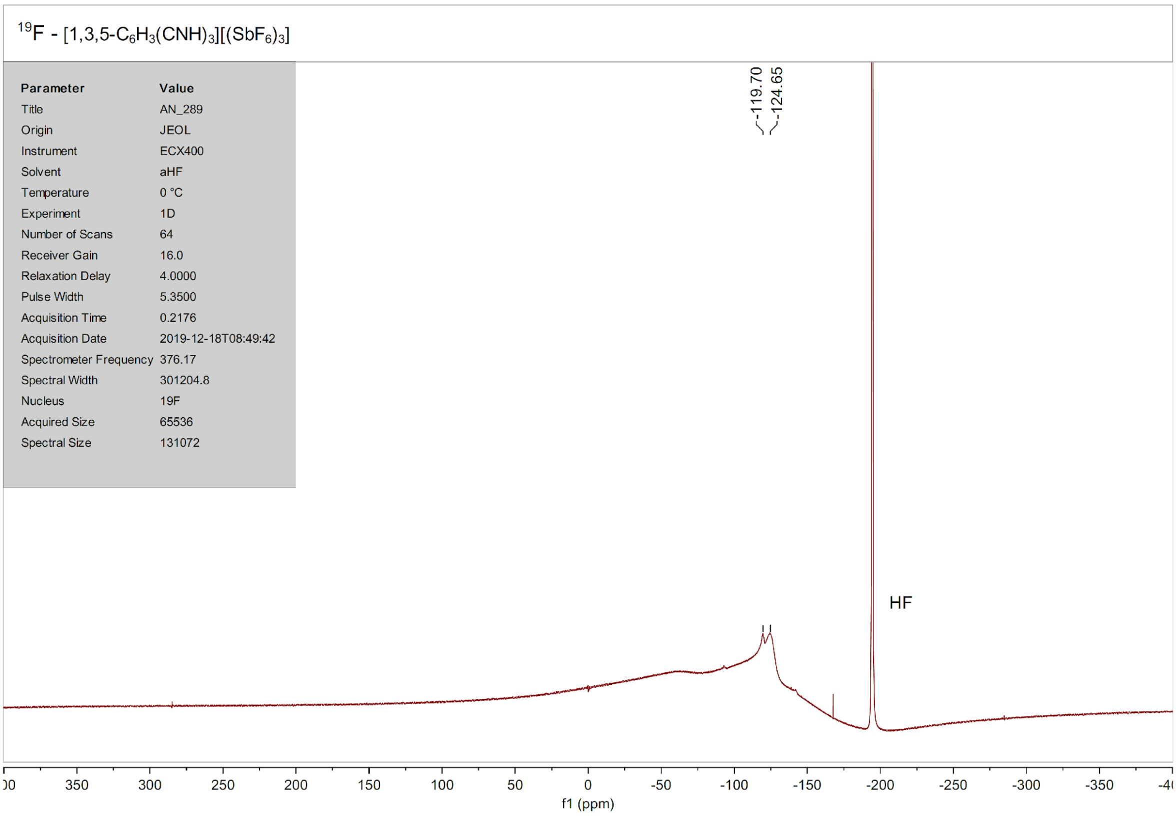
19F - [1,3,5-C6H3(CNH)3][(SbF6)3]
-119.70
-124.65
HF
f1 (ppm)
Parameter Value
Title AN_289
Origin JEOL
Instrument ECX400
Solvent aHF
Temperature 0 °C
Experiment 1D
Number of Scans 64
Receiver Gain 16.0
Relaxation Delay 4.0000
Pulse Width 5.3500
Acquisition Time 0.2176
Acquisition Date 2019-12-18T08:49:42
Spectrometer Frequency 376.17
Spectral Width 301204.8
Nucleus 19F
Acquired Size 65536
Spectral Size 131072

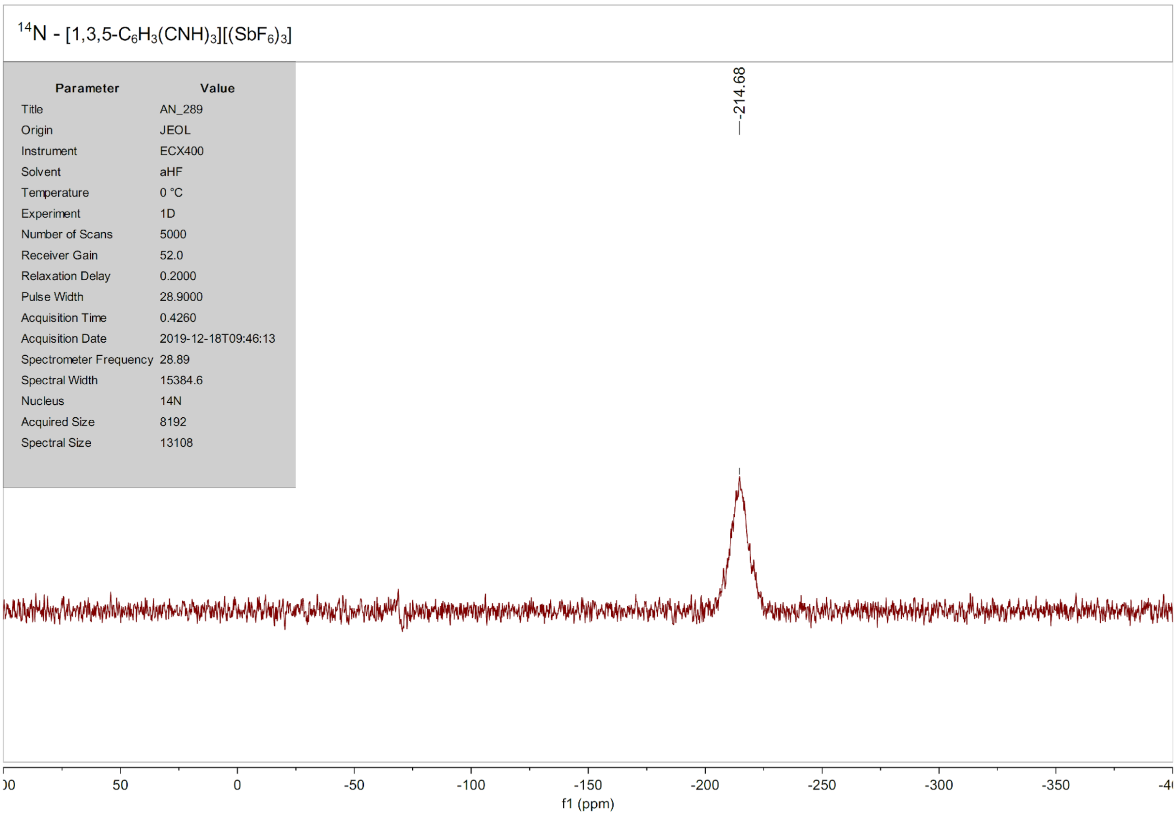

14N - [1,3,5-C6H3(CNH)3][(SbF6)3]
Parameter Value
Title AN_289
Origin JEOL
Instrument ECX400
Solvent aHF
Temperature 0 °C
Experiment 1D
Number of Scans 5000
Receiver Gain 52.0
Relaxation Delay 0.2000
Pulse Width 28.9000
Acquisition Time 0.4260
Acquisition Date 2019-12-18T09:46:13
Spectrometer Frequency 28.89
Spectral Width 15384.6
Nucleus 14N
Acquired Size 8192
Spectral Size 13108
-214.68
50 0 -50 -100 -150 -200 -250 -300 -350
f1 (ppm)

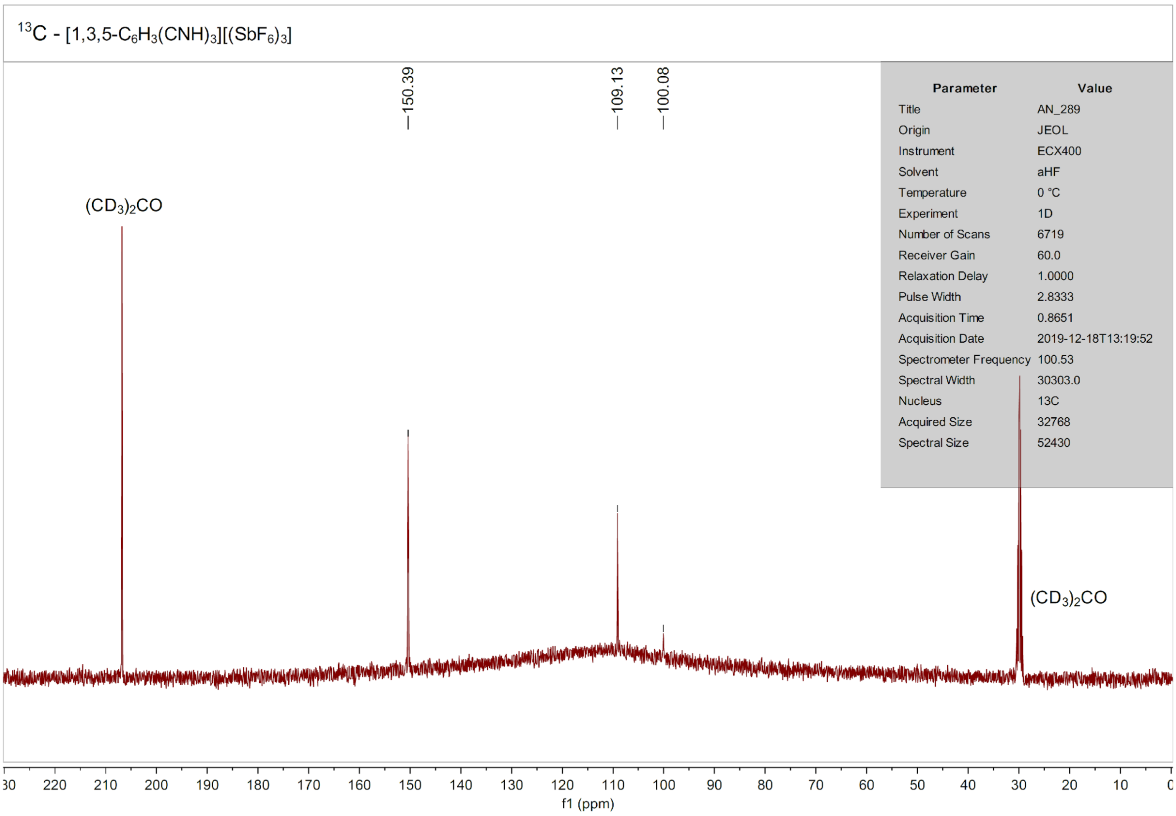

13C - [1,3,5-C6H3(CNH)3][(SbF6)3]
150.39
109.13
100.08
(CD3)2CO
Parameter Value
Title AN_289
Origin JEOL
Instrument ECX400
Solvent aHF
Temperature 0 °C
Experiment 1D
Number of Scans 6719
Receiver Gain 60.0
Relaxation Delay 1.0000
Pulse Width 2.8333
Acquisition Time 0.8651
Acquisition Date 2019-12-18T13:19:52
Spectrometer Frequency 100.53
Spectral Width 30303.0
Nucleus 13C
Acquired Size 32768
Spectral Size 52430
(CD3)2CO
220 210 200 190 180 170 160 150 140 130 120 110 100 90 80 70 60 50 40 30 20 10
f1 (ppm)

4.3 $[1,3,5\text{-}C_6H_3(CNCH_3)_3][(SbF_6)_3]$

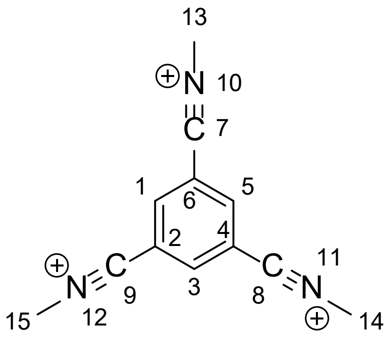

NMR (SO_2, –20 ° C) (ppm): δ (^{1}H) = 10.49 (3H, s, H-1, H-3, H-5), 5.44 (9H, s, H-13abc, H-14abc, H-15abc); δ (^{13}C) = 150.09 (s, C-1, C-3, C-5), 111.25 (s, C-2, C-4, C-6), 100.90 (s, C-7, C-8, C-9), 33.97 (s, C-13, C-14, C-15); δ (^{14}N) = –215.09 (s(broad), N-10, N-11, N-12); δ (^{19}F) = –105.33 (SbF_6^-).

^1H - $[1,3,5\text{-}C_6H_3(CNCH_3)_3][(SbF_6)_3]$

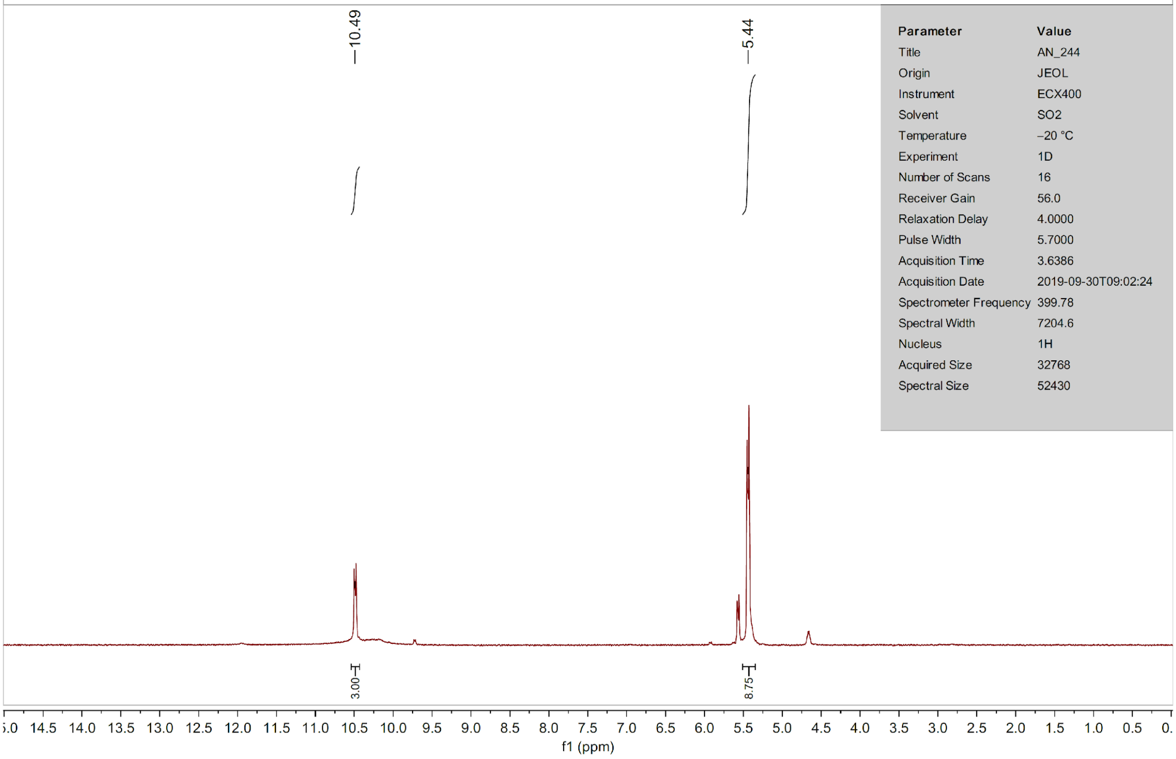

^{19}F - $[1,3,5\text{-}C_6H_3(CNCH_3)_3][(SbF_6)_3]$

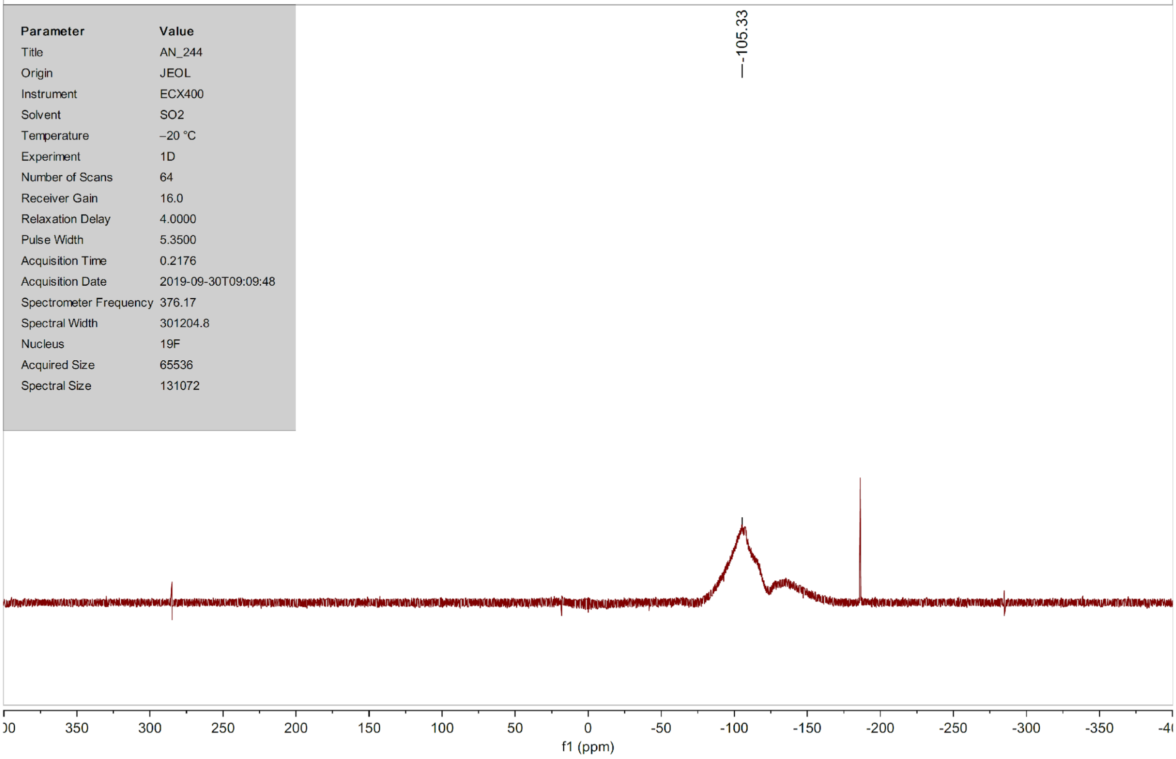

^{14}N - $[1,3,5-C_6H_3(CNCH_3)_3][(SbF_6)_3]$

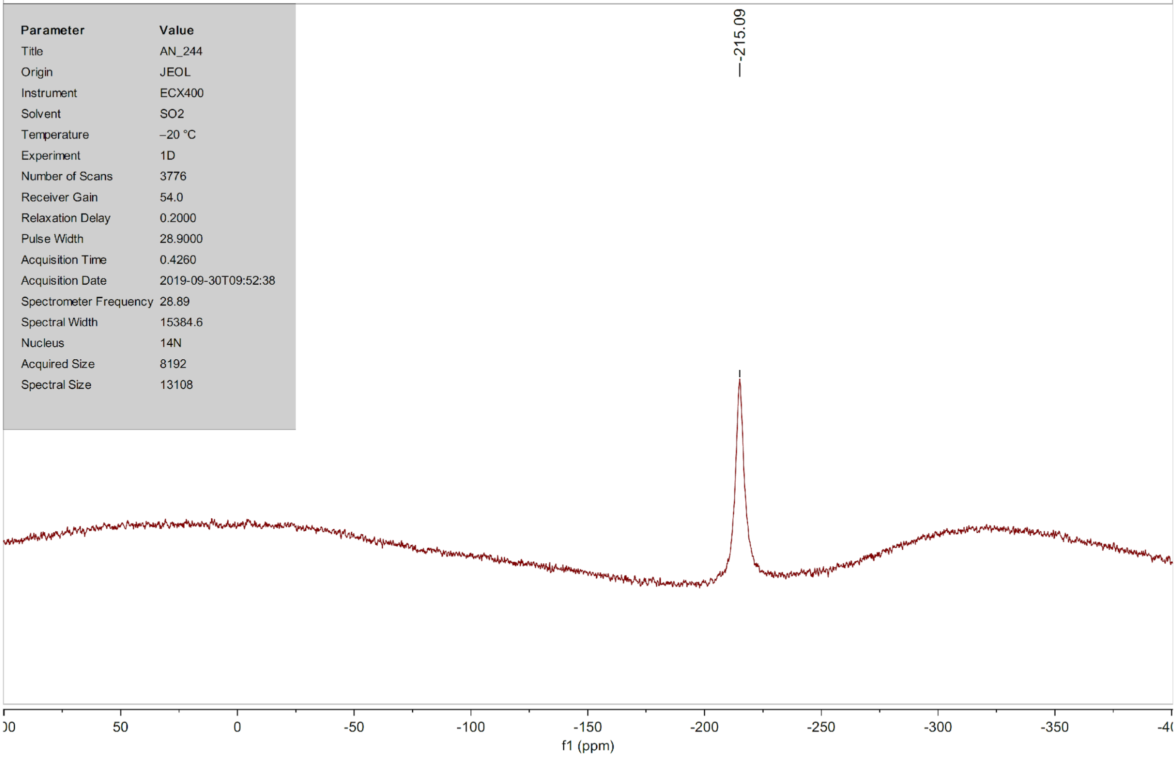

^{13}C - $[1,3,5-C_6H_3(CNCH_3)_3][(SbF_6)_3]$

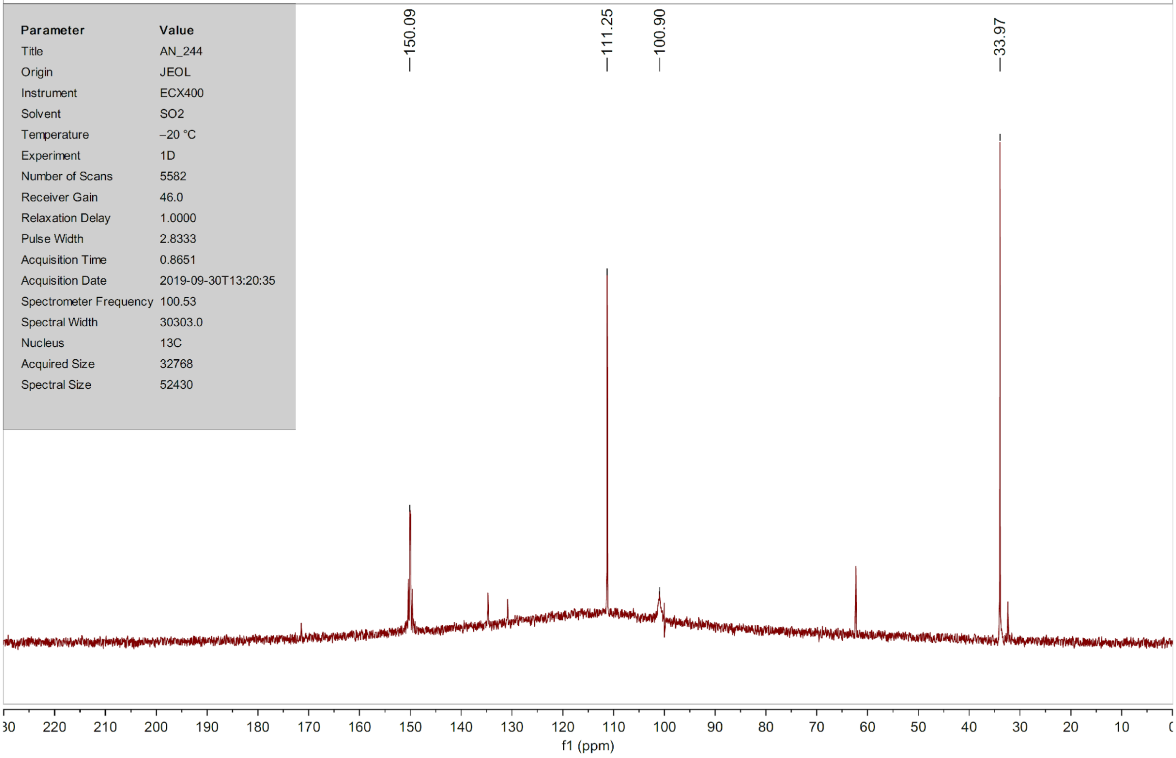

4.4 Comparison of NMR data of 1,3,5-Tricyanobenzene and its` protonated and methylated derivates

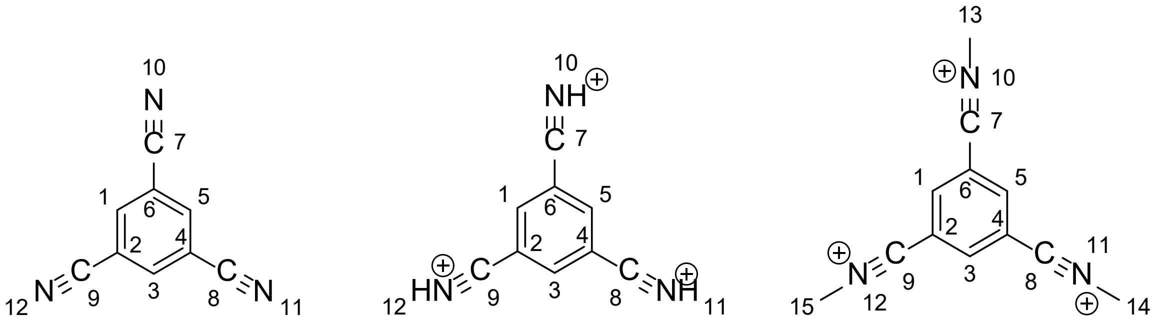

Table 19. Comparison of NMR data of 1,3,5-Tricyanobenzene and its` protonated and methylated derivatives.

[ppm]	^{13}C educt in SO_2 @RT	^{13}C protonated product in HF @0° C (Δ to educt)	^{13}C methylated product SO_2 @–20° C (Δ to educt)
**C**N	111.17	100.08 (–11.09)	100.90 (–10.27)
**C**CN	113.78	109.13 (–4.65)	111.25 (–2.53)
**C**-H	141.07	150.39 (+9.32)	150.09 (+9.02)
N-**C**H_3			33.97
	^{14}N educt in SO_2 @RT (Δ to educt)	^{14}N protonated product in HF @0° C (Δ to educt)	^{14}N methylated product SO_2 @–20° C (Δ to educt)
	–238.79	–214.68 (+24.11)	–215.09 (+23.7)
	^{1}H educt in SO_2 @RT (Δ to educt)	^{1}H protonated product in HF @0° C (Δ to educt)	^{1}H methylated product SO_2 @–20° C (Δ to educt)
C-**H**	8.72	9.03 (+0.31)	10.49 (+1.77)
N-**H**		9.84	
C**H**$_3$			5.44

4.5 1,3,5-Triammoniumbenzene

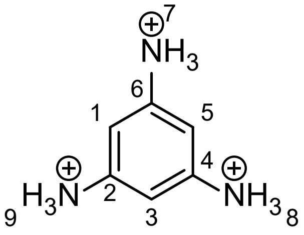

NMR (*a*HF, 26 ° C) (ppm): δ (^{1}H) = 7.55 (3H, s, H-1, H-3, H-5); δ (^{13}C) = 132.07 (s, C-2, C-4, C-6), 121.95 (s, C-1, C-3, C-5); δ (^{14}N) = –333.45 (s(broad), N-7, N-8, N-9).

As the counterion, some sort of fluoride/hydrogenfluoride is to be expected when employing hydrogen fluoride as solvent.

NMR (D_2O, 25° C) (ppm): δ (^{1}H) = 7.03 (3H, s, H-1, H-3, H-5); δ (^{13}C) = 133.02 (s, C-2, C-4, C-6), 110.54 (s, C-1, C-3, C-5); δ (^{14}N) = –356.91 (hept, $^{1}J_{ND}$ = 8.0 Hz), N-7, N-8, N-9).

As the counterion, chloride is to be expected.

Note that in D_2O the compound may be present in two forms - as the ammonium and the arenium ion. First, two peaks appear in ^{14}N NMR, the heptett belonging clearly to a ND_3^+ group, as the observed $^{1}J_{ND}$ with 8 Hz would theoretically result in $^{1}J_{NH}$ = 8 Hz • 6.514 = 52.114 Hz due to relative gyromagnetic ratios appliable for coupling constant calculation.[11] Furthermore, in contrast to N-H couplings, N-D couplings are more likely to be observed in ^{14}N NMR due to a much smaller electric quadrupole moment, reducing contribution to relaxation time.[11] Second, especially observable in ^{13}C NMR spectrum, besides the aromatic signals, more signals appear, the one at 29.66 ppm belonging most likely to the arenium species` CH_2 group.

As discussed in the main paper, in (already slightly) acidic solution, tautomerism between the monoprotonated ammonium species and the arenium is present.[12]

^{1}H - [1,3,5-$C_6H_3(NH_3)_3$][F]

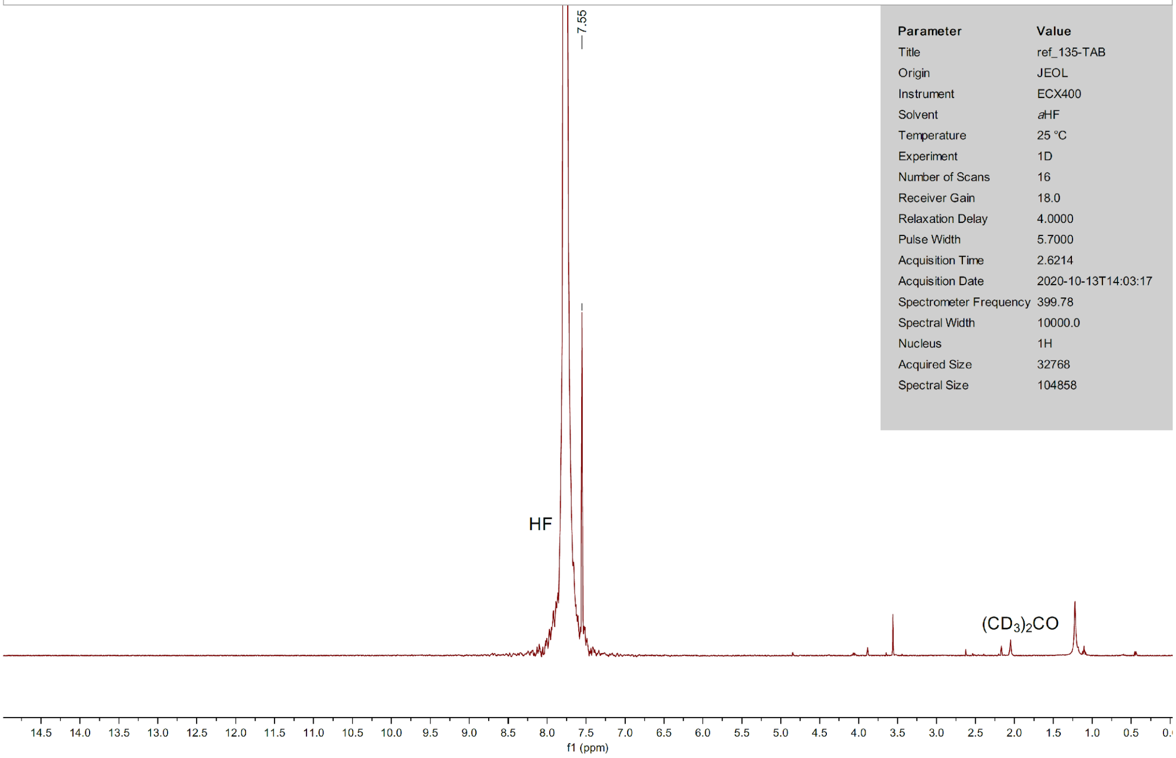

^{14}N - [1,3,5-$C_6H_3(NH_3)_3$][F]

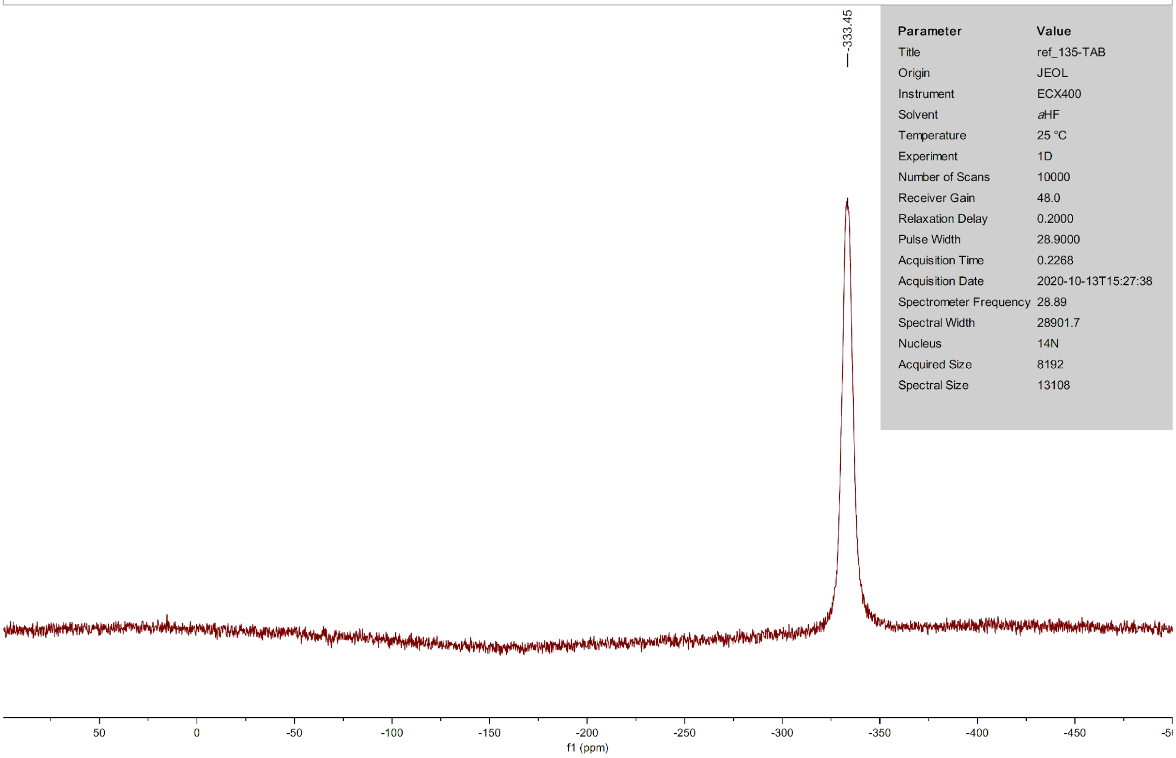

^{14}N - [1,3,5-$C_6H_3(NH_3)_3$][F]

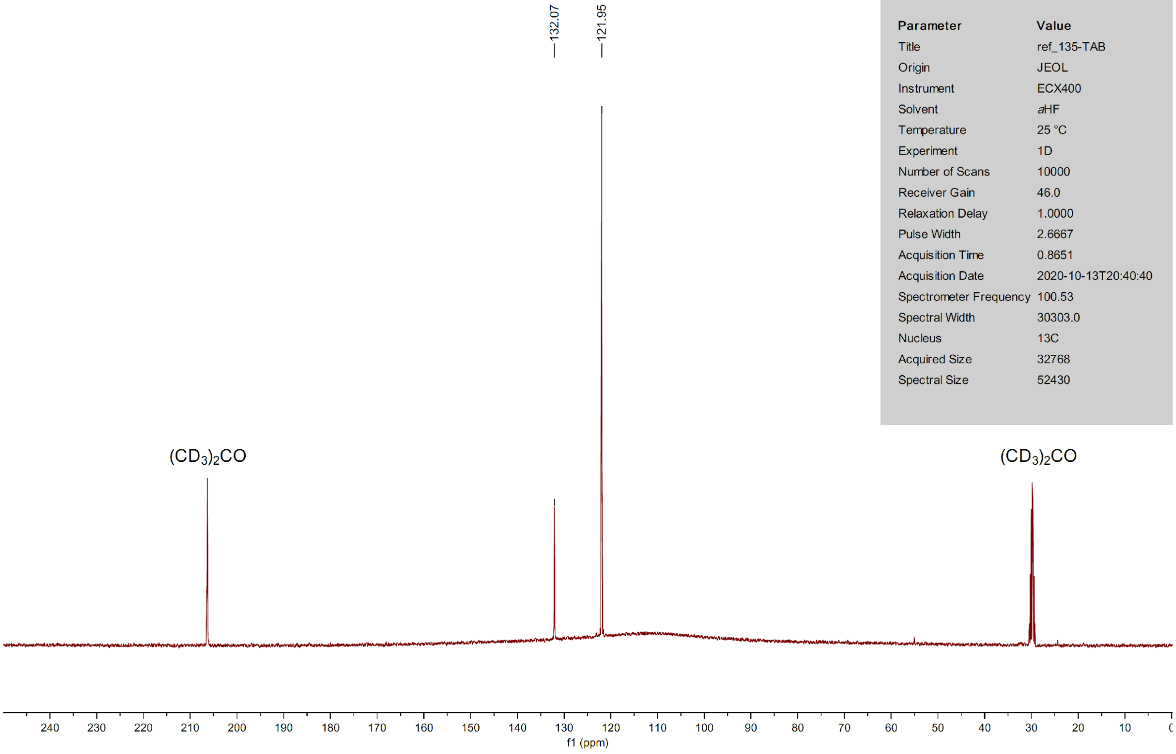

^{1}H - [1,3,5-$C_6H_3(ND_3)_3$][Cl_3]

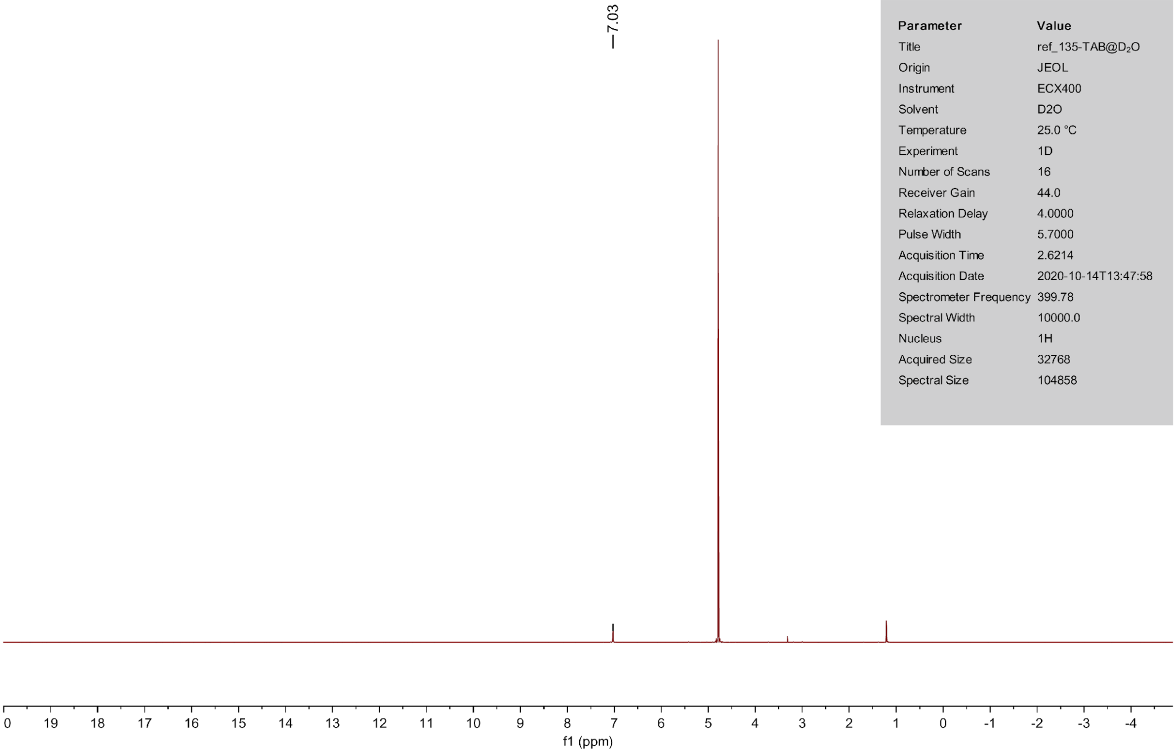

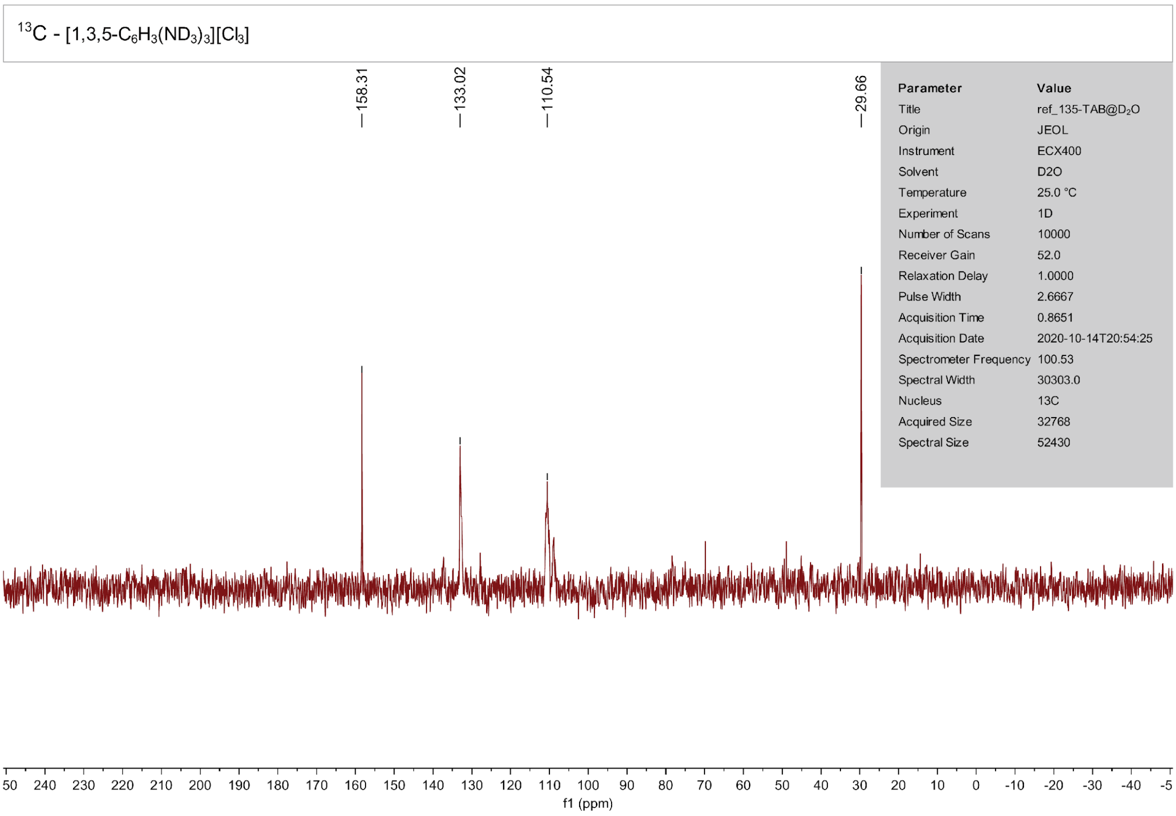

13C - [1,3,5-C6H3(ND3)3][Cl3]
158.31
133.02
110.54
29.66
Parameter Value
Title ref_135-TAB@D2O
Origin JEOL
Instrument ECX400
Solvent D2O
Temperature 25.0 °C
Experiment 1D
Number of Scans 10000
Receiver Gain 52.0
Relaxation Delay 1.0000
Pulse Width 2.6667
Acquisition Time 0.8651
Acquisition Date 2020-10-14T20:54:25
Spectrometer Frequency 100.53
Spectral Width 30303.0
Nucleus 13C
Acquired Size 32768
Spectral Size 52430
f1 (ppm)

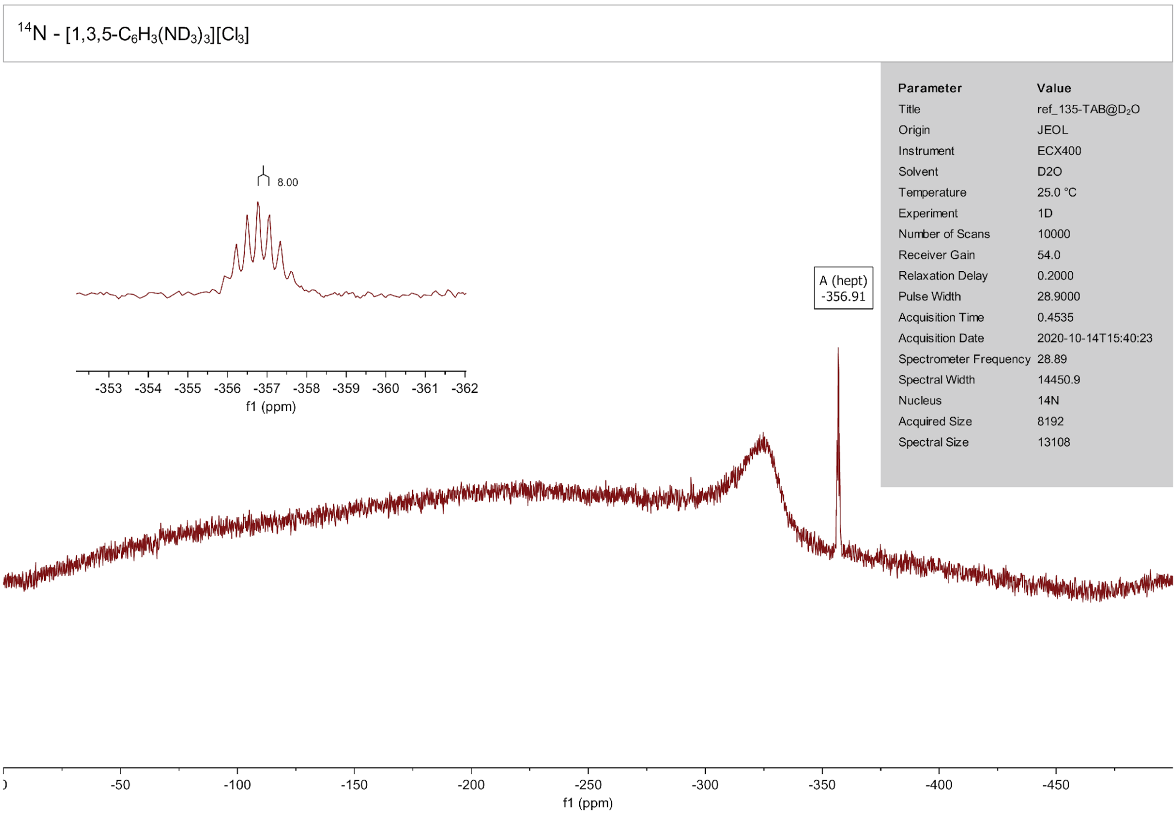

14N - [1,3,5-C6H3(ND3)3][Cl3]
8.00
A (hept)
-356.91
Parameter Value
Title ref_135-TAB@D2O
Origin JEOL
Instrument ECX400
Solvent D2O
Temperature 25.0 °C
Experiment 1D
Number of Scans 10000
Receiver Gain 54.0
Relaxation Delay 0.2000
Pulse Width 28.9000
Acquisition Time 0.4535
Acquisition Date 2020-10-14T15:40:23
Spectrometer Frequency 28.89
Spectral Width 14450.9
Nucleus 14N
Acquired Size 8192
Spectral Size 13108
f1 (ppm)

5. Quantum chemical calculations

5.1 1,3,5-Tricyanobenzene

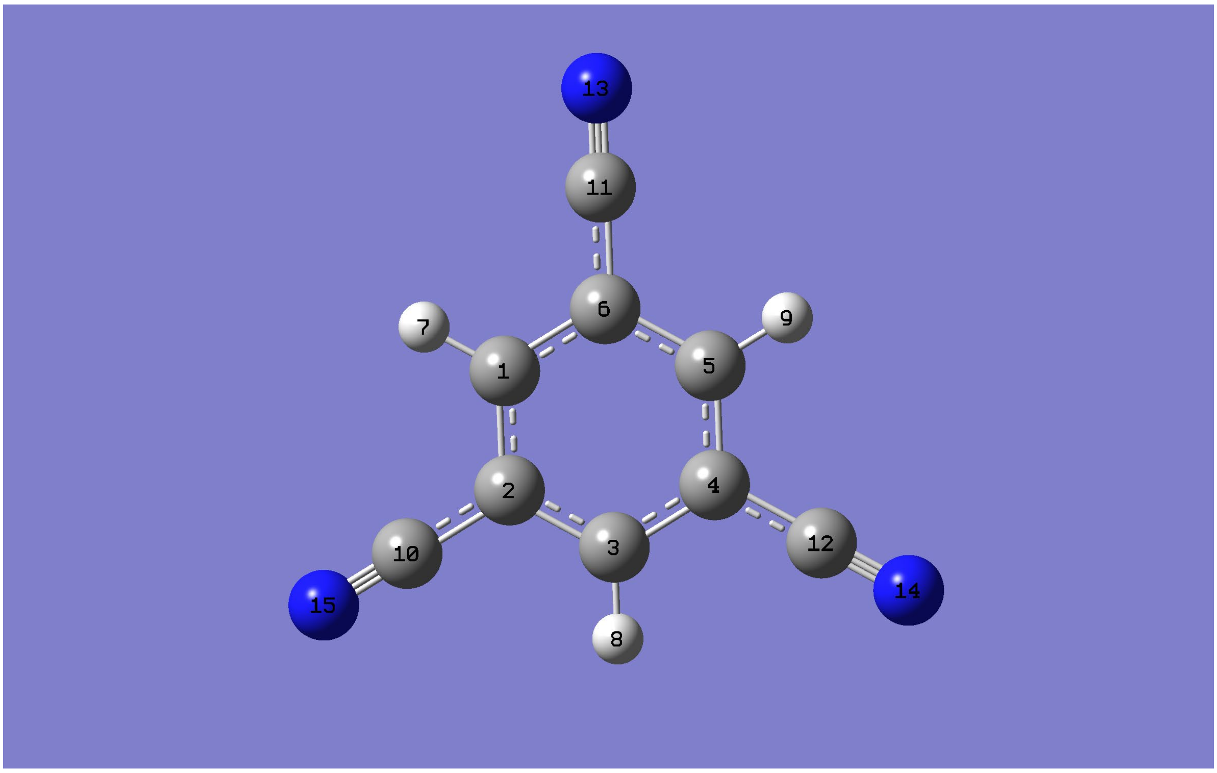

Figure 9. Optimized structure of 1,3,5-Tricyanobenzene.

E(RB3LYP) = -508.956620785 Hartree

Standard orientation:

Center	Atomic	Atomic	Coordinates (Angstroms)		
Number	Number	Type	X	Y	Z
1	6	0	-0.811266	-1.147350	0.000000
2	6	0	0.587026	-1.274132	0.000002

3	6	0	1.399264	-0.128900	0.000002
4	6	0	0.809918	1.145447	-0.000000
5	6	0	-0.588004	1.276248	-0.000002
6	6	0	-1.396947	0.128686	-0.000001
7	1	0	-1.434266	-2.028448	0.000000
8	1	0	2.473817	-0.227887	0.000003
9	1	0	-1.039557	2.256330	-0.000003
10	6	0	1.186282	-2.574773	0.000003
11	6	0	-2.822963	0.260042	-0.000003
12	6	0	1.636683	2.314735	-0.000000
13	7	0	-3.983846	0.366979	-0.000004
14	7	0	2.309742	3.266616	-0.000000
15	7	0	1.674112	-3.633597	0.000005

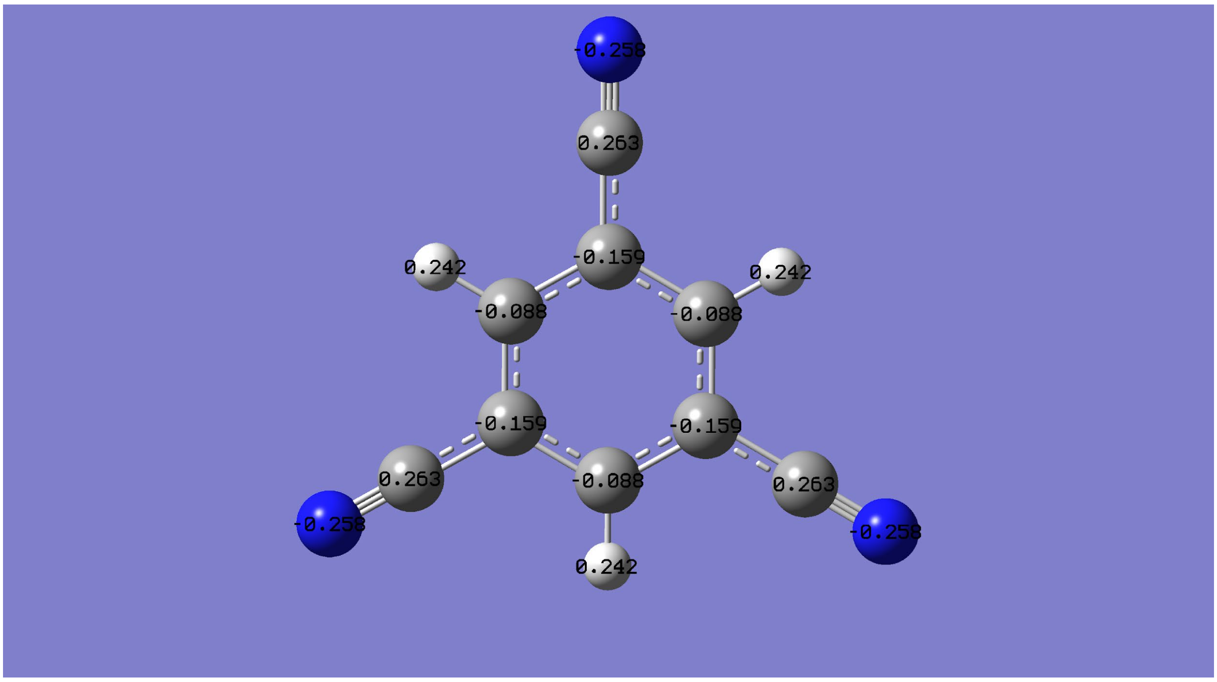

Figure 10. NPA charges of 1,3,5-Tricyanobenzene.

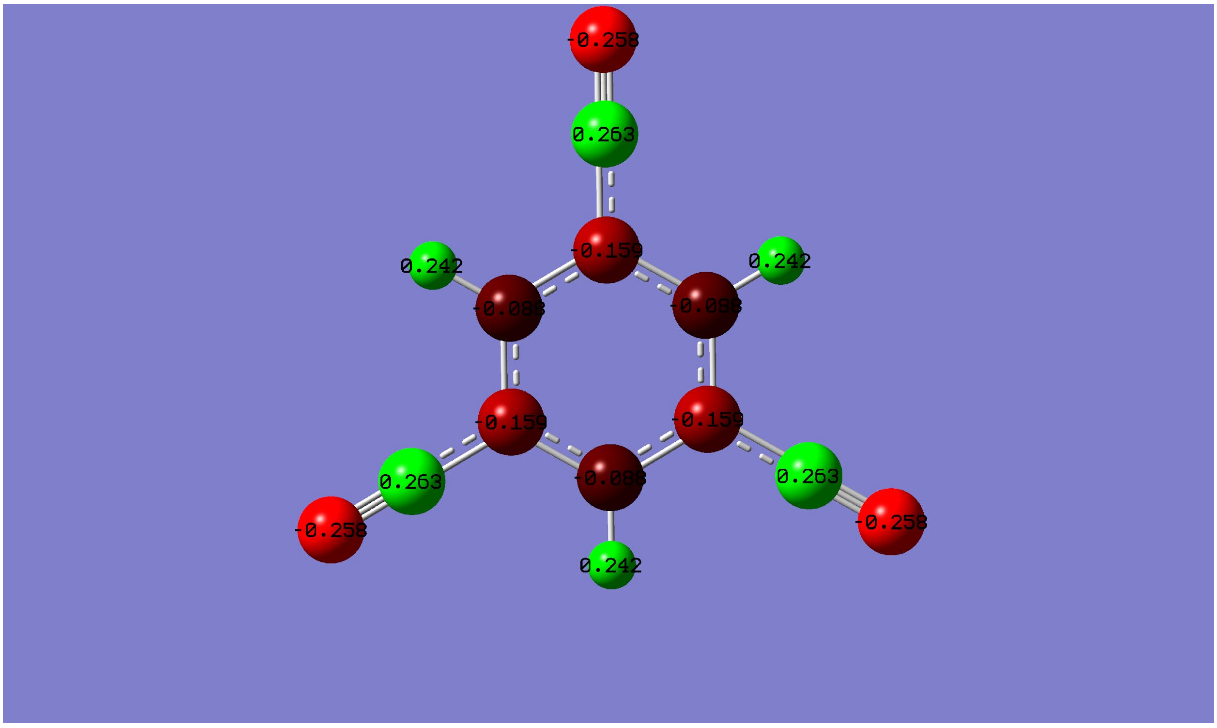

Figure 11. NPA charges of 1,3,5-Tricyanobenzene (coloured).

5.2 1,3,5-$C_6H_3(CNH)_3$

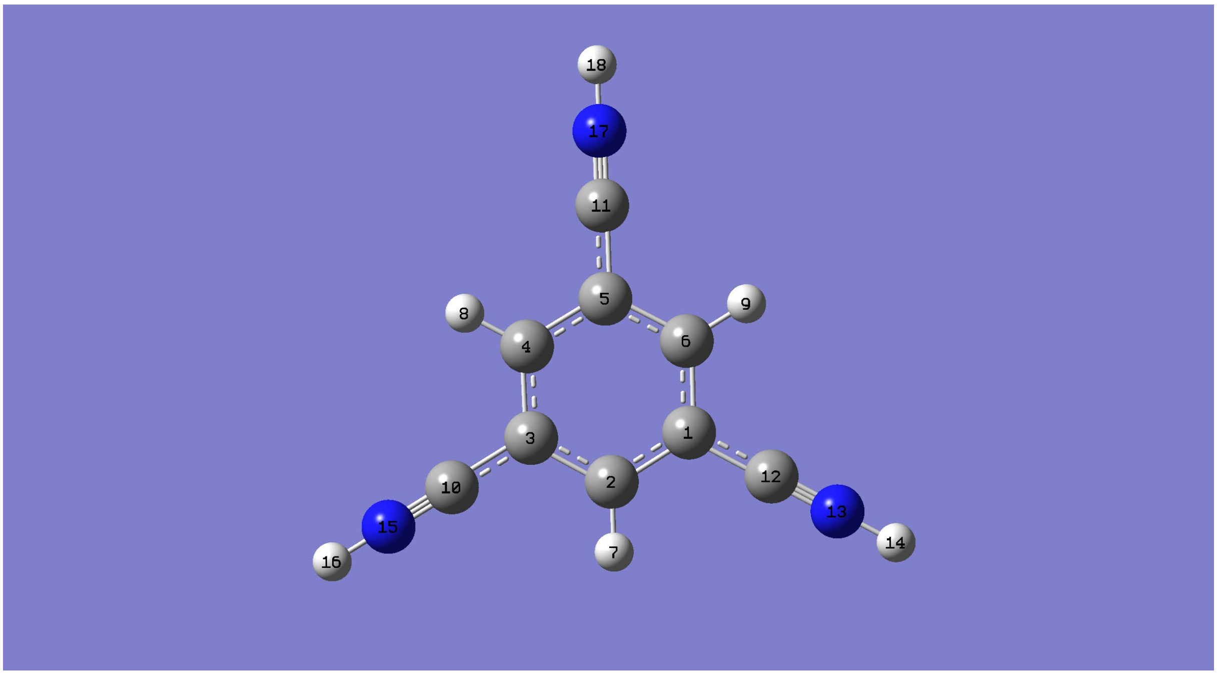

Figure 12. Optimized structure of 1,3,5-$C_6H_3(CNH)_3$.

E(RB3LYP) = -509.564352855 Hartree

Standard orientation:

Center Number	Atomic Number	Atomic Type	Coordinates (Angstroms) X	Y	Z
1	6	0	1.352795	-0.368153	-0.000040
2	6	0	0.361768	-1.371534	0.000043
3	6	0	-0.995220	-0.987479	0.000080
4	6	0	-1.368659	0.372466	0.000043
5	6	0	-0.357568	1.355623	-0.000038

6	6	0	1.006901	0.999060	-0.000082
7	1	0	0.637917	-2.418472	0.000077
8	1	0	-2.413405	0.656781	0.000076
9	1	0	1.775500	1.761678	-0.000150
10	6	0	-2.014494	-1.998804	0.000159
11	6	0	-0.723770	2.744001	-0.000076
12	6	0	2.738265	-0.745200	-0.000082
13	7	0	3.845988	-1.046640	-0.000116
14	1	0	4.830568	-1.314562	-0.000144
15	7	0	-2.829425	-2.807383	0.000220
16	1	0	-3.553777	-3.526057	0.000275
17	7	0	-1.016573	3.854036	-0.000110
18	1	0	-1.276833	4.840668	-0.000137

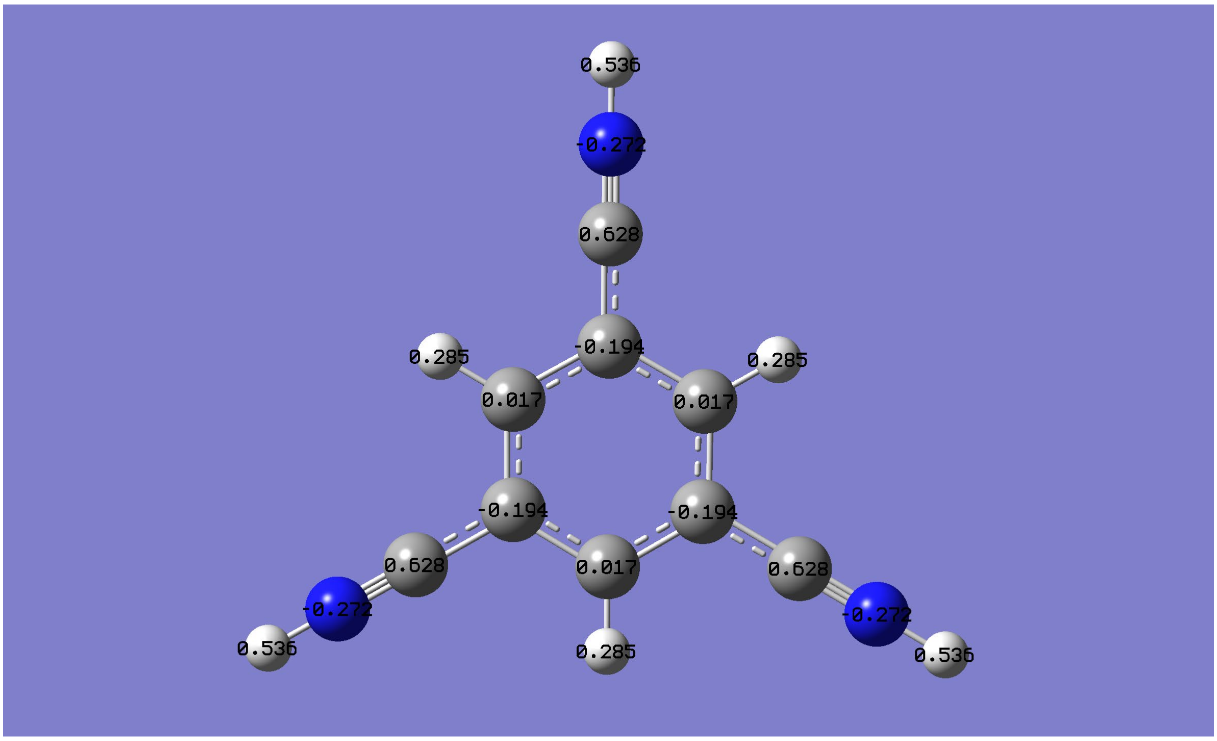

Figure 13. NPA charges of 1,3,5-$C_6H_3(CNH)_3$.

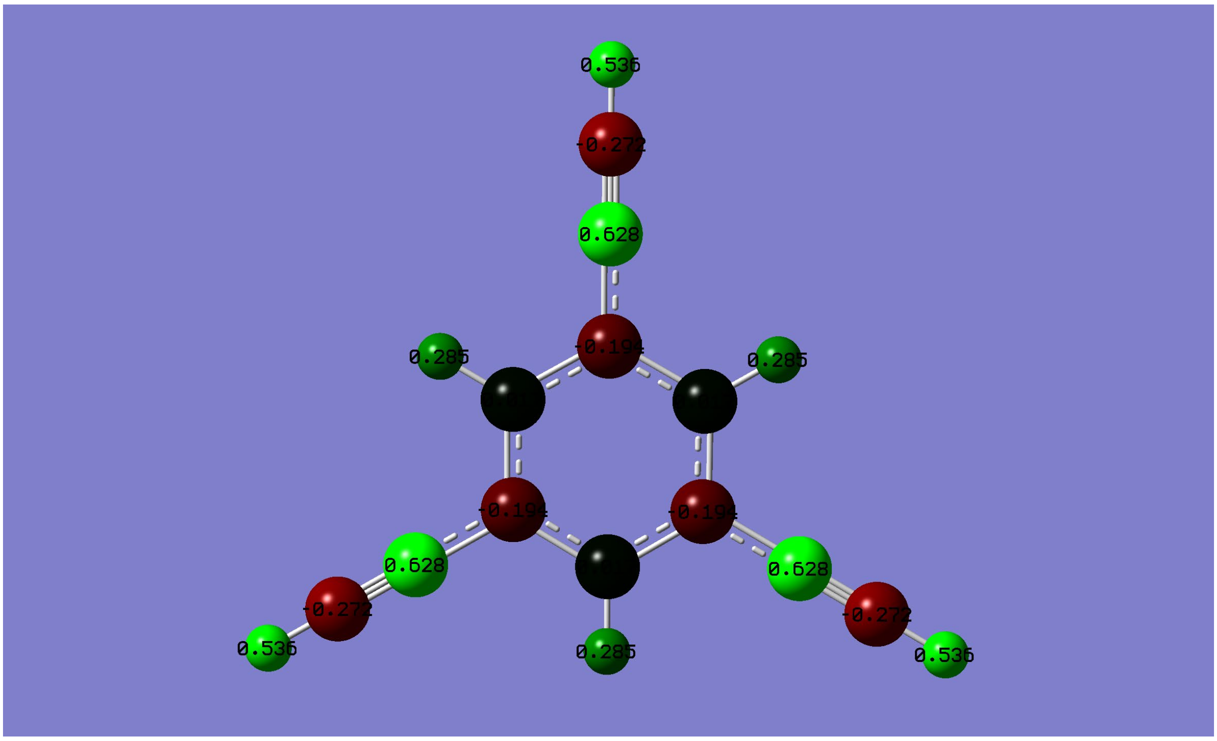

Figure 14. NPA charges of 1,3,5-$C_6H_3(CNH)_3$ (coloured).

5.3 1,3,5-$C_6H_3(CNCH_3)_3$.

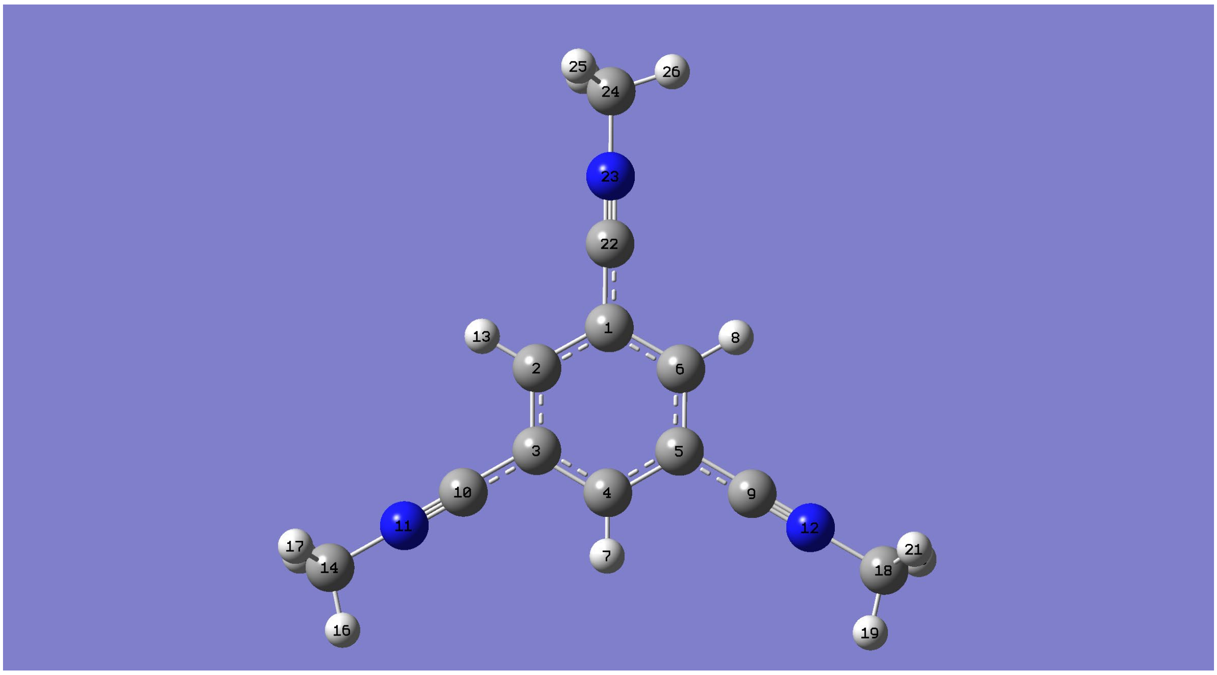

Figure 15. Optimized structure of 1,3,5-$C_6H_3(CNCH_3)_3$.

E(RB3LYP) = -627.612286787 Hartree

Standard orientation:

Center Number	Atomic Number	Atomic Type	Coordinates (Angstroms) X	Y	Z
1	6	0	-0.365993	1.353536	-0.000001
2	6	0	-1.366698	0.361965	-0.000003
3	6	0	-0.988758	-0.995179	-0.000008
4	6	0	0.370289	-1.366043	-0.000006
5	6	0	1.356586	-0.360154	-0.000003

6	6	0	0.998274	1.002289	-0.000002
7	1	0	0.652884	-2.409739	-0.000005
8	1	0	1.760960	1.768777	0.000000
9	6	0	2.744152	-0.727687	-0.000001
10	6	0	-2.001426	-2.012488	-0.000012
11	7	0	-2.814247	-2.829108	-0.000019
12	7	0	3.857907	-1.022811	0.000002
13	1	0	-2.411886	0.639110	-0.000001
14	6	0	-3.840799	-3.855343	0.000027
15	1	0	-4.448712	-3.729839	-0.896536
16	1	0	-3.346047	-4.827010	-0.003859
17	1	0	-4.443966	-3.734554	0.900436
18	6	0	5.261941	-1.391128	0.000004
19	1	0	5.324846	-2.479694	0.000181
20	1	0	5.722651	-0.979508	-0.898578
21	1	0	5.722725	-0.979213	0.898413
22	6	0	-0.741509	2.738963	0.000002
23	7	0	-1.042830	3.851057	0.000005
24	6	0	-1.425878	5.251144	0.000003
25	1	0	-2.013234	5.444174	0.898272
26	1	0	-0.514605	5.849906	0.000453
27	1	0	-2.012490	5.444379	-0.898708

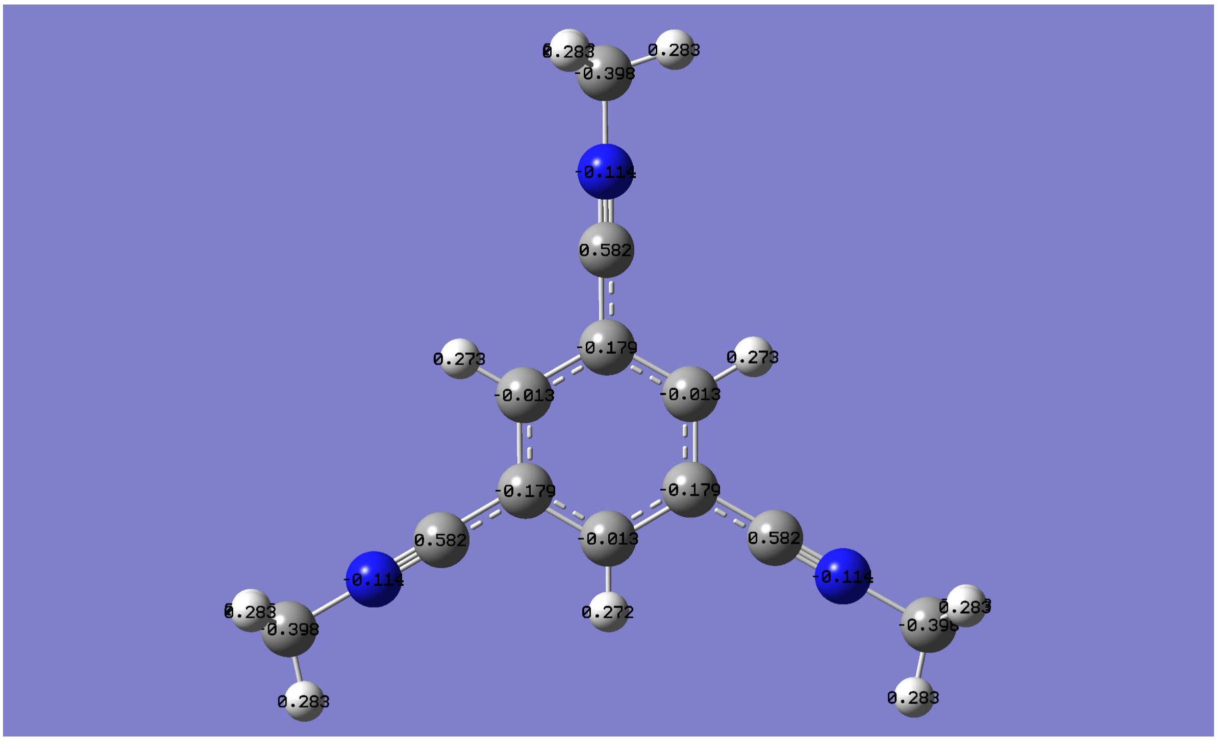

Figure 16. NPA charges of 1,3,5-$C_6H_3(CNCH_3)_3$.

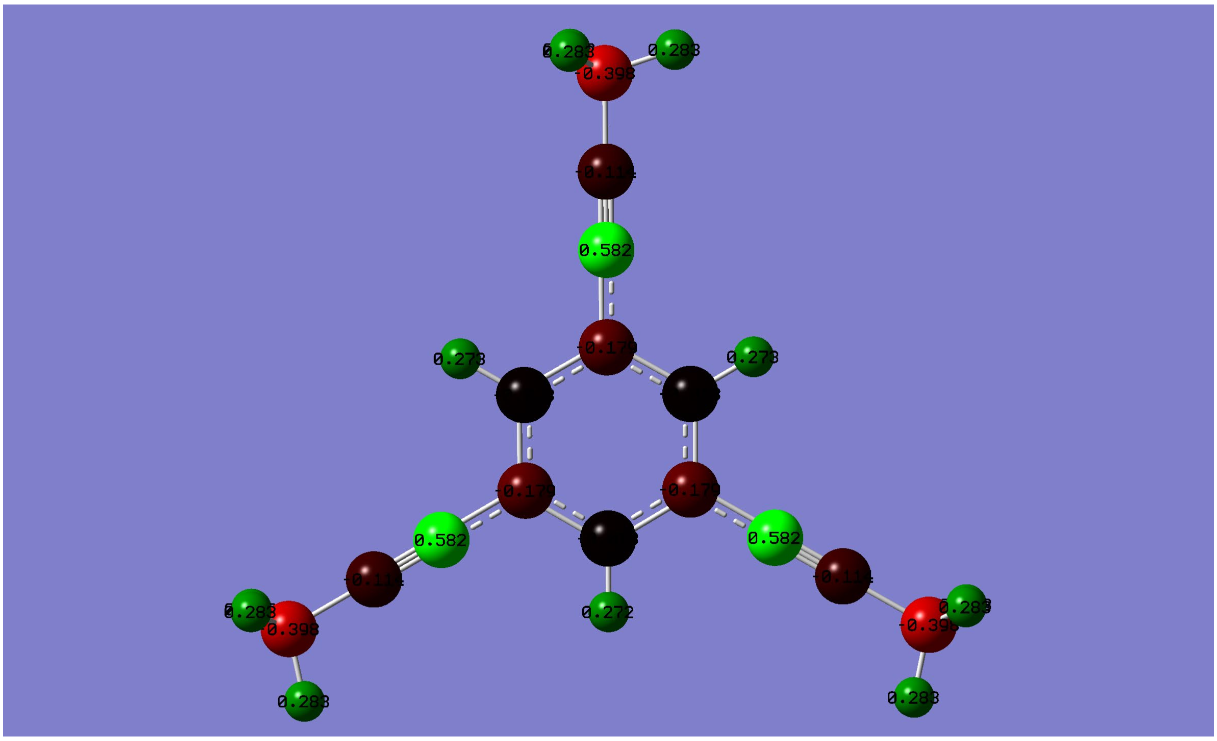

Figure 17. NPA charges of 1,3,5-$C_6H_3(CNCH_3)_3$ (coloured).

5.4 1,3,5-Triaminobenzene.

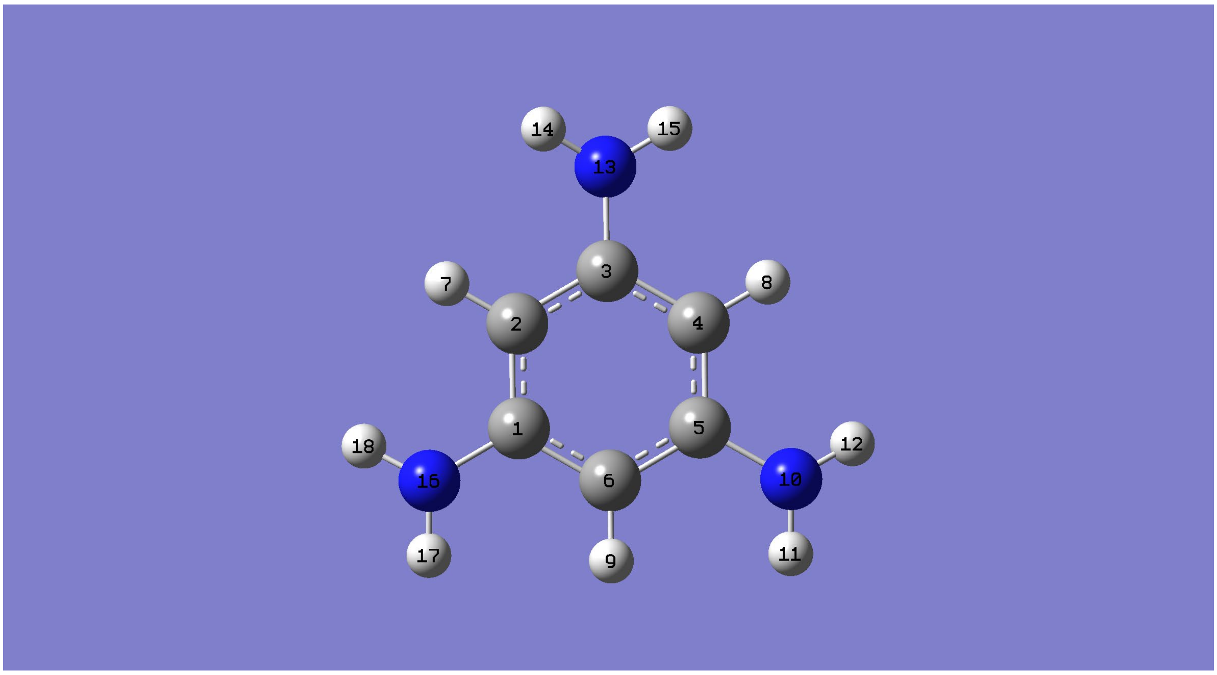

Figure 18. Optimized structure of 1,3,5-Triaminobenzene.

E(RB3LYP) = -398.360959689 Hartree

Standard orientation:

Center Number	Atomic Number	Atomic Type	Coordinates (Angstroms) X	Y	Z
1	6	0	0.638201	1.249702	-0.005753
2	6	0	-0.764712	1.179949	0.004114
3	6	0	-1.401255	-0.072094	0.007690
4	6	0	-0.639571	-1.252199	0.003970
5	6	0	0.763091	-1.177606	-0.005914

6	6	0	1.404188	0.072247	-0.010316
7	1	0	-1.353077	2.087794	0.007523
8	1	0	-1.131651	-2.215636	0.007253
9	1	0	2.484342	0.127831	-0.033955
10	7	0	1.524769	-2.353762	-0.045027
11	1	0	2.507343	-2.303537	0.158853
12	1	0	1.081632	-3.227454	0.178345
13	7	0	-2.801209	-0.144136	0.050410
14	1	0	-3.334291	0.678253	-0.172067
15	1	0	-3.247058	-1.016918	-0.172010
16	7	0	1.275127	2.497841	-0.044654
17	1	0	2.257660	2.548707	0.159270
18	1	0	0.744638	3.321365	0.178939

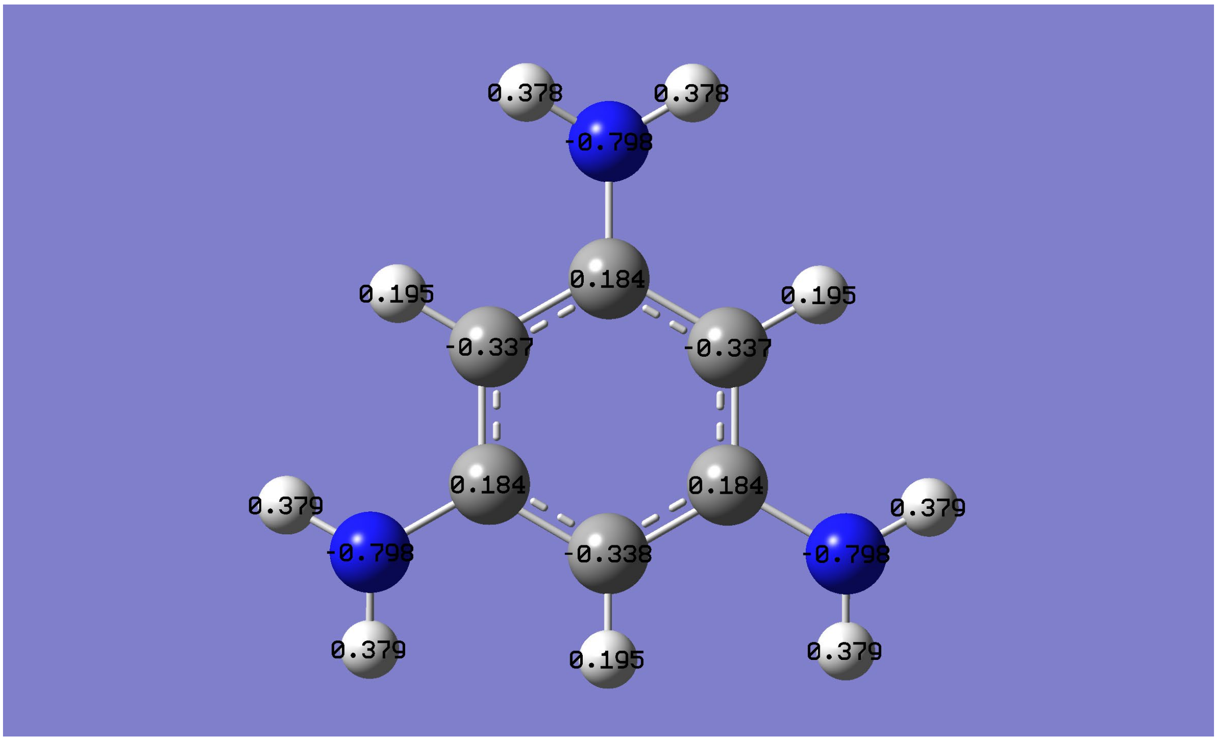

Figure 19. NPA charges of 1,3,5-Triaminobenzene.

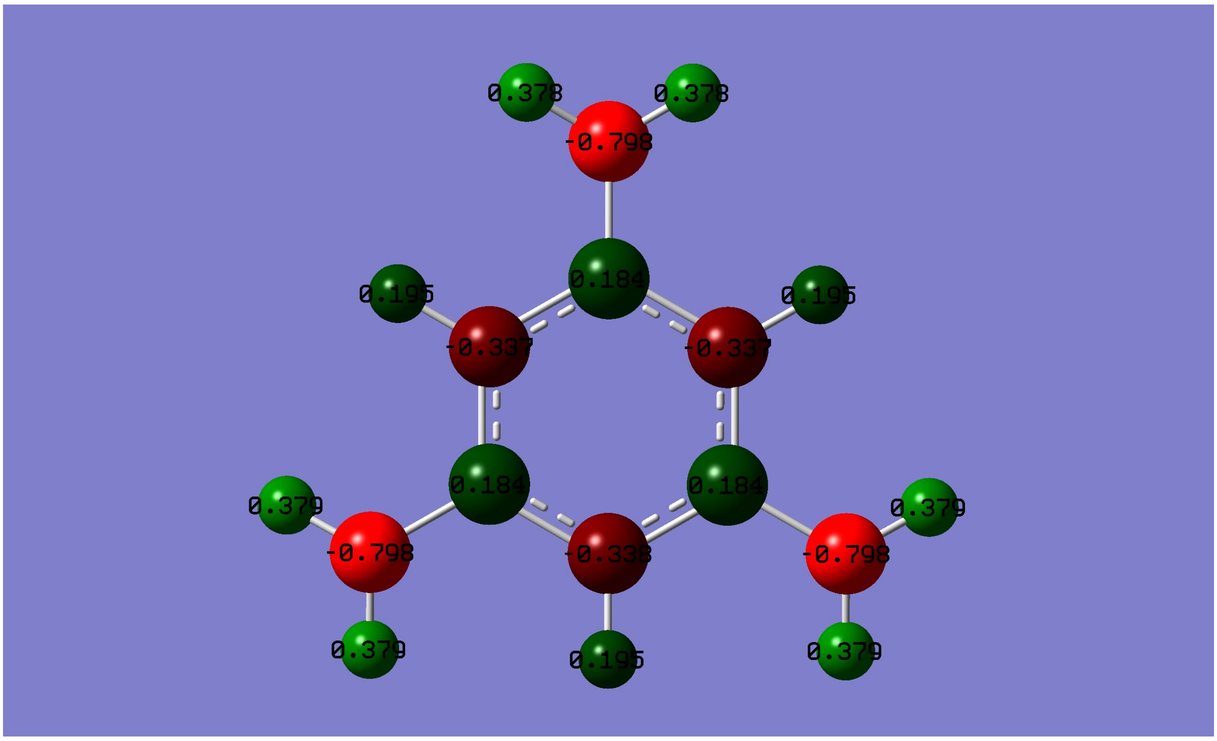

Figure 20. NPA charges of 1,3,5-Triaminobenzene (coloured).

5.5 1,3,5-$C_6H_3(NH_3)_3$.

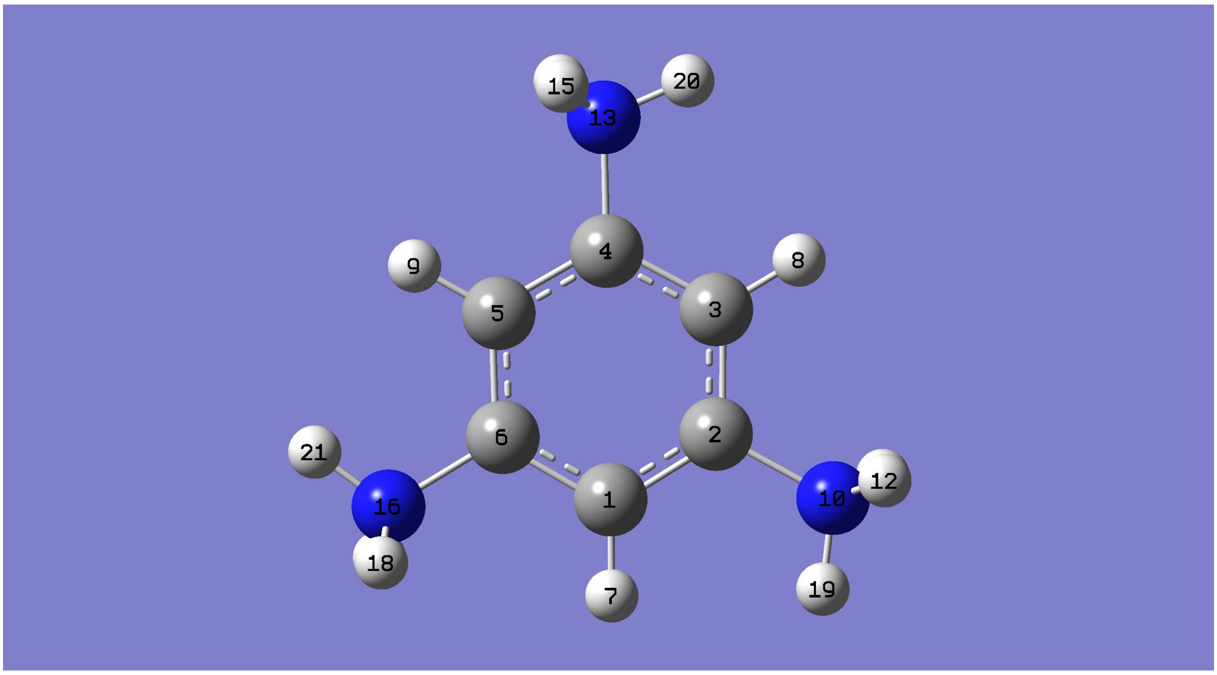

Figure 21. Optimized structure of 1,3,5-$C_6H_3(NH_3)_3$.

E(RB3LYP) = -399.043053094 Hartree

Standard orientation:

Center Number	Atomic Number	Atomic Type	Coordinates (Angstroms) X	Y	Z
1	6	0	-0.038291	-1.411228	-0.000054
2	6	0	1.177408	-0.726609	0.000007
3	6	0	1.241306	0.672448	0.000067
4	6	0	0.040552	1.382973	0.000049
5	6	0	-1.203015	0.738778	-0.000009

6	6	0	-1.217982	-0.656368	-0.000067
7	1	0	-0.071837	-2.494145	-0.000100
8	1	0	2.195931	1.184831	0.000112
9	1	0	-2.124065	1.309303	-0.000022
10	7	0	2.461760	-1.505398	0.000017
11	1	0	3.039975	-1.306580	-0.831691
12	1	0	3.040019	-1.306481	0.831672
13	7	0	0.072844	2.884635	0.000121
14	1	0	-0.388503	3.286028	-0.831530
15	1	0	-0.388478	3.285944	0.831832
16	7	0	-2.534601	-1.379236	-0.000131
17	1	0	-2.651429	-1.979453	-0.831812
18	1	0	-2.651531	-1.979409	0.831570
19	1	0	2.301182	-2.522638	0.000076
20	1	0	1.034103	3.254161	0.000105
21	1	0	-3.335252	-0.731537	-0.000215

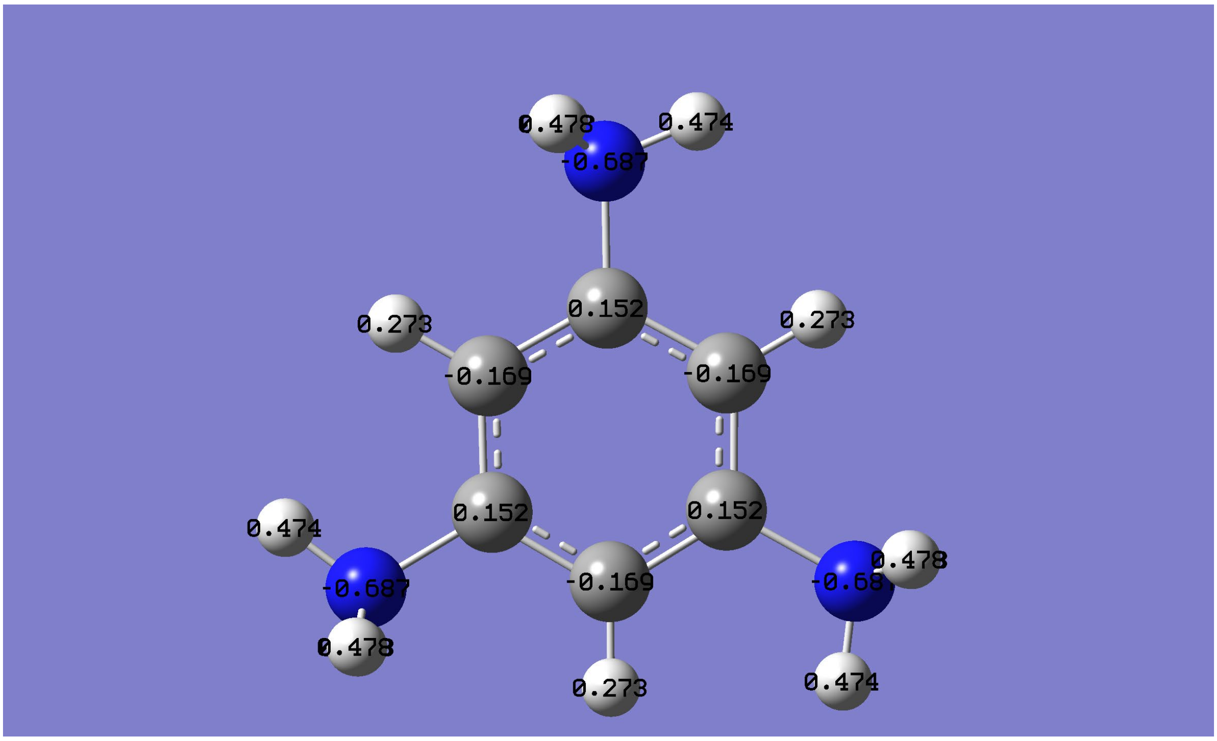

Figure 22. NPA charges of 1,3,5-$C_6H_3(NH_3)_3$.

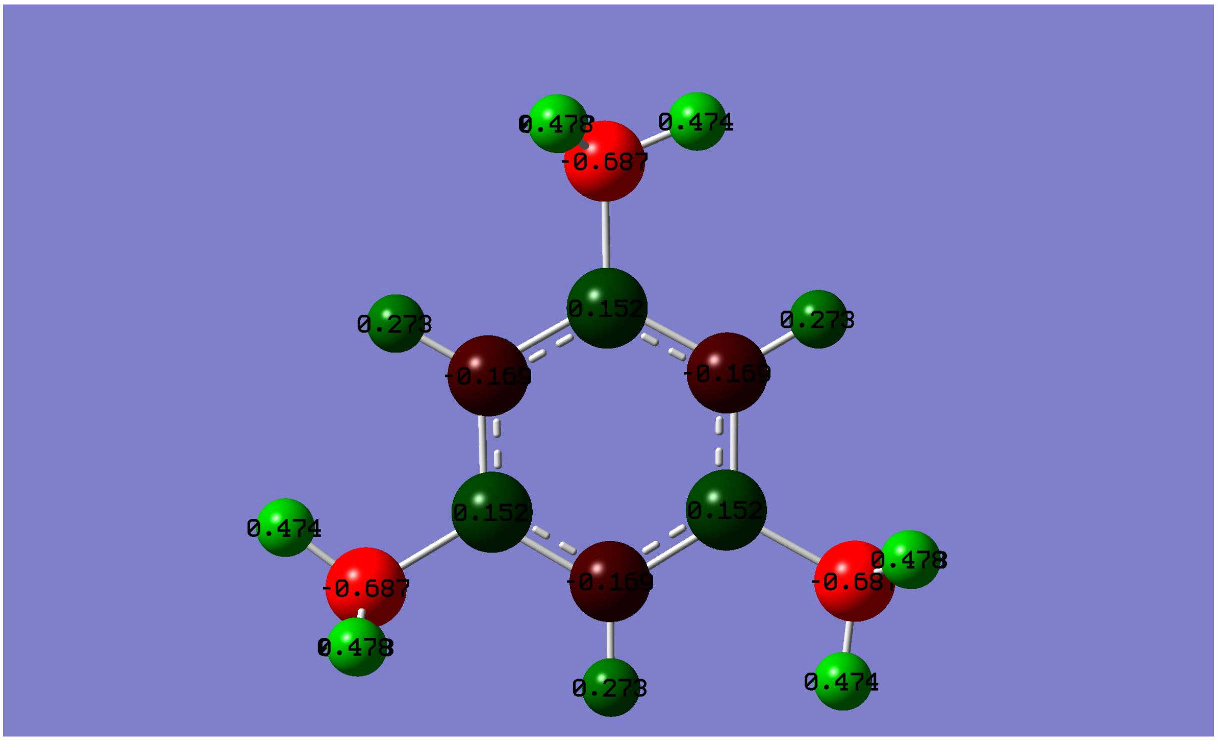

Figure 23. NPA charges of 1,3,5-$C_6H_3(NH_3)_3$ (coloured).

5.6 Comparison of NPA charges

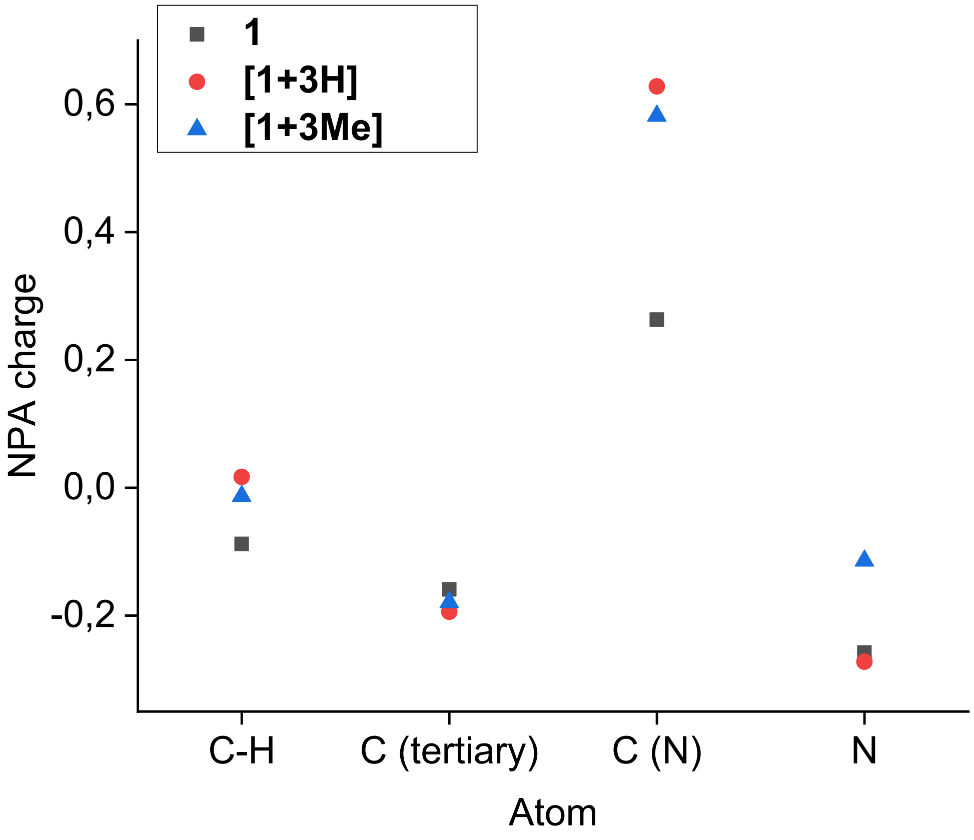

Figure 24. NPA charges of 1,3,5-Tricyanobenzene, its` protonated and methylated derivate (R= aromatic core).

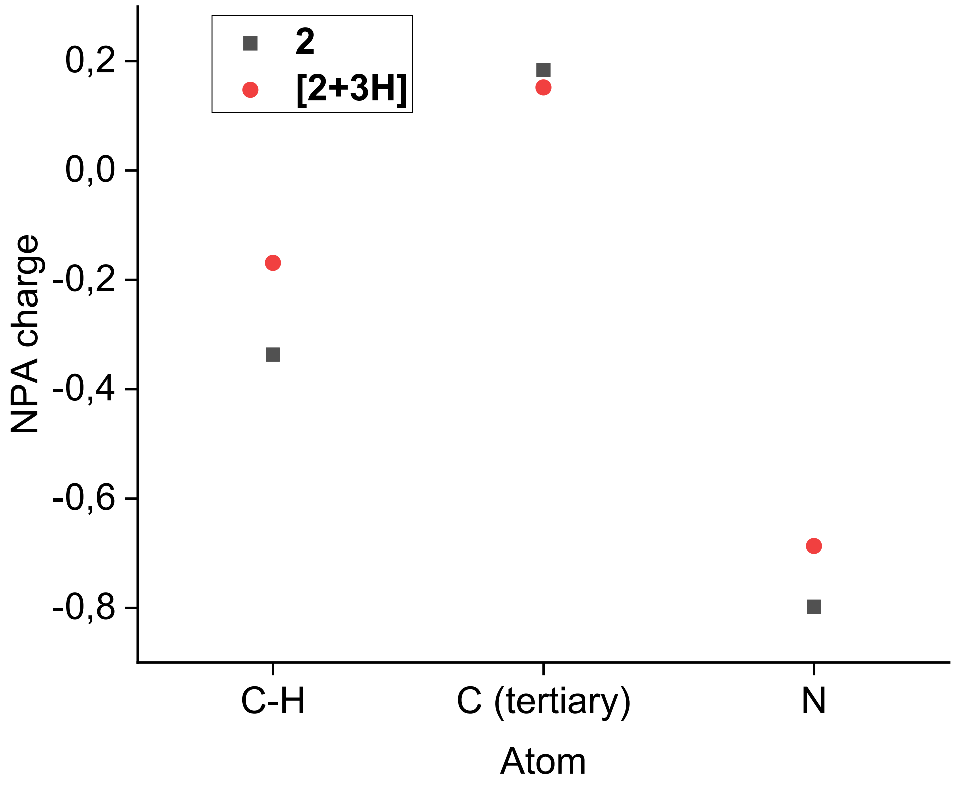

Figure 25. NPA charges of 1,3,5-Triaminobenzene and its` protonated derivate (R= aromatic core).

5.7 NMR shieldings

Table 20. Comparison of NMR data of 1,3,5-Tricyanobenzene and 1,3,5-Triammoniumbenzene (135-TAB) and its` protonated and methylated derivatives with calculated ^{13}C NMR shifts in square brackets.[a]

[ppm]	^{13}C starting material in SO_2 @RT	^{13}C protonated product in HF @0° C (Δ to starting material)	^{13}C methylated product in SO_2 @–20° C (Δ to starting material)
**C**N	111.17 [118]	100.08 (–11.09) [100]	100.90 (–10.27) [102]
**C**CN	113.78 [117]	109.13 (–4.65) [109]	111.25 (–2.53) [112]
**C**-H	141.07 [141]	150.39 (+9.32) [158]	150.09 (+9.02) [153]
N-**C**H_3			33.97 [35]
	^{13}C 135-TAB+3H^+ in *a*HF		
**C**N	132.07 [134]		
**C**-H	121.95 [128]		

[a] ^{13}C NMR shielding with TMS optimized as reference, calculated on the same level as NICS(0).

6. Literature

[1] *MestReNova 12.0.2*; Mestrelab Research S.L., **2018**.

[2] Rigaku Oxford Diffraction, CrysAlisPro Software System, *Version 1.171.38.46*, Rigaku Corporation, Oxford, UK, **2015**.

[3] G. M. Sheldrick, *Acta Crystallogr. A* **2015**, *71*, 3. DOI: 10.1107/S2053273314026370.

[4] G. M. Sheldrick, *Acta Crystallogr. C* **2015**, *71*, 3. DOI: 10.1107/S2053229614024218.

[5] L. J. Farrugia, *J. Appl. Crystallogr.* **1999**, *32*, 837. DOI: 10.1107/S0021889899006020.

[6] A. L. Spek, *J. Appl. Crystallogr.* **2003**, *36*, 7. DOI: 10.1107/S0021889802022112.

[7] *SCALE3 ABSPACK*, *An Oxford Diffraction Program*, Oxford Diffraction Ltd, UK, **2005**.

[8] *Mercury 2020.2.0 (Build 290188)*, CCDC, **2020.**

[9] *Gaussian 16, Revision A.03*, M. J. Frisch, G. W. Trucks, H. B. Schlegel, G. E. Scuseria, M. A. Robb, J. R. Cheeseman, G. Scalmani, V. Barone, G. A. Petersson, H. Nakatsuji, X. Li, M. Caricato, A. V. Marenich, J. Bloino, B. G. Janesko, R. Gomperts, B. Mennucci, H. P. Hratchian, J. V. Ortiz, A. F. Izmaylov, J. L. Sonnenberg, D. Williams-Young, F. Ding, F. Lipparini, F. Egidi, J. Goings, B. Peng, A. Petrone, T. Henderson, D. Ranasinghe, V. G. Zakrzewski, J. Gao, N. Rega, G. Zheng, W. Liang, M. Hada, M. Ehara, K. Toyota, R. Fukuda, J. Hasegawa, M. Ishida, T. Nakajima, Y. Honda, O. Kitao, H. Nakai, T. Vreven, K. Throssell, J. A. Montgomery, Jr., J. E. Peralta, F. Ogliaro, M. J. Bearpark, J. J. Heyd, E. N. Brothers, K. N. Kudin, V. N. Staroverov, T. A. Keith, R. Kobayashi, J. Normand, K. Raghavachari, A. P. Rendell, J. C. Burant, S. S. Iyengar, J. Tomasi, M. Cossi, J. M. Millam, M. Klene, C. Adamo, R. Cammi, J. W. Ochterski, R. L. Martin, K. Morokuma, O. Farkas, J. B. Foresman, and D. J. Fox, Gaussian, Inc., Wallingford CT, **2016**.

[10] D. S. Reddy, K. Panneerselvam, G. R. Desiraju, H. L. Carrell, C. J. Carell, *Acta Cryst. C* **1995**, *51*, 2352–2354.

[11] H. Friebolin in *Ein- und Zweidimensionale NMR-Spektroskopie*, Wiley-VCH, Weinheim, **2013**, pp. 106–107.

[12] T. Yamaoka, H. Hosoya, S. Nagakura, *Tetrahedron* **1968**, *24*, 6203–6213.
